# Spirobarbiturates with a pyrrolizidine moiety: synthesis, structure and biological evaluation

**DOI:** 10.3762/bjoc.22.20

**Published:** 2026-02-17

**Authors:** Arthur A Puzyrkov, Andrew S Drachuk, Ekaterina A Popova, Alexander V Stepakov, Vitali M Boitsov

**Affiliations:** 1 Saint Petersburg State University, Universitetskaya nab. 7/9, 199034, St. Petersburg, Russian Federation; 2 Saint Petersburg National Research Academic University of the Russian Academy of Sciences, ul. Khlopina 8/3, 194021, St. Petersburg, Russian Federation; 3 Saint Petersburg State Pediatric Medical University, Litovskaya ul. 2, 194353, St. Petersburg, Russian Federation; 4 Saint Petersburg State Institute of Technology (Technical University), Moskovsky prospect 26, 190013, St. Petersburg, Russian Federation

**Keywords:** alloxan, antiproliferative activity, azomethine ylides, 1,3-dipolar cycloaddition, maleimides, pyrrolizidine moiety, spirobarbiturates, three-component reactions

## Abstract

Polycyclic spirobarbiturates containing a pyrrolizidine moiety were synthesized via a one-pot three-component 1,3-dipolar cycloaddition reaction of alloxan, ʟ-proline and *N*-substituted maleimides. The reaction stereoselectivity was found to depend on the nature of substituents in the maleimide: in most cases the *endo*-isomer is the main (or only) product, but for some *N*-arylmaleimides the *exo*-product is predominant. The proposed mechanism is discussed, and the products were characterized by detailed spectral analysis. The configuration of the stereocenters was determined by X-ray diffraction analysis (XRD) for two adducts, followed by Hirshfeld surface analysis. The antiproliferative effect of the synthesized compounds against cancer cell lines was assessed.

## Introduction

**Scheme 1:**
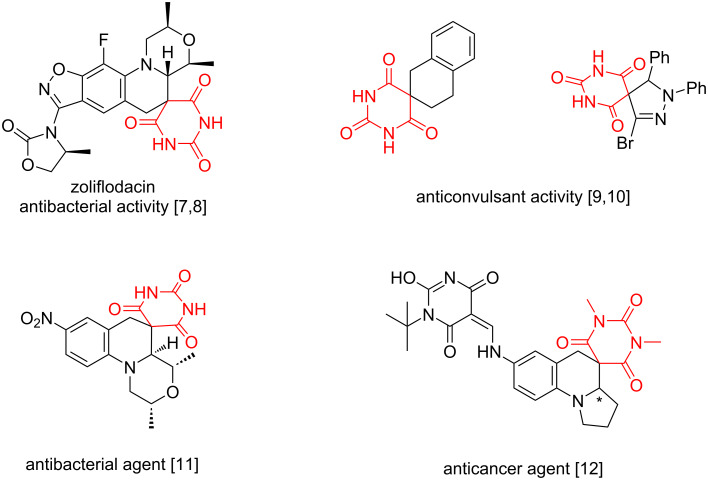
Biologically active compounds with a spirobarbiturate moiety in their structure [7–12].

The history of barbiturates dates back to 1863, when the young Adolf von Baeyer first synthesized barbituric acid [1–2]. In 1903, the drug marketed under the trade name barbital (veronal^©^) became commercially available as a sedative [3]. For more than 120 years of barbiturate history a lot has happened. Barbituric acid-based medications were widely used until the 40–50s of the last century. Then, due to accumulating evidence of their addictive potential and adverse effects, most of these substances were eventually phased out of pharmaceutical use. Today, only slightly more than a dozen barbiturate-containing drugs remain in medical practice [4]. Nevertheless, interest in barbiturate derivatives resurged around the 1920s, when the first compounds containing a spirobarbiturate moiety were synthesized [5–6]. By the early 21st century, this research culminated in the approval of the first drug whose active molecule contained a spirobarbiturate scaffold – zoliflodacin^©^ (Scheme 1) [7].

Due to the significance of spirobarbiturates, numerous approaches have been devised for their synthesis [13–19]. Advances in this area of organic synthesis include the preparation of barbiturates containing spiro-fused cyclohexane, cyclopentane, tetrahydrooxepine, and tetrahydroquinoline moieties via [3 + 2], [4 + 2], and [5 + 2] annulation reactions involving arylidene and alkylidene barbiturates [20–23]. In 2018, Kota and Sreevani prepared spirobarbiturates via a Mo(CO)_6_-catalyzed intermolecular [2 + 2 + 2] cycloaddition of propargyl halides with dipropargylbarbituric acid [24]. Almeida and colleagues described the formation of spiroindoline barbiturates via thermal rearrangement of 2,1-benzisoxazole barbiturates [25]. Other methods include the DBU-mediated stereospecific cyclopropanation of barbiturate-based olefins with benzyl chlorides, developed by Chang’s group for the synthesis of spirobarbiturate-cyclopropanes [26].

**Scheme 2:**
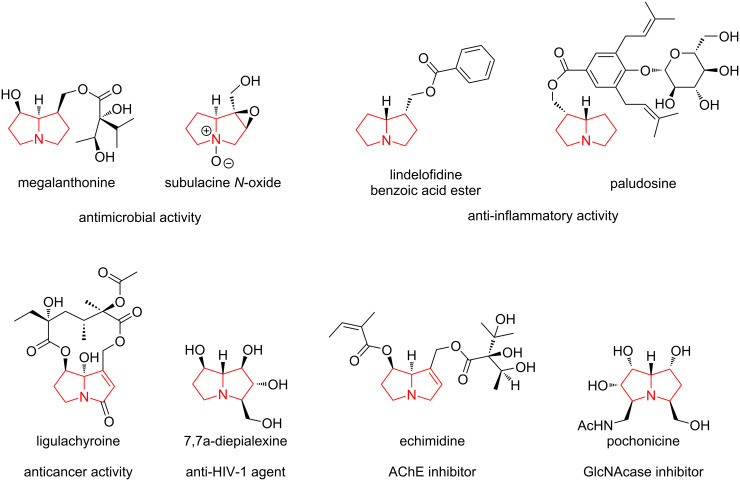
Biologically active alkaloids with a pyrrolizidine moiety.

Another interesting structural fragment is the pyrrolizidine heterocycle, which is a system of two condensed pyrrolidine rings sharing a common nitrogen atom. This heterocyclic system occurs naturally in numerous plant-derived alkaloids [27–30]. Notably, most pyrrolizidine alkaloids exhibit severe hepatotoxicity, genotoxicity and cause neurological disorders in humans and animals [31–33]. However, some representatives of these compounds (Scheme 2) find applications in pharmacology [34] due to their antimicrobial (megalanthonine; subulacine *N*-oxide) [35–36], anti-inflammatory (lindelofidine, benzoic acid ester; paludosine) [37–38], anticancer (ligulachyroine) [39] and antiviral activities (7,7a-diepialexine) [40]. Some pyrrolizidine derivatives are inhibitors of enzymes such as acetylcholinesterase (AChE) (echimidine) [41] or β*-N-*acetylglucosaminidase (GlcNAcases) (pochonicine) [42].

**Scheme 3:**
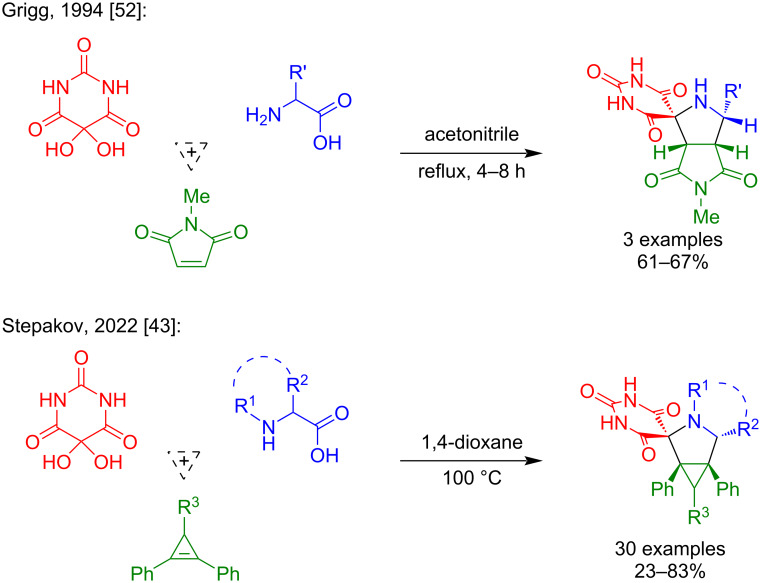
Previous studies on the three-component synthesis of spirobarbiturates.

Our previous studies have shown that spiro-fused barbiturates containing azabicyclo[3.1.0]hexane and cyclopropa[*a*]pyrrolizine moieties can be synthesized via 1,3-dipolar cycloaddition between cyclopropene derivatives and azomethine ylides generated in situ from alloxan and α-amino acids [43]. In [43], a mechanism was proposed for the formation of azomethine ylides from alloxan and α-amino acids, which includes a stage of decarboxylation of the intermediate lactone. It is worth noting that generating azomethine ylides via decarboxylation of α-amino acid derivatives is a common practice in synthetic chemistry [44–46]. It was shown in our recent works that spirocyclopropa[*a*]pyrolizines as well as spiroazabicyclo[3.1.0]hexanes are readily available from cyclopropenes and azomethine ylides [47–49] and some adducts inhibited cancer cell growth in vitro [50–51]. The first reported synthesis of spirobarbiturates employing azomethine ylides (generated in situ from alloxan and α-amino acids) reacted with *N*-methylmaleimide was published by Ronald Grigg’s group in 1994 (Scheme 3) [52]. The authors reported the synthesis of spirobarbiturates containing various substituents: R’ = iPr (67%), Bn (61%), and Ph (66%).

In this work, which is a continuation of studies on the synthesis of spirobarbiturates via a [3 + 2] cycloaddition reaction of azomethine ylides, we present for the first time a three-component diastereoselective synthesis of hybrid systems containing spiro-fused cyclic fragments of barbituric acid and pyrrolo[3,4-*a*]pyrrolizidine. It was found that azomethine ylide, which is generated in situ by condensation of alloxan and ʟ-proline, can react with maleimides to give spirobarbiturate-pyrrolo[3,4-*a*]pyrrolizidine-1,3-diones in moderate to good yields. We have demonstrated the possibility of using a wide range of *N*-substituted maleimides as dipolarophiles. All obtained spirocyclic adducts became objects of biological research.

## Results and Discussion

**Scheme 4:**
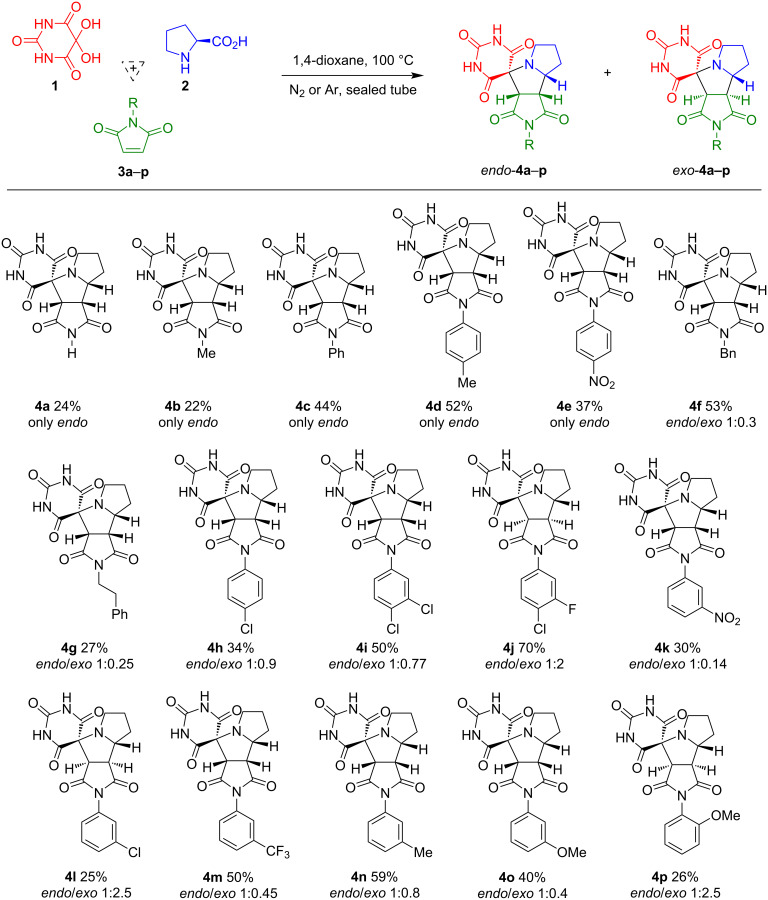
Synthesis of racemic spirobarbiturates **4a**–**p** via one-pot three-component reaction of alloxan (**1**), ʟ-proline (**2**) and *N*-substituted maleimides **3**.

The 1,3-dipolar cycloaddition between alloxan (**1**), ʟ-proline (**2**), and maleimides **3a**–**p** afforded spirobarbiturates **4a**–**p** as diastereomeric racemic mixtures with 22–70% yields (Scheme 4). The reaction progress is highly sensitive to water presence in the system. It is worth noting that in all cases, a side reaction was observed between alloxan (**1**) and α-amino acid **2**, leading to the formation of murexide via Strecker degradation (Scheme S1, Supporting Information File 1) [53]. This side reaction resulted in decreased yields of the target products. We observed that murexide formation decreased when the reaction was carried out in anhydrous 1,4-dioxane under an inert atmosphere at 100 °C. When using dry acetonitrile as a solvent, the reaction yields were lower, so we decided to use a higher-boiling solvent – 1,4-dioxane.

It was established that both diastereoselectivity and reaction yields significantly depend on the *N*-substituent of the maleimide: exclusive formation of *endo*-products **4a**–**e** (yields up to 52%) was observed when using maleimides with R = H (**3a**), Me (**3b**), Ph (**3c**), *p*-Tol (**3d**), or 4-NO_2_С_6_H_4_ (**3e**) at the nitrogen atom. Maleimides bearing aromatic halogens (**3h**, **3i**, **3j**, **3l**) or R = Bn (**3f**), -CH_2_CH_2_Ph (**3g**), 3-NO_2_С_6_H_4_ (**3k**), and 3-CF_3_С_6_H_4_ (**3m**) groups yielded mixtures of *exo*- and *endo*-adducts. When the reaction was performed with a maleimide containing a chlorine atom at the *para*-position, a near 1:1 mixture of *endo*- and *exo*-isomers **4h** (34%) was obtained. For the *meta*-chloro-substituted maleimide, the *exo*-isomer **4l** (25%) predominated (*endo*/*exo* 1:2.5). In contrast, the maleimide bearing two chlorine atoms at the *para*- and *meta*-positions preferentially formed the *endo*-isomer **4i** (50%, *endo*/*exo* 1:0.77). Substitution of the *meta*-chlorine with fluorine also favored the *exo*-isomer (compound **4j**, 70%, *endo*/*exo* 1:2), similar to **4l**. The presence of an electron-withdrawing group at the *meta*-position of the maleimide’s *N*-aryl group likely facilitates *exo*-isomer formation. Reactions with maleimides containing *meta*-NO_2_ and *meta*-CF_3_ groups were investigated to test this hypothesis. Contrary to expectations, the *endo*-isomer predominated in these cases (**4k**, 30%, *endo*/*exo* 1:0.14; **4m**, 50%, *endo*/*exo* 1:0.45). We also synthesized two spirobarbiturates, **4n** (59%) and **4o** (40%), from maleimides containing electron-donating groups at the *meta*-position of the aromatic ring. In both cases, the *endo*-isomers predominated among the cycloadducts. However, using an *N*-arylmaleimide with an *ortho*-methoxy group, we obtained product **4p** (26%) as diastereomeric mixture, and the *exo*-isomer became the major product. Notably, the ^1^H NMR spectrum of product **4p** exhibited three doublet signals corresponding to the methine proton in a five-membered ring formed during cycloaddition. This observation indicated the formation of atropisomers in the 1,3-dipolar cycloaddition of *N*-(2-methoxyphenyl)maleimide (**3p**) which was previously reported by Awad I. Said et al. [54].

The cycloaddition reactions were performed in sealed tubes under an inert atmosphere at 100 °C in anhydrous 1,4-dioxane with constant stirring for 6–16 h. Reaction progress was monitored by TLC (CH_2_Cl_2_/MeOH 25:1) and the spirobarbiturates were isolated from the reaction mixture by preparative TLC or column chromatography. Notably, compounds **4a**, **4e**, and **4k** were purified by crystallization. Detailed synthetic and isolation procedures for all compounds are provided in Supporting Information File 1. We regret to state that when forming a mixture of diastereomers, we were unable to isolate individual products either by chromatography or by recrystallization.

**Scheme 5:**
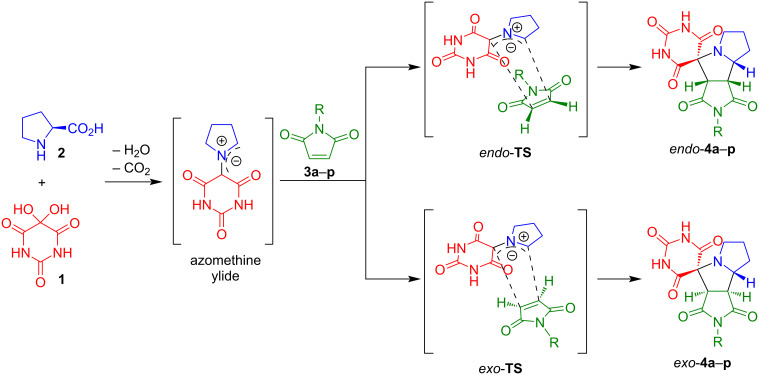
A plausible mechanism of spirobarbiturate formation from alloxan (**1**), ʟ-proline (**2**), and *N*-substituted maleimides **3a**–**p**.

The proposed reaction mechanism is shown in Scheme 5, where it can be seen that the intermediate azomethine ylide, acting as a 1,3-dipole, undergoes a [3 + 2] cycloaddition with *N*-substituted maleimides **3a**–**p**. The formation of azomethine ylide from alloxan (**1**) and ʟ-proline (**2**) was previously studied using DFT calculations [43]. Based on the calculated data, it was assumed that the 1,3-dipole is formed as a result of a multistage sequence: initial generation of a zwitterionic imine intermediate from **1** and **2**, followed by intramolecular cyclization to yield a lactone, which undergoes decarboxylation to produce the corresponding azomethine ylide. In our system, we propose this generated ylide reacts with *N*-substituted maleimides **3a**–**p** such that the transformation predominantly proceeds via the *endo*-TS, leading to preferential formation of the *endo*-adduct (upper pathway). The predominance of *exo*-isomers in some cases probably reflects insufficient stabilization due to secondary orbital interactions in the transition state [43].

It should be noted separately that secondary cyclic amino acids such as azetidine-2-carboxylic acid, thiazolidinecarboxylic acid, and pipecolic acid, in combination with alloxan (**1**) and maleimides **3**, did not lead to the formation of the corresponding cycloadducts. In this work, we also used other dipolarophiles. Under the standard reaction conditions (1,4-dioxane, heating with alloxan and ʟ-proline), tetracyanoethylene, chalcone, and dimethyl acetylenedicarboxylate all failed to produce the target spiro adducts – chalcone and dimethyl acetylenedicarboxylate showed no reactivity, while tetracyanoethylene led to complete resinification.

The structure of the products, including the configuration of their stereocenters, was determined using NMR spectroscopy and XRD analysis. *endo*-Isomers **4a**–**e** exhibited the following characteristic signals in their ^1^H NMR spectra: multiplets in the range of δ 1.5–4.4 ppm were assigned to the protons of the pyrrolizidine ring system. The methyl singlets of **4b** and **4d** (δ 2.79 and 2.35 ppm, respectively) also fall within this range.

**Figure 1:**
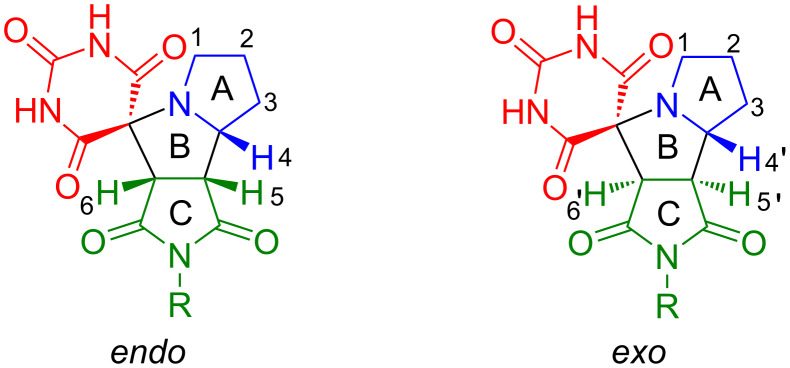
Schematic structures of *endo*- and *exo*-adducts of spirobarbiturates **4**.

The methylene protons of the five-membered ring A (Figure 1) in *endo*-isomers **4a**–**e** resonate as complex multiplets: C*H*_2_^2^ and C*H*_2_^3^ groups appear at δ 1.65–2.05 ppm, while C*H*_2_^1^ is deshielded (δ 2.54–2.70 ppm), consistent with the deshielding effect of the directly attached nitrogen atom on the C*H*_2_^1^ group. The methine protons resonate as distinct signals appearing as a ddd for C*H*^4^, dd for C*H*^5^, and a d for C*H*^6^. Chemical shift values, signal multiplicities, and spin–spin coupling constants for the methine protons of all compounds are provided in Supporting Information File 1. Notably, for compounds **4a** and **4b** containing non-aromatic substituents in the succinimide moiety, the signals of protons C*H*^4^ and C*H*^6^ overlap, whereas no such overlap occurs with aromatic substituents. The assignments of the methine proton signals for all compounds were established using ^1^H,^13^C HSQC NMR spectroscopy. The chemical shift values, signal shapes, and spin–spin coupling constants are provided in Table S1 in Supporting Information File 1. The aromatic protons of compounds **4c**–**e** appear in the downfield region of the spectrum. For product **4c**, the aromatic protons resonate as three distinct signals: a broad doublet at δ 7.26 ppm (*ortho*-protons), along with multiplets at δ 7.40–7.47 ppm (*para*-proton), and δ 7.48–7.56 ppm (*meta*-protons). In contrast, compounds **4d** and **4e**, which contain *para*-substituted aromatic rings, exhibit two characteristic doublets each: **4d** shows signals at δ 7.12 and 7.30 ppm for *ortho*- and *meta*-protons respectively (*J* = 8.2 Hz) and **4e** displays resonances at δ 7.58 and 8.41 ppm for *ortho*- and *meta*-protons (*J* = 8.8 Hz). In the ^1^H NMR spectra, the proton signals of ring A in diastereomers **4f**–**p** overlap and appear at the same chemical shifts as in the *endo*-isomers **4a**–**e**. The C*H*^4^–C*H*^6^ protons of diastereomers **4f**–**p** resonate as distinct separate signals. The formation of atropisomers **4p** is evidenced by three distinct doublets in the ^1^H NMR spectrum at δ 4.29 ppm (*J* = 8.8 Hz), 4.49 ppm (*J* = 10.3 Hz), and 4.58 ppm (*J* = 10.3 Hz), assigned to the diastereotopic *H*^6^/*H*^6^’ methine protons of different atropisomeric forms. The NH protons of the barbiturate ring resonate as broad singlets at δ 11.5–11.7 ppm, while compound **4a** shows an additional broad singlet at δ 11.30 ppm corresponding to the succinimide NH group.

In the ^13^C NMR spectra of *endo*-adducts **4a**–**e**, the carbon signals of ring A (Figure 1) appear at δ_C_ 44.8–45.0 ppm (*C*H_2_^1^), 27.3 ppm (*C*H_2_^2^), and 25.2 ppm (*C*H_2_^3^). Ring B carbons resonate at δ_C_ 67.7–67.8 ppm (*C*H^4^), 46.3–46.4 ppm (*C*H^5^), and 58.2–58.3 ppm (*C*H^6^). The carbonyl carbons of ring C and the barbiturate ring appear at δ_C_ 150.0, 169.3–169.4, 171.2–171.5, 175.5–176.8, and 176.4–177.9 ppm. The spiro carbon appears at δ_C_ 70.1–70.3 ppm. For diastereomers **4f**–**p**, the ^13^C NMR spectra proved more informative than the ^1^H NMR spectra, clearly displaying a duplicate set of nearly all signals. The chemical shifts of carbons in rings A, B, and C for *endo*-**4f**–**p** could be unambiguously identified, as they remain virtually unchanged compared to those in the pure *endo*-isomers **4a**–**e**.

^19^F NMR analysis of **4j** revealed two fluorine signals (δ_F_ −114.3 ppm for *exo*, −114.4 ppm for *endo*), while the CF_3_ group in **4m** showed analogous splitting (δ_F_ −61.2 ppm *endo*, −61.3 ppm *exo*).

The structures of all cycloadducts **4a**–**p** were confirmed by ^13^C DEPT NMR and two-dimensional NMR spectroscopy (HSQC, COSY), supplemented by HRESIMS. Complete spectral data are provided in Supporting Information File 1.

**Figure 2:**
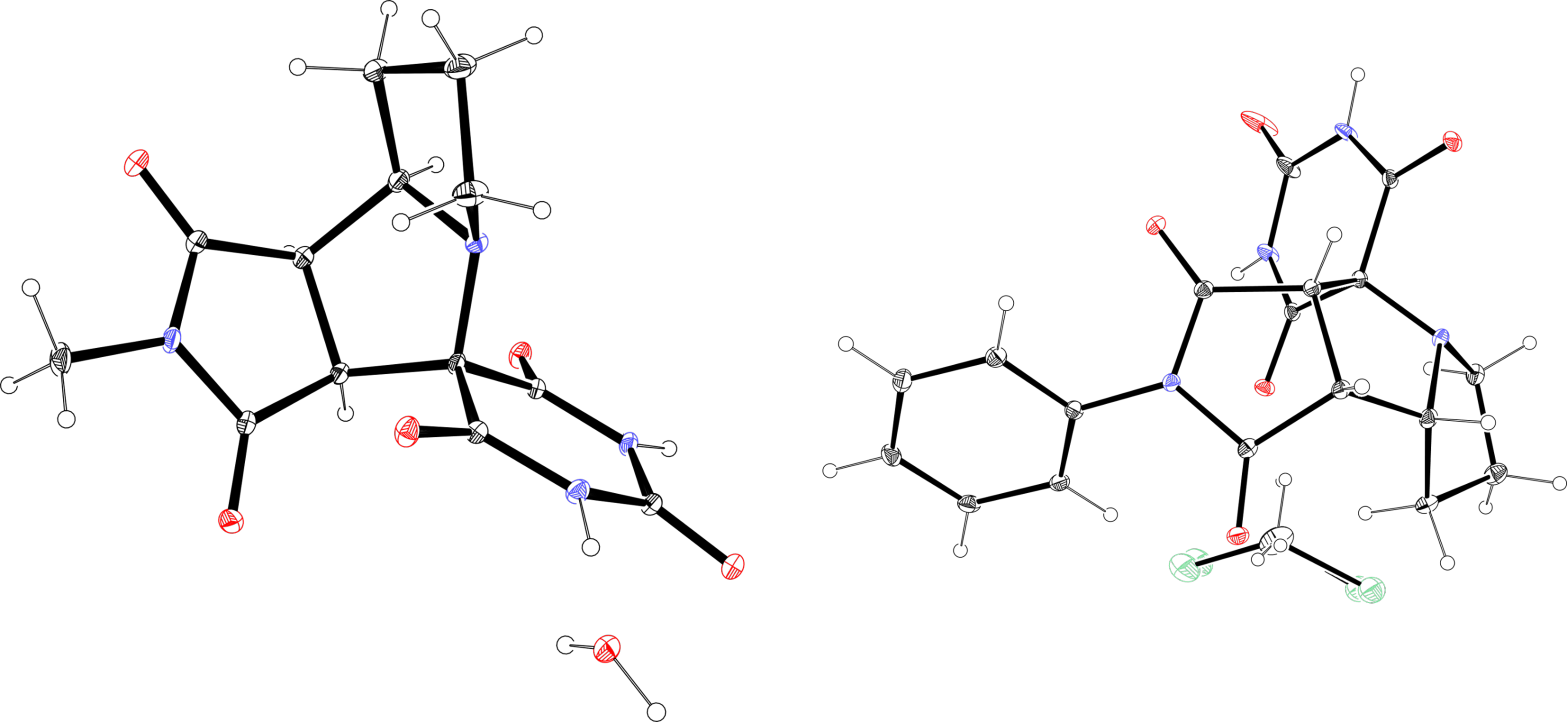
X-ray crystal structures of compounds **4b** (CCDC 2391172, left) and **4c** (CCDC 2391171, right).

**Figure 3:**
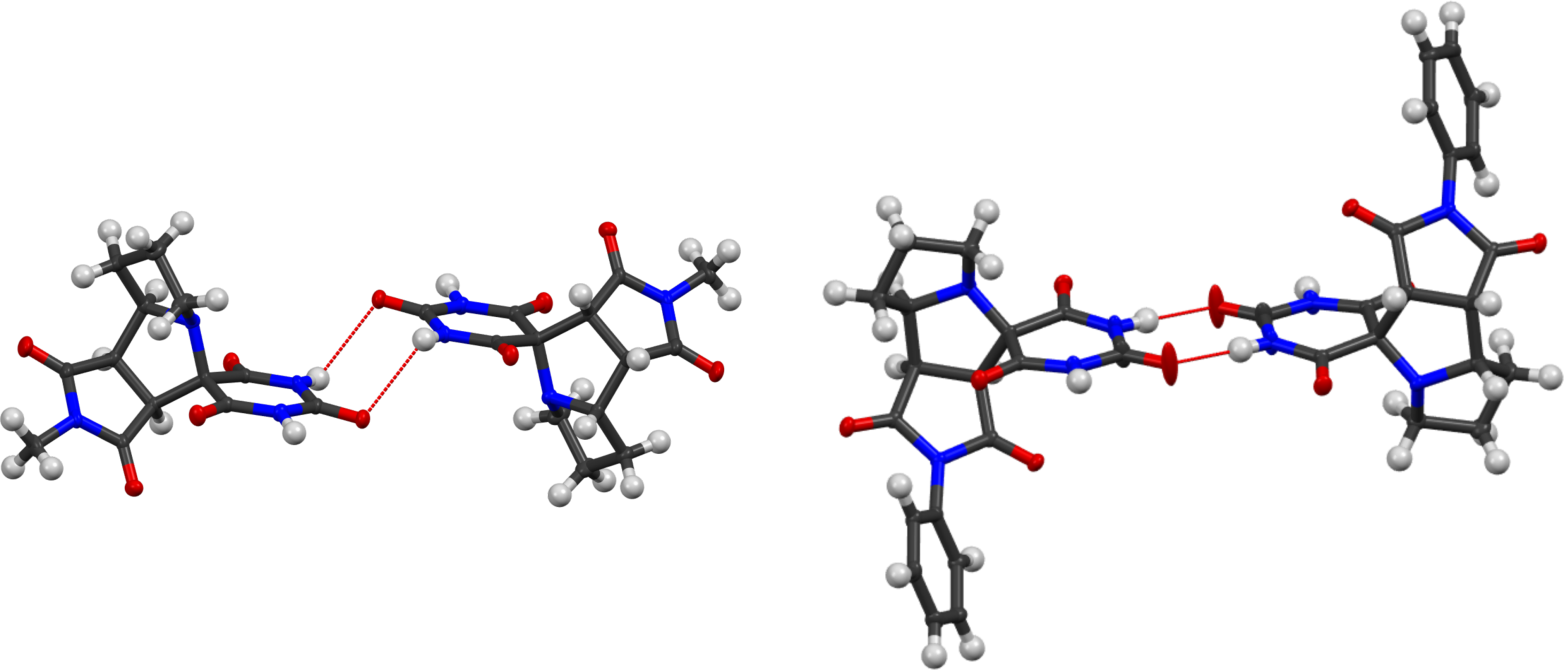
Unit cell packing of products **4b** (left) and **4c** (right).

The molecular structures of **4b** and **4c** were also confirmed by XRD (Figure 2). Both compounds crystallize as racemates, Figure 3 displaying their unit cell fragments. In **4b**, the two enantiomers are connected through weak hydrogen bonds (N–H···O 2.612 Å) between the barbiturate rings. In contrast, compound **4c** exhibits classical hydrogen bonding (N–H···O 1.878 Å) linking barbiturate moieties of paired enantiomers (see Supporting Information File 1, Tables S2 and S3).

**Figure 4:**
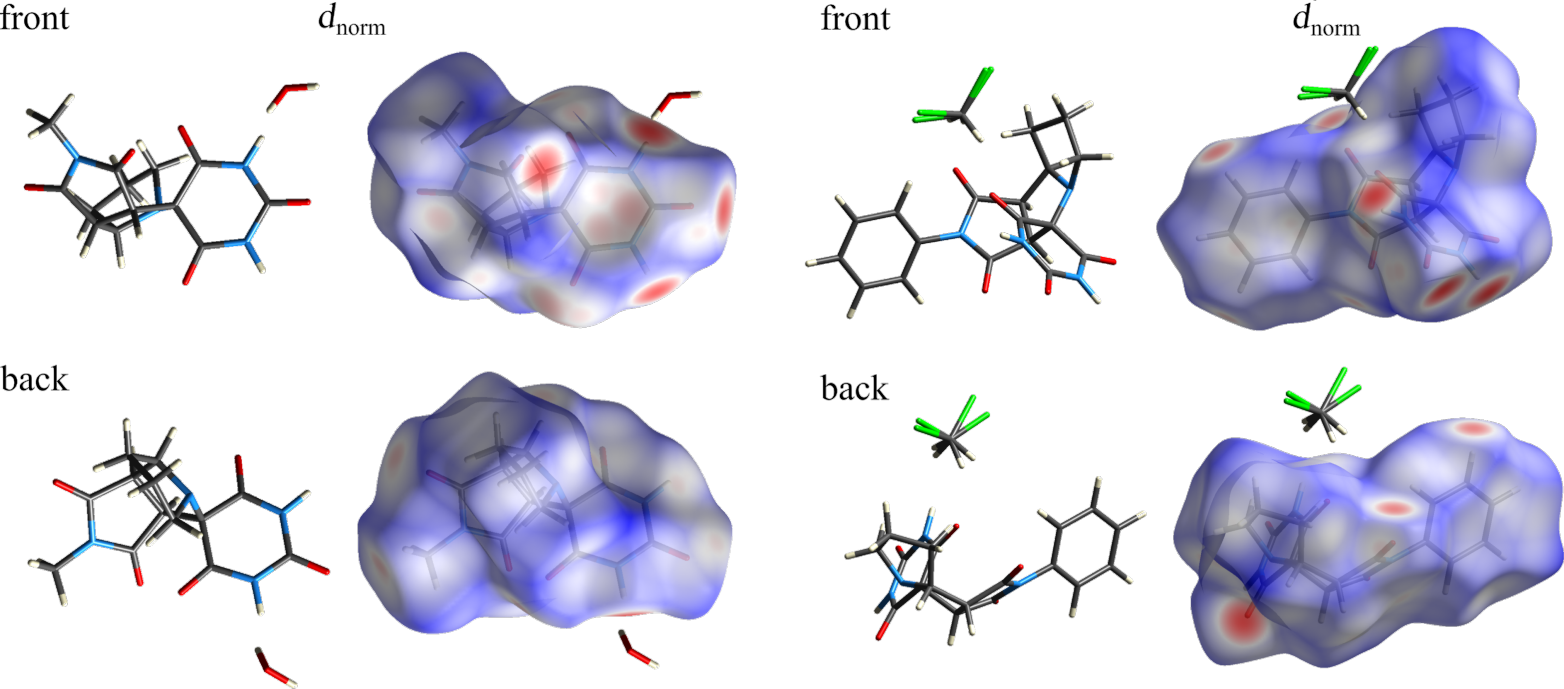
HS mapped with *d*_norm_ for compounds **4b** (left) and **4c** (right).

The Hirshfeld surface (HS) analysis provides a robust tool for visualizing and characterizing intermolecular interactions in organic crystal lattices. The calculations of the HS were performed using CrystalExplorer 21.5 [55]. On the Hirshfeld surface, color coding indicates the nature of intermolecular interactions: red highlights contacts shorter than the van der Waals radius, white represents distances equal to the van der Waals radius, and blue denotes contacts longer that the van der Waals radius. Figure 4 shows front and back views of the Hirshfeld surfaces (*d*_norm_) for compounds **4b** and **4c**.

**Figure 5:**
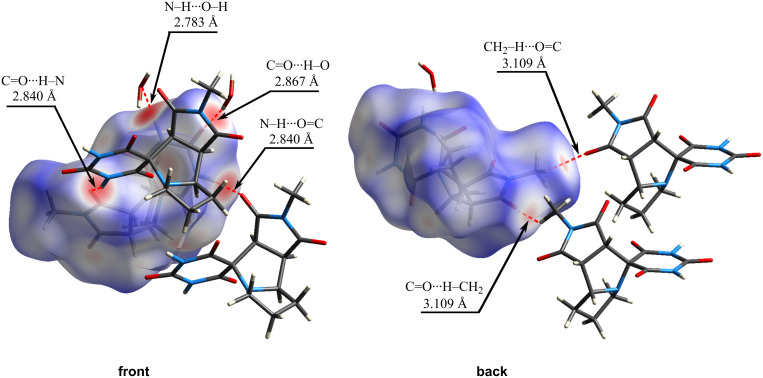
A segment of the crystal structure of compound **4b** with the HS (*d*_norm_), showing intermolecular contacts (lengths shown for D···A).

The Hirshfeld surface for compound **4b** with *d*_norm_ map plotted (ranging from −0.8499 (red) to 1.4229 (blue) a.u.) is presented in Figure 5. Analysis of the HS revealed the presence of several intermolecular contacts: the barbiturate ring forms hydrogen bonds with water molecules through C=O···H–O and N–H···O–H interactions; the amide group of the barbiturate ring is engaged in a C=O···H–N hydrogen bond with the carbonyl oxygen of the maleimide fragment; additionally, a C=O···H–CH_2_ contact is observed between the hydrogen atom of the maleimide methyl group and the carbonyl oxygen of an adjacent maleimide ring.

The crystal structure of compound **4b** exists as a solvate. The oxygen atom of a water molecule forms a hydrogen bond with the amide hydrogen of the barbiturate fragment (N–H···O–H = 2.783 Å), while one of the water hydrogens interacts with a carbonyl group of the barbiturate ring (C=O···H–O = 2.867 Å).

**Figure 6:**
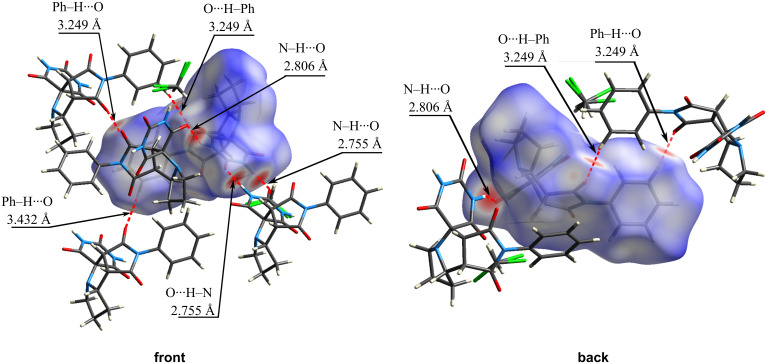
A segment of the crystal structure of compound **4c** with the HS (*d*_norm_), showing intermolecular contacts (lengths shown for D···A).

The HS for compound **4c** with *d*_norm_ map plotted (ranging from −0.6673 (red) to 1.4313 (blue) a.u.), is shown in Figure 6. The *d*_norm_ map highlights key intermolecular interactions (marked in red), including O···H–N hydrogen bonds between barbiturate rings and O···H–Ph contacts involving maleimide carbonyl oxygens and phenyl ring hydrogens.

The crystal structure of adduct **4c** includes a molecule of dichloromethane. Due to the disorder of the dichloromethane molecule, the exact distances of the corresponding intermolecular interactions cannot be precisely determined. However, the nature of these interactions can be described as follows: one of the hydrogen atoms of dichloromethane contacts with the oxygen atoms of carbonyl groups from two nearby barbiturate rings. The estimated contact distances are C=O···H ≈ 3.6 Å, which is consistent with weak hydrogen-bonding interactions.

**In silico analyses of drug-like properties.** An in silico analysis was performed to preliminarily determine whether the synthesized spiro-fused adducts have drug-like properties. The physicochemical profile was determined using the free online software SwissADME (http://www.swissadme.ch/). The molecular descriptors were calculated according to Lipinski’s rule of five. This rule was formulated by Ch.A. Lipinski based on the observation that most orally administered drugs are relatively small and moderately lipophilic molecules [56]. By this rule, orally active drugs should not violate more than one of the following criteria: MW – molecular weight: < 500 Da; *N*_HBD_ – number of hydrogen-bond donors, Log(P) – octanol/water partition coefficient: <5; N_HBA_ – number of hydrogen-bond acceptors, *N*_RotB_ – number of rotatable bonds: <10 and TPSA – topological polar surface area: <140 Å^2^. The obtained results are presented in Supporting Information File 1, Table S4.

Since antitumor drugs usually damage healthy cells along with tumor cells, while developing new substances, it is extremely important to pay attention to their ADMET properties: absorption, distribution, metabolism, excretion, and toxicity. In this paper, these were evaluated in silico using an online resource accessible via https://preadmet.webservice.bmdrc.org/. The following ADME descriptors were selected: blood-brain barrier permeability (BBB), human intestinal absorption (HIA), in vitro permeability to Caco-2 cells (Caco2), in vitro binding to plasma proteins (PPB), solubility, and inhibition of CYP2D6. The following were selected as descriptors of toxicity: carcinogenicity for rats and mice, mutagenicity according to the Ames test, and cardiotoxicity by inhibition of hERG in vitro. The results are shown in Supporting Information File 1, Table S5.

As can be seen from the table, the obtained results suggest that the compounds have a good intestinal absorption and medium permeability. However they are expected to have low plasma protein binding and permeation potential in the brain with regard to bioavailability in the CNS.

**Antiproliferative activity study.** Cancer cells are favorable in vitro models that are widely used in cancer research and drug discovery. In this study, the MTS assay was applied to evaluate the antiproliferative activity of the synthesized compounds against human cervical carcinoma (HeLa), erythroleukemia (K562), and melanoma (Sk-mel-2) cell lines. It was found that the studied spiro-fused adducts reduced the cell proliferation in a time- and concentration-dependent manner. The results of these investigations for 24 h and 72 h are presented in Supporting Information File 1, Figures S77–S79. As can be seen from the obtained results, the compounds generally have a limited antiproliferative effect against these cells in contrast to previously studied compounds that contain a cyclopropane moiety instead of a pyrrolidine one [57] and tryptanthrin, 11*H*-benzo[4,5]imidazo[1,2-*a*]indol-11-one or indolin-2-one instead of the barbituric moiety [58–60]. The most interesting effect however, was the increased Sk-mel-2 cell viability under treatment with compounds **4f** and **4g** at the lowest concentration tested (5 µg/mL).

**Actin cytoskeleton changes.** It is known that actin plays an important role in vital cellular processes, providing a number of functions such as cell migration, adhesion, and morphogenesis [61–62]. The dynamic reorganization of the actin cytoskeleton, which plays a key role in a variety of cellular functions, directly determines cell motility, and its structural organization can serve as an indicator of the metastatic potential of tumor cells [63–65].

The presence of stress fibers and filopodia-like protrusions were used to evaluate the Sk-mel-2 cells' actin cytoskeleton structure after their co-incubation with spiro-fused adducts **4f**, **4g**, **4i**, **4k**, and **4l**.

**Figure 7:**
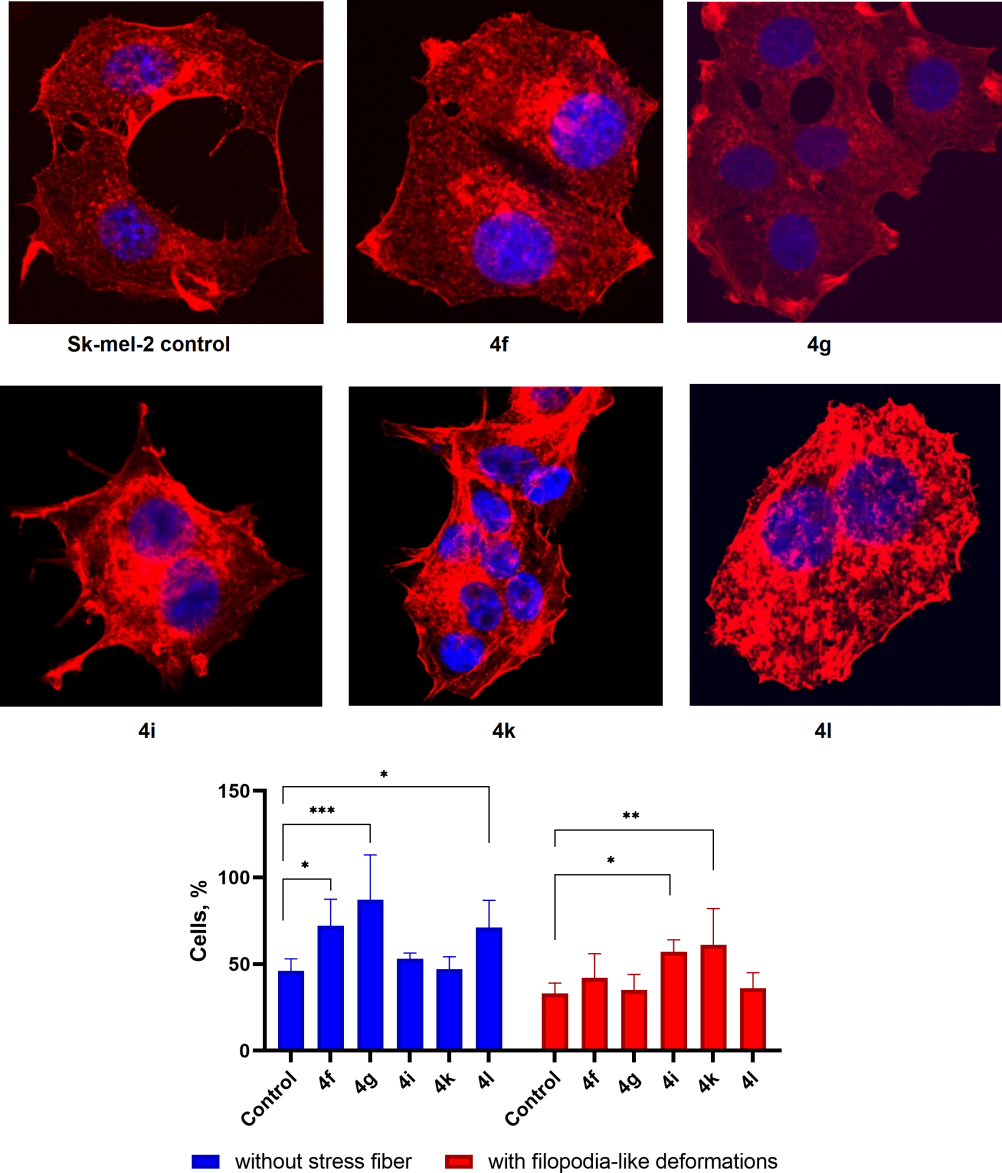
Microscopic images of treated cells and state of the actin cytoskeleton of Sk-mel-2 cells after cultivation with compounds **4f**, **4g**, **4i**, **4k**, and **4l** (10 μg/mL); *p*-value <0.05 (*), 0.01 (**), 0.001 (***).

Confocal microscopy showed that co-incubation of Sk-mel-2 cells with adducts **4f**, **4g**, **4i**, **4k**, and **4l** led to significant changes in the structure of their actin cytoskeleton resulting in stress fibers disappearance (granular actin was found in the cytoplasm of up to 87% cells) and increased number of filopodia-like deformations (up to 61% after co-incubation). Such changes in the cytoskeleton structure may indicate a change in the motor activity of cells. Although compounds **4f** and **4g** showed increased cell viability, they may have a reduced wound healing ability if applied to non-tumor cells. At the same time, nucleus fragmentation was not observed during the experiment which may indicate the absence of pro-apoptotic activity. Data on actin cytoskeleton structure as well as data demonstrating percentage of cells with filopodia-like deformations and disassembled stress fibers are combined at Figure 7.

Additionally, a scratch-test was performed to study the wound healing ability of these compounds and the results are given in Supporting Information File 1, Figure S80 and Table S6. The measured wound area after 24 h of incubation with compounds **4f**, **4g**, **4i**, **4k** and **4l** was equal to 25, 19, 31, 21, 23, and 19% for the tested compounds and control sample, respectively. As can be seen from the obtained data the wound healing ability are consistent with the cytoskeleton data. Compounds **4g** and **4l**, which led to less stress fibers (87 ± 22%, 71 ± 16% vs 46 ± 7% for **4g**, **4l** vs control, respectively) and nearly the same filopodia-like deformations (35 ± 15% and 36 ± 22% vs 33 ± 17% for **4g** and **4l** vs control, respectively) as compared to the control, result in low wound healing ability when applied to Sk-mel-2 cells. At the same time, compound **4i** which led to slightly less stress fibers (53 ± 3% vs 46 ± 7% for **4i** vs control, respectively) and more filopodia-like deformations (57 ± 3% vs 33 ± 17% for **4i** vs control, respectively) as compared to the control, result in increased wound healing ability in treated Sk-mel-2 cells.

**Molecular docking.** To confirm the results obtained, docking simulations were performed for the possible interaction of spiro-adducts with actin, as the most widespread and highly conserved cellular protein. Since the initial determination of the G-actin crystal structure in complex with DNase I, many actin structures have been registered. Taking into account that the conformation of the actin monomer in the described structures is basically the same, the structure of non-muscle β-actin (8DNH, 2.99 Å) obtained by cryo-electron microscopy was used for this study. Docking was performed to both known clefts (hydrophobic or target-binding one and nucleotide, DNaze I-binding).

**Figure 8:**
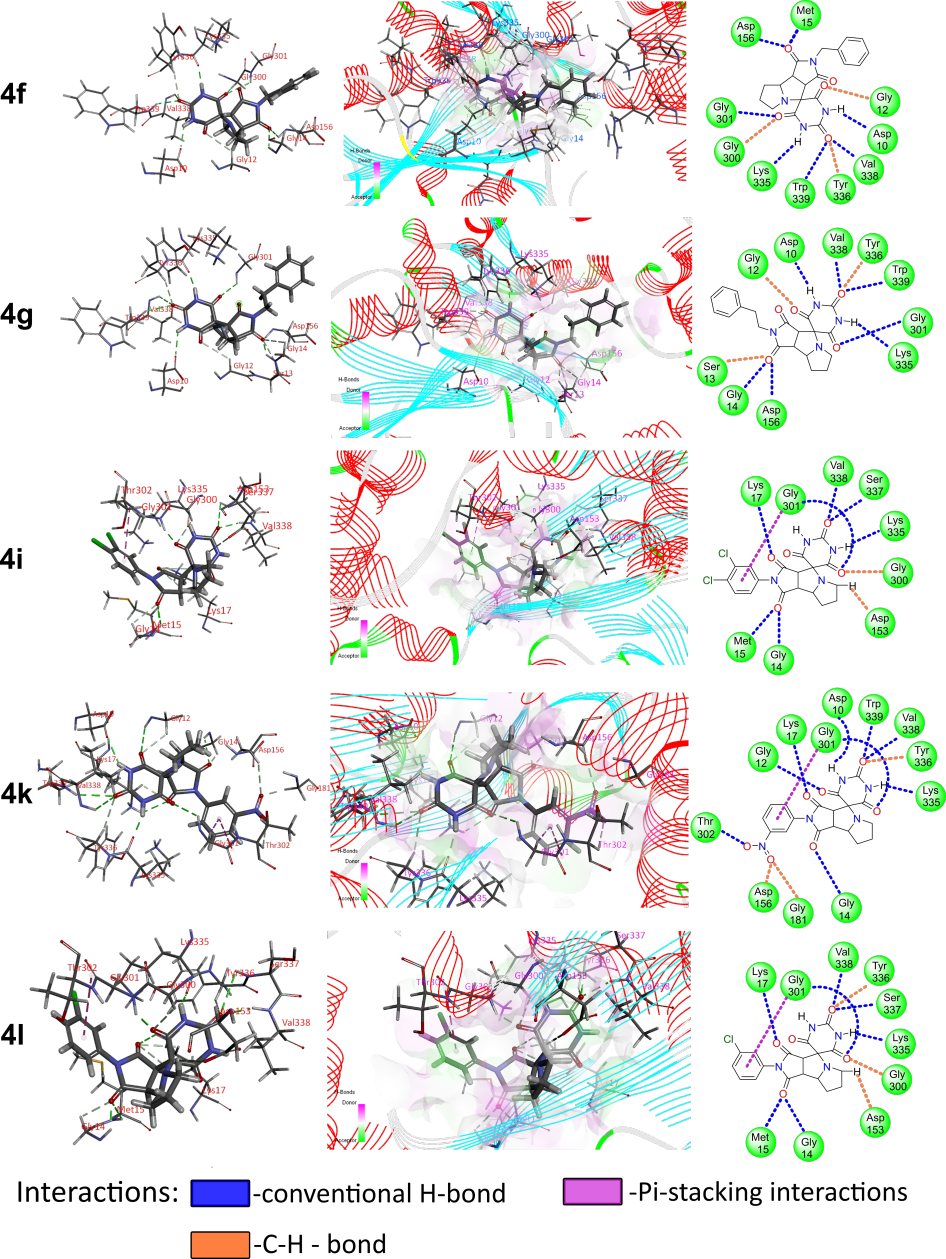
Docked view of compounds **4f**, **4g**, **4i**, **4k**, and **4l** with the target protein (PDB ID: 8DNH).

The structure of the 8DNH protein was retrieved from the protein data bank and the Molegro Virtual Docker 6.0 software was used to prepare it for the docking study [66–67]. The pose organizer and the ligand energy inspector tool were used to examine the docking results. The latter were then tabulated and the docked view was retrieved. Figure 8 and Table S7 in Supporting Information File 1 show the docking results. The analysis of the data obtained, shows that the affinity for the hydrophobic target-binding cleft was always lower in comparison to nucleotide cleft (Rerank Score was found to be from −77 to −108 and from −93 to −125 arbitrary units for both clefts correspondingly, data are collected in Table S7 in Supporting Information File 1). The predicted binding models also revealed that the adducts, while being fitted within the cleft, usually are located in such way that the barbituric moiety is directed to Trp 339, while the aryl substituent of the imide moiety is directed to Lys 212. This result agrees with the data on changes in the actin cytoskeleton and can be caused by a balance disturbance in the processes of actin polymerization and depolymerization that continuously occur in cells.

## Conclusion

We have developed a novel one-pot three-component method for the synthesis of pyrrolizidine-containing spirobarbiturates via 1,3-dipolar cycloaddition reactions. The study revealed that maleimide derivatives with -H, -Me, -Ph, *p*-Tol, or 4-NO_2_С_6_H_4_ substituents exclusively form *endo*-adducts with yields ranging from 22% to 52%, while the use of maleimides bearing -Bn, -CH_2_CH_2_Ph, or halogenated aromatic substituents led to a significant decrease in diastereoselectivity, resulting in mixtures of *endo-* and *exo-*isomers with yields of 25–70%. Notably, *meta*-halogen substitution in aromatic maleimides was found to preferentially direct the cycloaddition toward the formation of *exo*-isomers. The structures of all obtained diastereomers were thoroughly characterized by ^1^H and ^13^C NMR spectroscopy. Single crystal XRD analysis of two representative compounds allowed unambiguous determination of the absolute configuration of the spirobarbiturate adducts. Additionally, a detailed analysis of intermolecular interactions in the crystal structure was performed using Hirshfeld surface calculations, providing valuable insights into the packing arrangements of these compounds. The results of antiproliferative activity study showed that generally spiro-adducts have limited effect on the tested cancer cells, while they caused significant changes of Sk-mel-2 cells’ actin cytoskeleton structure leading to the disappearance of stress fibers (granular actin was distributed diffusely in the cytoplasm of treated cells in up to 87%) and changes in the number of filopodia-like deformations (increased up to 61% after cultivation).

## Supporting Information

File 1 General information, experimental procedures, characterization data, X-ray data and biological activity data.

## Data Availability

All data that supports the findings of this study is available in the published article and/or the supporting information of this article.
