# Supplementary material for: Spirobarbiturates with a pyrrolizidine moiety: synthesis, structure and biological evaluation
Source: Beilstein J Org Chem. 2026 Feb 17;22:274–88. doi: 10.3762/bjoc.22.20 (PMC12927484; doi:10.3762/bjoc.22.20)
Supplement: File 1 — General information, experimental procedures, characterization data, X-ray data and biological activity data. [file Beilstein_J_Org_Chem-22-274-s001.pdf]

BEILSTEIN JOURNAL OF ORGANIC CHEMISTRY

Supporting Information

for

Spirobarbiturates with a pyrrolizidine moiety: synthesis, structure and biological evaluation

Arthur A. Puzyrkov, Andrew S. Drachuk, Ekaterina A. Popova, Alexander V. Stepakov and Vitali M. Boitsov

Beilstein J. Org. Chem. **2026,** *22,* 274–288. doi:10.3762/bjoc.22.20

General information, experimental procedures, characterization data, X-ray data and biological activity data

License and Terms: This is a supporting information file under the terms of the Creative Commons Attribution License (https://creativecommons.org/licenses/by/4.0). Please note that the reuse, redistribution and reproduction in particular requires that the author(s) and source are credited and that individual graphics may be subject to special legal provisions.
The license is subject to the *Beilstein Journal of Organic Chemistry* terms and conditions: (https://www.beilstein-journals.org/bjoc/terms)

Table of contents

Materials and methods S2

General procedure A for the synthesis of 2’-substituted hexahydro-1'*H*,2*H*-spiro[pyrimidine-5,4'-pyrrolo[3,4-*a*]pyrrolizine]-1',2,3',4,6(1*H*,2'*H*,3*H*)-pentaones **4a**–**p** S2

The ^{1}H NMR, ^{13}C NMR, ^{19}F NMR, ^{13}C DEPT NMR, COSY ^{1}H,^{1}H NMR, HSQC ^{1}H,^{13}C NMR spectra of compounds **4a**–**p** S12

X-Ray crystallographic data for compounds **4b** (CCDC 2391172) and **4c** (CCDC 2391171) S55

Biological activity study S57

Materials and methods

All reagents used were purchased from commercial suppliers and were used without further purification. The progress of the reaction was monitored by TLC on Silufol UV-254 plates using UV light. Preparative thin-layer chromatography (PTLC) was performed on glass plates using high-purity grade (7749) Silica gel, with gypsum binder and fluorescent indicator (particle size ≤55 µm). All solvents were purified according to standard techniques before use. Diethyl ether was distilled over sodium metal. 1,4-Dioxane was refluxed in the presence of sodium metal for 24 hours, filtered off and distilled over sodium metal. All the chemicals were purchased commercially, and used without further purification. NMR spectra were recorded with a Bruker Avance 400 spectrometer (400.13 MHz for ^{1}H, 100.61 MHz for ^{13}C, 376.50 MHz for ^{19}F). Chemical shifts are reported in ppm relative to residual DMSO-d_6 (^{1}H, 2.50 ppm), DMSO-d_6 (^{13}C, 39.52 ppm) as internal standards. All ^{13}C NMR spectra were proton-decoupled. Mass spectra were recorded with a HRMS-ESI-QTOF mass-analyzer, electrospray ionization, positive mode. Single-crystal X-ray diffraction experiments for compounds **4b** and **4c** were carried out with an Agilent Technologies Xcalibur diffractometer with monochromated Mo Kα radiation. Melting points were measured on a Boetius Kofler instrument.

General procedure A for the synthesis of 2′-substituted hexahydro-1'*H*,2*H*-spiro[pyrimidine-5,4'-pyrrolo[3,4-*a*]pyrrolizine]-1',2,3',4,6(1*H*,2'*H*,3*H*)-pentaones 4a–p

Alloxan (**1**, 0.2 mmol), ʟ-proline (**2**, 0.4 mmol) and the corresponding maleimide **3a**–**p** (0.2 mmol) were placed in a tube. The resulting mixture was poured into 2–5 mL of dried 1,4-dioxane. The tube was purged with dry argon or nitrogen and tightly closed. The reaction mixture was stirred under heating at 100 °C. After the reaction (about 6 to 16 h), controlled by TLC, the reaction mixture was filtered off and evaporated under reduced pressure. Further purification of the product was carried out by PTLC, preparative column chromatography or crystallization.

(*rac*)-(3a′*S*,8a′*R*,8b′*R*)-Hexahydro-1'*H*,2*H*-spiro[pyrimidine-5,4'-pyrrolo[3,4-*a*]pyrrolizine]-1',2,3',4,6(1*H*,2'*H*,3*H*)-pentaone (4a)

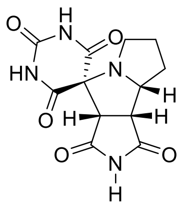

4a 24%
only endo

The synthesis was carried out according to procedure **A**. Alloxan (**1**, 0.165 g, 1.03 mmol), ʟ-proline (**2**, 0.237 g, 2.06 mmol) and maleimide **3a** (0.100 g, 1.03 mmol) were mixed with 5 mL of dried 1,4-dioxane and heated under stirring in a closed tube at 100 °C for 6 hours under argon atmosphere. The reaction mixture was filtered off, then filtrate was evaporated under reduced pressure and diluted with 1–2 mL of methanol. The resulting solution was put away in the refrigerator overnight. This resulted in the precipitation of pale pink crystals of **4a**. The crystals were filtered and dried. Yield: 24% (72 mg).

M.p.: 130.5-132.0°C; ^{1}H NMR (400 MHz, DMSO-d_6, δ, ppm): 1.68-1.75 (m, 2H), 1.77-1.88 (m, 1H), 1.91-2.02 (m, 1H), 2.55-2.64 (m, 2H), 3.52 (dd, 1H, J = 8.6 Hz, *J* = 8.8 Hz), 3.92 (d, 1H, *J* = 8.8 Hz), 3.94 (d, 1H, *J* = 7.9 Hz), 11.30 (bs, 1H), 11.57 (bs, 2H); ^{13}C NMR (101 MHz, DMSO-d_6, δ_C, ppm): 25.4, 27.3, 44.0, 44.9, 60.5, 66.8, 67.2, 69.5, 150.0, 169.3, 171.5, 176.8, 177.9; HRMS (ESI): m/z [M - H]$^{+}$ calculated for $C_{12}H_{12}N_4O_5$: 291.0724, found: 291.0738

(*rac*)-(3a'*S*,8a'*R*,8b'*R*)-2'-Methylhexahydro-1'*H*,2*H*-spiro[pyrimidine-5,4'-pyrrolo[3,4-*a*]pyrrolizine]-1',2,3',4,6(1*H*,2'*H*,3*H*)-pentaone (4b)

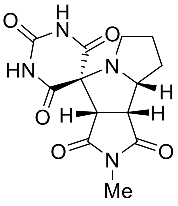

4b 22%
only endo

The synthesis was carried out according to procedure **A**. Alloxan (**1**, 0.144 g, 0.9 mmol), ʟ-proline (**2**, 0.207 g, 1.8 mmol) and *N*-methylmaleimide (**3b**, 0.100 g, 0.9 mmol) were mixed with 9 mL of dried 1,4-dioxane and heated under stirring in a closed tube at 100 °C for 6 hours under nitrogen atmosphere. The reaction mixture was filtered off and filtrate was evaporated under reduced pressure. The residue was subjected to silica gel and purified by preparative column chromatography using a mixture of CH_2Cl_2:MeOH (30:1) as eluent. White solid. Yield: 22% (60 mg).

M.p.: 201.6-202.6 °C (decomposition); ^{1}H NMR (400 MHz, DMSO-d_6, δ, ppm): 1.65-1.78 (m, 2H), 1.77-1.90 (m, 1H), 1.90-2.02 (m, 1H), 2.54-2.63 (m, 2H), 2.79 (s, 3H), 3.57 (dd, 1H, *J* = 8.4 Hz, *J* = 8.3 Hz), 3.93-3.98 (m, 1H), 4.00 (bd, 1H, *J* = 8.4 Hz), 11.61 (bs, 2H); ^{13}C NMR (101 MHz, DMSO-d_6, δ_C, ppm): 25.2, 25.3, 27.2, 44.1, 45.8, 58.9, 67.2, 69.4, 150.0, 169.4, 171.2, 175.5, 176.4; HRMS (ESI): m/z $[M + H]^+$ calculated for $C_{13}H_{15}N_4O_5$: 307.1037; found: 307.1041.

(*rac*)-(3a'*S*,8a'*R*,8b'*R*)-2'-Phenylhexahydro-1'*H*,2*H*-spiro[pyrimidine-5,4'-pyrrolo[3,4-*a*]pyrrolizine]-1',2,3',4,6(1*H*,2'*H*,3*H*)-pentaone (4c)

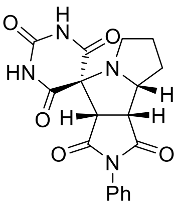

4c 44%
only endo

The synthesis was carried out according to procedure **A**. Alloxan (**1**, 0.092 g, 0.6 mmol), ʟ-proline (**2**, 0.133 g, 1.2 mmol) and *N*-phenylmaleimide (**3c**, 0.100 g, 0.6 mmol) were mixed with 5 mL of dried 1,4-dioxane and heated under stirring in a closed tube at 100 °C for 10 hours under nitrogen atmosphere. The reaction mixture was filtered off and filtrate was evaporated under reduced pressure. The residue was subjected to silica gel and purified by preparative column chromatography using a mixture of CH_2Cl_2:MeOH (30:1) as eluent. White solid. Yield: 44% (94 mg).

M.p.: 260.9-261.5 °C (decomposition); ^{1}H NMR (400 MHz, DMSO-d_6, δ, ppm): 1.70-2.03 (m, 4H), 2.56-2.75 (m, 2H), 3.80 (dd, *J* = 8.7 Hz, *J* = 8.6 Hz, 1H), 4.04 (ddd, *J* = 6.2 Hz, *J* = 8.7 Hz, *J* = 9.0 Hz, 1H), 4.20 (d, *J* = 9.0 Hz, 1H), 7.26 (bd, *J* = 8.2 Hz, 2H), 7.40-7.47 (m, 1H), 7.48-7.56 (m, 2H), 11.74 (bs, 2H). ^{13}C NMR (101 MHz, DMSO-d_6, δ_C, ppm): 25.3, 27.2, 45.0, 46.4, 58.3, 67.7, 70.3, 127.3, 128.9, 129.4, 132.8, 150.0, 169.8, 171.0, 174.8, 175.5; HRMS (ESI): m/z $[M + H]^+$ calculated for $C_{18}H_{17}N_4O_5$: 369.1193; found: 369.1188.

(*rac*)-(3a'*S*,8a'*R*,8b'*R*)-2'-(*p*-Tolyl)hexahydro-1'*H*,2*H*-spiro[pyrimidine-5,4'-pyrrolo[3,3-*a*]pyrrolizine]-1',2,3',4,6(1*H*,2'*H*,3*H*)-pentaone (4d)

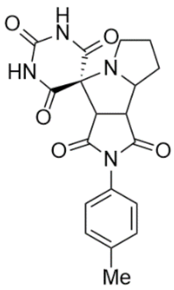

4d 52%
only endo

The synthesis was carried out according to procedure **A**. Alloxan (**1**, 0.085 g, 0.5 mmol), ʟ-proline (**2**, 0.123 g, 1.1 mmol) and *N*-(*p*-tolyl)maleimide (**3d**, 0.100 g, 0.5 mmol) were mixed with 5 mL of dried 1,4-dioxane and heated under stirring in a closed tube at 100 °C for 10 hours under nitrogen atmosphere. The reaction mixture was filtered off, then filtrate was evaporated under reduced pressure. The residue was subjected to silica gel and purified by preparative column chromatography using a mixture of CH_2Cl_2:MeOH (30:1) as eluent. White solid. Yield: 52% (107 mg).

M.p.: 190.8-191.8 °C (decomposition); ^{1}H NMR (400 MHz, DMSO-d_6, δ, ppm): 1.69-1.92 (m, 3H), 1.93-2.05 (m, 1H), 2.56-2.72 (m, 2H), 3.78 (dd, 1H, *J* = 8.9 Hz, *J* = 8.7 Hz), 3.97-4.08 (m, 1H), 4.14 (d, 1H, *J* = 8.9 Hz), 7.12 (d, 1H, *J* = 8.2 Hz), 7.30 (d, 1H, *J* = 8.2 Hz), 11.7 (bs, 2H); ^{13}C NMR (101 MHz, DMSO-d_6, δ_C, ppm): 25.4, 21.2, 27.2, 44.9, 46.3, 58.3, 67.7, 70.3, 127.1, 129.8,

130.2, 138.4, 150.1, 169.9, 171.2, 174.9, 175.6; HRMS (ESI): m/z [M - H]$^+$ calculated for $C_{19}H_{18}N_4O_5$: 381.1204; found: 381.1206.

(*rac*)-(3a'*S*,8a'*R*,8b'*R*)-2'-(4-Nitrophenyl)hexahydro-1'*H*,2*H*-spiro[pyrimidine-5,4'-pyrrolo[3,3-*a*]pyrrolizine]-1',2,3',4,6(1*H*,2'*H*,3*H*)-pentaone (4e)

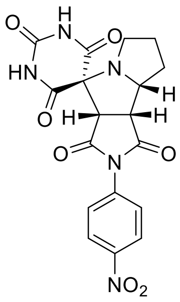

4e 37%
only endo

The synthesis was carried out according to procedure **A**. Alloxan (**1**, 0.072 g, 0.5 mmol), L-proline (**2**, 0.106 g, 0.9 mmol) and *N*-(4-nitrophenyl)maleimide (**3e**, 0.100 g, 0.5 mmol) were mixed with 5 mL of dried 1,4-dioxane and heated under stirring in a closed tube at 100 °C for 6 hours under argon atmosphere. The reaction mixture was filtered off, then filtrate was evaporated under reduced pressure and diluted with 1–2 mL of methanol. This resulted in the precipitation of a white solid of **4e**. The precipitate was filtered on a Schott filter and dried. The white powdery solid obtained was insoluble in MeOH, CH_2Cl_2, $CHCl_3$. Yield: 37% (68 mg).

M.p.: slow decomposition under 130 °C; ^{1}H NMR (400 MHz, DMSO-d_6, δ, ppm): 1.68-2.07 (m, 4H), 2.55-2.73 (m, 2H), 3.83 (dd, *J* = 8.7 Hz, *J* = 8.5 Hz, 1H), 4.03 (ddd, *J* = 7.3 Hz, *J* = 8.0 Hz, *J* = 8.5 Hz, 1H), 4.20 (d, *J* = 8.7 Hz, 1H), 7.58 (d, *J* = 8.8 Hz, 2H), 8.41 (d, *J* = 8.8 Hz, 2H), 11.74 (bs, 2H); ^{13}C NMR (101 MHz, DMSO-d_6, δ_C, ppm): 24.9, 27.0, 44.9, 46.7, 57.9, 67.6, 70.2, 124.9, 128.2, 138.7, 147.2, 150.0 (NH-C(O)-NH), 169.7, 170.6, 174.4, 175.0; HRMS (ESI): m/z [M - H]$^+$ calculated for $C_{18}H_{15}N_5O_7$·MeOH: 444.1150; found: 444.1179.

(*rac*)-(*endo,exo*)-2'-Benzylhexahydro-1'*H*,2*H*-spiro[pyrimidine-5,4'-pyrrolo[3,4-*a*]pyrrolizine]-1',2,3',4,6(1*H*,2'*H*,3*H*)-pentaone (4f)

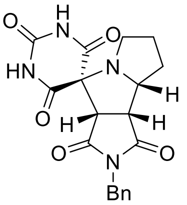

4f 53%
endo:exo 1:0.3

The synthesis was carried out according to procedure **A**. Alloxan (**1**, 0.085 g, 0.5 mmol), L-proline (**2**, 0.123 g, 1.1 mmol) and *N*-benzylmaleimide (**3f**, 0.100 g, 0.5 mmol) were mixed with 5 mL of dried 1,4-dioxane and heated under stirring in a closed tube at 100 °C for 16 hours under argon atmosphere. The reaction mixture was filtered off and filtrate was evaporated under reduced pressure. The residue was diluted with 1–2 mL of methanol and subjected to a plate for PTLC, using a mixture of CH_2Cl_2:MeOH (25:1) as eluent. Compound **4f** obtained after chromatography was a glassy mass. After addition of Et_2O a white solid of **4f** was formed. Yield: 53% (109 mg).

Main isomer (33:10): ^{1}H NMR (400 MHz, DMSO-d_6, δ, ppm): 1.59-1.99 (m, 4H), 2.53-2.73 (m, 2H), 3.26-3.37 (bs, 2H), 3.63 (d, *J* = 8.4 Hz), 3.95-4.05 (m, 1H), 4.10 (d, *J* = 8.6 Hz), 4.44 (d, *J* = 15.1 Hz), 4.61 (d, *J* = 15.1 Hz), 7.06-7.54 (m, 5H), 11.65 (bs, 2H); ^{13}C NMR (101 MHz, DMSO-d_6, δ_C, ppm): 25.1, 27.0, 42.3, 44.2, 46.0, 58.6, 67.1, 69.6, 127.7, 128.0, 128.8, 136.2, 150.0, 168.9, 171.1, 175.3, 176.2; HRMS (ESI): m/z [M + Na]$^+$ calculated for $C_{19}H_{18}N_4O_5Na$: 405.1169; found: 405.1180.

(*rac*)-(*endo,exo*)-2'-Phenethylhexahydro-1'*H*,2*H*-spiro[pyrimidine-5,4'-pyrrolo[3,4-*a*]pyrrolizine]-1',2,3',4,6(1*H*,2'*H*,3*H*)-pentaone (4g)

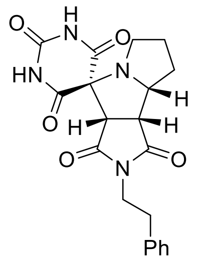

4g 27%
endo:exo 1:0.25

The synthesis was carried out according to procedure **A**. Alloxan (**1**, 0.073 g, 0.5 mmol), L-proline (**2**, 0.105 g, 0.9 mmol) and *N*-phenetylmaleimide (**3g**, 0.100 g, 0.5 mmol) were mixed with 3 mL of dried 1,4-dioxane and heated under stirring in a closed tube at 100 °C for 16 hours under argon atmosphere. The reaction mixture was filtered off and filtrate was evaporated under reduced pressure. The residue was diluted with 1–2 mL of methanol and subjected to a plate for PTLC, using a mixture of CH_2Cl_2:MeOH (20:1) as eluent. Compound **4g** obtained after chromatography was a glassy mass. After addition of Et_2O a white solid of **4g** was formed. Yield: 27% (48 mg).

^{1}H NMR (400 MHz, DMSO-d_6, δ, ppm): There are the signals of Et_2O, as cocrystallysed solvent, in spectra.

endo-isomer: 1.54-1.74 (m, 2H), 1.75-1.88 (m, 1H), 1.88-2.00 (m, 1H), 2.49-2.59 (m, 1H), 2.60-2.69 (m, 1H), 2.69-2.82 (m, 2H), 3.46-3.58 (m, 3H), 3.91-4.03 (m, 2H), 7.15-7.35 (m, 5H), 11.61 (bs, 2H);

exo-isomer: 1.78-2.13 (m, 4H), 2.49-2.59 (m, 1H), 2.60-2.69 (m, 1H), 2.69-2.82 (m, 2H), 3.44-3.58 (m, 3H), 3.70-3.76 (m, 1H), 4.37 (d, J = 10.5 Hz, 1H), 7.15-7.35 (m, 5H), 11.61 (bs, 2H).

^{13}C NMR (101 MHz, DMSO-d_6, δ_C, ppm): 25.1 (endo), 25.4 (exo), 27.1 (endo), 29.6 (exo), 33.1 (exo), 33.2 (endo), 39.4 (exo), 40.1 (endo), 44.0 (endo), 45.8 (endo), 48.8 (exo), 50.4 (exo), 53.9 (exo), 58.4 (endo), 66.8 (exo), 67.1 (endo), 69.4 (endo), 73.3 (exo), 126.8 (endo), 126.9 (exo), 128.8 (endo), 129.0 (exo), 129.2 (endo), 138.76 (endo), 138.79 (exo), 150.3, 159.5, 161.4, 168.0, 169.3, 171.3, 175.4, 175.8, 176.3, 177.7;

HRMS (ESI): m/z $[M + H]^+$ calculated for $C_{20}H_{20}N_4O_5$: 397.1506; found: 397.1508.

(*rac*)-(*endo,exo*)-2'-(4-Chlorophenyl)hexahydro-1'*H*,2*H*-spiro[pyrimidine-5,4'-pyrrolo[3,3-*a*]pyrrolizine]-1',2,3',4,6(1*H*,2'*H*,3*H*)-pentaone (4h)

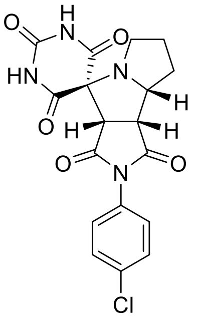

4h 34%
endo:exo 1:0.9

The synthesis was carried out according to procedure **A**. Alloxan (**1**, 0.077 g, 0.5 mmol), L-proline (**2**, 0.111 g, 1.0 mmol) and *N*-(4-chlorophenyl)maleimide (**3h**, 0.100 g, 0.5 mmol) were mixed with 5 mL of dried 1,4-dioxane and heated under stirring in a closed tube at 100 °C for 6 hours under argon atmosphere. The reaction mixture was filtered off and filtrate was evaporated under reduced pressure. The residue was diluted with 1–2 mL of methanol and subjected to a plate for PTLC, using a mixture of CH_2Cl_2:MeOH (30:1) as eluent. Compound **4h** obtained after chromatography was a glassy mass. After addition of Et_2O a white solid of **4h** was formed. Yield: 34% (53 mg).

^{1}H NMR (400 MHz, DMSO-d_6, δ, ppm): There are the signals of Et_2O, as cocrystallysed solvent, in spectra.

exo-isomer: 1.64-1.98 (m, 3H), 2.08-2.21 (m, 1H), 2.55-2.78 (m, 2H), 3.68 (dd, *J* = 10.6 Hz, *J* = 6.9 Hz, 1H), 3.82-3.88 (m, 1H), 4.53 (d, *J* = 10.6 Hz, 1H), 7.20-7.35 (m, 2H), 7.53-7.67 (m, 2H), 11.81 (bs, 2H);

endo-isomer: 1.64-1.98 (m, 4H), 2.55-2.78 (m, 2H), 3.76-3.83 (m, 1H), 3.96-4.08 (m, 1H), 4.16 (d, *J* = 8.9 Hz, 1H), 7.20-7.35 (m, 2H), 7.53-7.67 (m, 2H), 11.81 (bs, 2H).

^{13}C NMR (101 MHz, DMSO-d_6, δ_C, ppm): 25.2, 25.4, 27.1, 29.8, 44.9, 46.5, 49.0, 50.4, 54.2, 58.2, 67.2, 67.7), 70.2, 73.9, 129.0, 129.1, 129.5, 129.6, 131.6, 131.7, 133.4, 133.4, 149.9, 150.0, 167.0, 169.1, 169.7, 170.9, 174.6, 175.3, 175.4, 177.0;

HRMS (ESI): m/z [M - H]$^+$ calculated for $C_{18}H_{15}ClN_4O_5$: 401.0658; found: 401.0664.

(*rac*)-(*endo,exo*)-2'-(3,4-Dichlorophenyl)hexahydro-1'*H*,2*H*-spiro[pyrimidine-5,4'-pyrrolo[3,3-*a*]pyrrolizine]-1',2,3',4,6(1*H*,2'*H*,3*H*)-pentaone (4i)

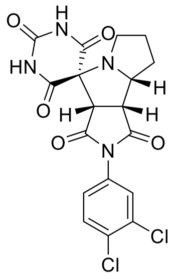

4i 50%
endo:exo 1:0.77

The synthesis was carried out according to procedure **A**. Alloxan (**1**, 0.066 g, 0.4 mmol), L-proline (**2**, 0.095 g, 0.8 mmol) and *N*-(3,4-dichlorophenyl)maleimide (**3i**, 0.100 g, 0.4 mmol) were mixed with 5 mL of dried 1,4-dioxane and heated under stirring in a closed tube at 100 °C for 16 hours under argon atmosphere. The reaction mixture was filtered off and filtrate was evaporated under reduced pressure. The residue was diluted with 1–2 mL of methanol and subjected to a plate for PTLC, using a mixture of CH_2Cl_2:MeOH (20:1) as eluent. Compound **4i** obtained after chromatography was a glassy mass, after addition of a small amount of EtOH/Et_2O mixture a white solid **4i** was precipitated. Yield: 50% (90 mg).

There are the signals of EtOH, as cocrystallysed solvent, in spectra

^1H NMR (400 MHz, DMSO-d_6, δ, ppm):

endo-isomer: 1.64-2.04 (m, 4H), 2.55-2.79 (m, 2H), 3.79 (dd, *J* = 8.4 Hz, *J* = 8.7 Hz, 1H), 3.97-4.07 (m, 1H), 4.17 (d, *J* = 8.7 Hz, 1H), 7.31 (dd, *J* = 2.2 Hz, *J* = 8.6 Hz, 1H), 7.51 (d, *J* = 2.2 Hz, 1H), 7.84 (d, *J* = 8.6 Hz, 1H), 11.81 (bs, 2H);

exo-isomer: 1.85-2.04 (m, 3H), 2.07-2.22 (m, 1H), 2.55-2.79 (m, 2H), 3.71 (dd, *J* = 10.8 Hz, *J* = 6.9 Hz, 1H), 3.82-3.89 (m, 1H), 4.54 (d, *J* = 10.8 Hz, 1H), 7.29 (dd, *J* = 2.2 Hz, *J* = 8.6 Hz, 1H), 7.50 (d, *J* = 2.2 Hz, 1H), 7.83 (d, *J* = 8.6 Hz, 1H), 11.81 (bs, 2H).

^{13}C NMR (101 MHz, DMSO-d_6, δ_C, ppm): 24.9 (endo), 25.4 (exo), 27.1 (endo), 29.8 (exo), 44.8 (endo), 46.6 (endo), 49.1 (exo), 54.2 (exo), 50.3 (exo), 58.0 (endo), 67.2 (exo), 67.6 (endo), 70.2 (endo), 74.1 (exo), 127.6 (endo), 127.7 (exo), 128.9 (endo), 129.0 (exo), 131.6 (exo), 131.62 (2C), 131.64 (endo), 131.71 (endo), 131.73 (exo), 132.5 (endo), 132.7 (exo), 149.94 (endo), 149.97 (exo), 166.9, 169.3, 169.8, 170.6, 174.4, 175.0, 175.2, 176.7

HRMS (ESI): m/z [M + H]$^+$ calculated for $C_{18}H_{14}Cl_2N_4O_5$: 437.0416 [35Cl], 439.0390 [37Cl]; found: 437.0414 [^{35}Cl], 439.0386 [^{37}Cl].

(*rac*)-(*endo,exo*)-2'-(4-Chloro-3-fluorophenyl)hexahydro-1'*H*,2*H*-spiro[pyrimidine-5,4'-pyrrolo[3,3-*a*]pyrrolizine]-1',2,3',4,6(1*H*,2'*H*,3*H*)-pentaone (4j)

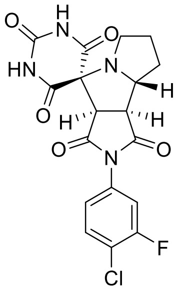

4j 70%
endo:exo 1:2

The synthesis was carried out according to procedure **A**. Alloxan (**1**, 0.071 g, 0.4 mmol), L-proline (**2**, 0.102 g, 0.9 mmol) and *N*-(4-chloro-3-fluorophenyl)maleimide (**3j**, 0.100 g, 0.4 mmol) were mixed with 5 mL of dried 1,4-dioxane and heated under stirring in a closed tube at 100 °C for 16 hours under argon atmosphere. The reaction mixture was filtered off and filtrate was evaporated under reduced pressure. The residue was diluted with 1–2 mL of methanol and subjected to a plate for PTLC, using a mixture of CH_2Cl_2:MeOH (20:1) as eluent. Compound **4j** obtained after chromatography was a glassy mass, after addition of a small amount of Et_2O a white solid **4j** was precipitated. Yield: 70% (130 mg).

^{1}H NMR (400 MHz, DMSO-d_6, δ, ppm): There are the signals of Et_2O, as cocrystallysed solvent, in spectra.

endo-isomer: 1.62-2.05 (m, 3H), 2.06-2.22 (m, 1H), 2.55-2.75 (m, 2H), 3.70 (dd, J = 10.6 Hz, J = 6.8 Hz, 1H), 3.81-3.89 (m, 1H), 4.54 (d, J = 10.6 Hz, 1H), 7.10-7.24 (m, 1H), 7.25-7.37 (m, 1H),7.72-7.85 (m, 1H), 11.86 (bs, 2H);

exo-isomer: 1.62-2.05 (m, 4H), 2.55-2.78 (m, 2H), 3.76-3.82 (m, 1H), 3.98-4.06 (m, 1H), 4.17 (d, J = 8.9 Hz, 1H), 7.10-7.24 (m, 1H), 7.25-7.37 (m, 1H),7.72-7.85 (m, 1H), 11.86 (bs, 2H).

^{13}C NMR (101 MHz, DMSO-d_6, δ_C, ppm):

endo-isomer: 25.4, 29.8, 49.1, 50.3, 54.2, 67.2, 74.1, 115.8 (d, J_{CF} = 23.0 Hz), 120.0 (d, J_{CF} = 17.5 Hz), 124.7 (d, J_{CF} = 4.0 Hz), 131.5, 133.0 (d, J_{CF} = 9.9 Hz), 150.3, 157.3 (d, J_{CF} = 254.0 Hz), 167.1, 169.4, 175.2, 176.7;

exo-isomer: 25.0, 27.1, 44.9, 46.5, 58.0, 67.6, 70.2, 115.7 (d, J_{CF} = 23.3 Hz), 119.0 (d, J_{CF} = 21.0 Hz), 124.6 (d, J_{CF} = 3.5 Hz), 131.4, 132.7 (d, J_{CF} = 11.3 Hz), 150.1, 158.7 (d, J_{CF} = 265.6 Hz), 169.8, 170.7, 174.4, 175.0.

^{19}F NMR (376 MHz, DMSO-d_6, δ_F, ppm): -114.4, -114.3.

HRMS (ESI): m/z [M - H]$^+$ calculated for $C_{18}H_{14}ClFN_4O_5$: 419.0564; found: 419.0575.

(*rac*)-(*endo,exo*)-2'-(3-Nitrophenyl)hexahydro-1'*H*,2*H*-spiro[pyrimidine-5,4'-pyrrolo[3,4-*a*]pyrrolizine]-1',2,3',4,6(1*H*,2'*H*,3*H*)-pentaone (4k)

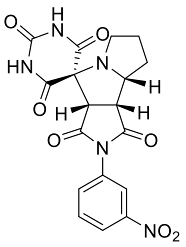

4k 30%
endo:exo 1:0.14

The synthesis was carried out according to procedure **A**. Alloxan (**1**, 0.072 g, 0.45 mmol), L-proline (**2**, 0.106 g, 0.9 mmol) and *N*-(3-nitrophenyl)maleimide (**3k**, 0.100 g, 0.45 mmol) were mixed with 3 mL of dried 1,4-dioxane and heated under stirring in a closed tube at 100 °C for 16 hours under argon atmosphere. The reaction mixture was filtered off and filtrate was evaporated under reduced pressure. The resulting residue was dissolved in methanol, the methanol solution was evaporated on an electric hotplate and $CHCl_3$ was gradually added. The addition of $CHCl_3$ was stopped when the solution became cloudy. This solution was placed in a refrigerator for several hours. After this time a precipitate was formed in the solution, which was filtered on a Schott filter and dried. White solid. Yield: 30% (54 mg).

^{1}H NMR (400 MHz, DMSO-d_6, δ, ppm):

endo-isomer: 1.68-2.06 (m, 4H), 2.57-2.76 (m, 2H), 3.83 (dd, J = 8.4 Hz, J = 8.5 Hz, 1H), 4.09 (ddd, J = 8.4 Hz, J = 7.7 Hz, J = 7.8 Hz, 1H), 4.21 (d, J = 8.5 Hz, 1H), 7.75 (bd, J = 8.4 Hz, 1H), 7.85 (t, J = 8.1 Hz, 1H), 8.17 (dd, J = 2.0 Hz, J = 2.1 Hz, 1H), 8.32 (bd, J = 8.3 Hz, 1H), 11.52-12.1 (m, 2H);

exo-isomer: 1.68-2.06 (m, 3H), 2.09-2.21 (m, 1H), 2.57-2.76 (m, 2H), 3.75 (dd, J = 7.0 Hz, J = 10.4 Hz, 1H), 3.86-3.91 (m, 1H), 4.58 (d, J = 10.4 Hz, 1H), 7.75 (bd, J = 8.4 Hz, 1H), 7.85 (t, J = 8.1 Hz, 1H), 8.12 (t, J = 2.1 Hz, J = 2.2 Hz, 1H), 8.32 (bd, J = 8.3 Hz, 1H), 11.52-12.1 (m, 2H).

^{13}C NMR (101 MHz, DMSO-d_6, δ_C, ppm):

The major (*endo*-) isomer: 24.8, 27.0, 44.9, 46.7, 57.9, 67.5, 70.2, 122.0,123.8, 131.1, 133.6, 133.8, 148.4, 150.0, 169.8, 170.6, 174.6, 175.1.

HRMS (ESI): m/z [M + H]$^+$ calculated for $C_{18}H_{15}N_5O_7$: 414.1044; found: 414.1043.

(*rac*)-(*endo,exo*)-2'-(3-Chlorophenyl)hexahydro-1'*H*,2*H*-spiro[pyrimidine-5,4'-pyrrolo[3,4-*a*]pyrrolizine]-1',2,3',4,6(1*H*,2'*H*,3*H*)-pentaone (4l)

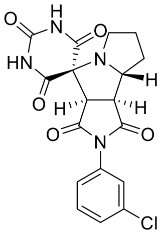

4l 25%
endo:exo 1:2.5

The synthesis was carried out according to procedure **A**. Alloxan (**1**, 0.077 g, 0.48 mmol), L-proline (**2**, 0.111 g, 0.97 mmol) and *N*-(3-chlorophenyl)maleimide (**3l**, 0.100 g, 0.48 mmol) were mixed with 3 mL of dried 1,4-dioxane and heated under stirring in a closed tube at 100 °C for 16 hours under argon atmosphere. The reaction mixture was filtered off and filtrate was evaporated under reduced pressure. The obtained residue was dissolved in 1–2 mL of acetone and subjected to a plate for PTLC, using a mixture of CH_2Cl_2:MeOH (15:1) as eluent. After chromatography, an oil-like product was formed which crystallized on addition of a small amount of Et_2O. White solid. Yield: 25% (48 mg).

^{1}H NMR (400 MHz, DMSO-d_6, δ, ppm):

exo-isomer: 1.65-2.03 (m, 3H), 2.14 (m, 1H), 2.62-2.76 (m, 2H), 3.70 (dd, *J* = 6.8 Hz, *J* = 10.6 Hz, 1H), 3.81-3.89 (m, 1H), 4.54 (d, *J* = 10.6 Hz, 1H), 7.20-7.36 (m, 2H), 7.50-7.61 (m, 2H), 11.91 (bs, 2H);

endo-isomer: 1.65-2.03 (m, 4H), 2.62-2.76 (m, 2H), 3.76-3.80 (m, 1H), 4.02 (ddd, *J* = 7.8 Hz, *J* = 8.0 Hz, *J* = 8.1 Hz, 1H), 4.16 (d, *J* = 8.8 Hz, 1H), 7.20-7.36 (m, 2H), 7.50-7.61 (m, 2H), 11.91 (bs, 2H).

^{13}C NMR (101 MHz, DMSO-d_6, δ_C, ppm):

exo-isomer: 25.4, 29.8, 49.1, 50.3, 54.2, 67.2, 74.0, 126.7, 127.1, 129.0, 131.23, 133.5, 134.2, 150.0, 167.0, 169.3, 175.4, 177.0;

endo-isomer: 25.1, 27.1, 44.9, 46.5, 58.1, 67.6, 70.2, 126.2, 127.0, 129.0, 131.19, 133.5, 134.2, 149.97, 169.9, 170.8, 174.6, 175.2.

HRMS (ESI): m/z [M + H]$^+$ calculated for $C_{18}H_{15}ClN_4O_5$: 403.0804; found: 403.0805.

(*rac*)-(*endo,exo*)-2'-(3-(trifluoromethyl)phenyl)hexahydro-1'*H*,2*H*-spiro[pyrimidine-5,4'-pyrrolo[3,4-*a*]pyrrolizine]-1',2,3,3',4,6(1*H*,2'*H*,3*H*)-pentaone (4m)

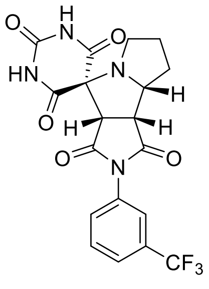

4m 50%
endo:exo 1:0.45

The synthesis was carried out according to procedure **A**. Alloxan (**1**, 0.066 g, 0.42 mmol), L-proline (**2**, 0.095 g, 0.83 mmol) and *N*-(3-(trifluoromethyl)phenyl)maleimide (**3m**, 0.100 g, 0.42 mmol) were mixed with 3 mL of dried 1,4-dioxane and heated under stirring in a closed tube at 100 °C for 16 hours under argon atmosphere. The reaction mixture was filtered off and filtrate was evaporated under reduced pressure. The obtained residue was dissolved in 1–2 mL of acetone and subjected to a plate for PTLC, using a mixture of CH_2Cl_2:MeOH (20:1) as eluent. After chromatography, an oil-like product was formed which crystallized on addition of a small amount of Et_2O. White solid. Yield: 50% (91 mg).

^{1}H NMR (400 MHz, DMSO-d_6, δ, ppm):

endo-isomer: 1.67-2.03 (m, 4H), 2.59-2.76 (m, 2H), 3.82 (dd, *J* = 8.5 Hz, *J* = 8.7 Hz, 1H), 4.03 (ddd, *J* = 6.9 Hz, *J* = 7.5 Hz, *J* = 8.5 Hz, 1H), 4.19 (d, *J* = 8.7 Hz, 1H), 7.54-7.68 (m, 2H), 7.73-7.89 (m, 2H);

exo-isomer: 1.67-2.03 (m, 3H), 2.06-2.22 (m, 1H), 2.59-2.76 (m, 2H), 3.72 (dd, *J* = 7.0 Hz, *J* = 10.5 Hz, 1H), 3.85-3.90 (m, 1H), 4.57 (d, *J* = 10.5 Hz, 1H), 7.54-7.68 (m, 2H), 7.73-7.89 (m, 2H), 11.80 (bs, 2H).

^{13}C NMR (101 MHz, DMSO-d_6, δ_C, ppm): 24.9 (endo), 25.4 (exo), 27.1 (endo), 29.8 (exo), 44.9 (endo), 46.6 (endo), 49.1 (exo), 50.3 (exo), 54.3 (exo), 58.0 (endo), 67.1 (exo), 67.6 (endo), 70.2 (endo), 74.1

(exo), 123.76 (exo), 123.81 (endo), 124.16 (q, J_{CF} = 271 Hz), 125.51 (exo), 125.70 (endo), 130.17 (q, J_{CF} = 30 Hz), 130.90 (endo), 130.96 (exo), 131.46 (endo), 131.58 (exo), 133.4 (endo), 133.6 (exo), 149.98 (endo), 150.00 (endo), 174.7 (endo), 175.3 (endo), 175.4 (exo), 177.0 (exo).

^{19}F NMR (376 MHz, DMSO-d_6, δ_F, ppm): -61.25, -61.22.

HRMS (ESI): m/z [M + H]$^+$ calculated for $C_{19}H_{15}F_3N_4O_5$: 437.1067; found: 437.1063.

(*rac*)-(*endo,exo*)-2'-(*m*-Tolyl)hexahydro-1'*H*,2*H*-spiro[pyrimidine-5,4'-pyrrolo[3,4-*a*]pyrrolizine]1',2,3',4,6(1*H*,2'*H*,3*H*)-pentaone (4n)

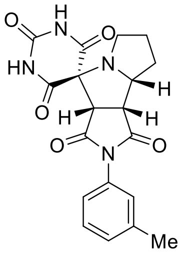

4n 59%
endo:exo 1:0.8

The synthesis was carried out according to procedure **A**. Alloxan (**1**, 0.085 g, 0.53 mmol), L-proline (**2**, 0.123 g, 1.07 mmol) and *N*-(*m*-tolyl)maleimide (**3n**, 0.100 g, 0.53 mmol) were mixed with 3 mL of dried 1,4-dioxane and heated under stirring in a closed tube at 100 °C for 16 hours under nitrogen atmosphere. The reaction mixture was filtered off and filtrate was evaporated under reduced pressure. The residue was subjected to silica gel and purified by preparative column chromatography using a mixture of CH_2Cl_2:MeOH (30:1) as eluent. White solid. Yield: 59% (121 mg). There are the signals of Et_2O, as cocrystallysed solvent, in spectra.

^{1}H NMR (400 MHz, DMSO-d_6, δ, ppm):

endo-isomer: 1.67-2.03 (m, 4H), 2.31-2.39 (m, 3H), 2.55-2.76 (m, 2H), 3.79 (dd, *J* = 8.5 Hz, *J* = 10.3 Hz, 1H), 4.00-4.10 (m, 1H), 4.15 (d, *J* = 8.8 Hz, 1H), 6.99-7.09 (m, 2H), 7.25 (d, *J* = 7.8 Hz, 1H), 7.39 (t, J = 7.6 Hz, 1H), 11.79 (bs, 2H);

exo-isomer: 1.67-2.03 (m, 3H), 2.06-2.22 (m, 1H), 2.31-2.39 (m, 3H), 2.59-2.76 (m, 2H), 3.67 (dd, *J* = 7.0 Hz, *J* = 10.7 Hz, 1H), 3.83-3.90 (m, 1H), 4.53 (d, *J* = 10.6 Hz, 1H), 6.99-7.09 (m, 2H), 7.25 (d, *J* = 7.8 Hz, 1H), 7.39 (t, J = 7.6 Hz, 1H), 11.79 (bs, 2H).

^{13}C NMR (101 MHz, DMSO-d_6, δ_C, ppm): 21.3 (endo), 25.4 (endo), 25.5 (exo), 27.2 (endo), 29.8 (exo), 44.9 (endo), 46.4 (endo), 49.0 (exo), 50.5 (exo), 54.2 (exo), 58.4 (endo), 67.2 (exo), 67.7 (endo), 70.3 (endo), 73.8 (exo), 124.5 (endo), 124.6 (exo), 127.7 (endo), 127.8 (exo), 129.19 (endo), 129.23 (exo), 129.5 (exo), 129.6 (endo), 132.8 (endo), 132.9 (exo), 138.8 (endo), 138.9 (exo), 149.94 (endo), 149.98 (exo), 167.1, 169.1, 169.8, 171.1, 174.8, 175.5, 175.6, 177.2.

HRMS (ESI): m/z [M + H]$^+$ calculated for $C_{19}H_{18}N_4O_5$: 383.1350; found: 383.1352.

(*rac*)-(*endo,exo*)-2'-(3-Methoxyphenyl)hexahydro-1'*H*,2*H*-spiro[pyrimidine-5,4'-pyrrolo[3,4-*a*]pyrrolizine]-1',2,3',4,6(1*H*,2'*H*,3*H*)-pentaone (4o)

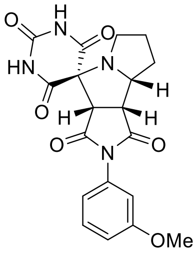

4o 40%
endo:exo 1:0.4

The synthesis was carried out according to procedure **A**. Alloxan (**1**, 0.079 g, 0.49 mmol), L-proline (**2**, 0.113 g, 0.98 mmol) and *N*-(3-methoxyphenyl)maleimide (**3o**, 0.100 g, 0.49 mmol) were mixed with 3 mL of dried 1,4-dioxane and heated under stirring in a closed tube at 100 °C for 16 hours under nitrogen atmosphere. The reaction mixture was filtered off and filtrate was evaporated under reduced pressure. The residue was subjected to silica gel and purified by preparative column chromatography using a mixture of CH_2Cl_2:MeOH (30:1) as eluent. White solid. Yield: 40% (78 mg).

^{1}H NMR (400 MHz, DMSO-d_6, δ, ppm):

endo-isomer: 1.69-2.04 (m, 4H), 2.55-2.76 (m, 2H), 3.74-3.81 (m, 1H), 3.80 (bs, 3H), 3.99-4.09 (m, 1H), 4.14 (d, *J* = 8.9 Hz, 1H), 7.00-7.09 (m, 2H), 7.10-7.21 (m, 2H), 11.74 (bs, 2H);

exo-isomer: 1.69-2.04 (m, 3H), 2.06-2.19 (m, 1H), 2.55-2.76 (m, 2H), 3.65 (dd, *J* = 6.9 Hz, *J* = 10.6 Hz, 1H), 3.80 (bs, 3H), 3.82-3.87 (m, 1H), 4.51 (d, *J* = 10.5 Hz, 1H), 7.00-7.09 (m, 2H), 7.10-7.21 (m, 2H), 11.74 (bs, 2H).

^{13}C NMR (101 MHz, DMSO-d_6, δ_C, ppm): 25.4 (endo), 25.5 (exo), 27.2 (endo), 29.8 (exo), 44.9 (endo), 46.3 (endo), 49.0 (exo), 50.5 (exo), 54.1 (exo), 55.9 (endo/exo), 58.4 (endo), 67.2 (exo), 67.8 (endo), 70.3 (endo), 73.8 (exo), 114.6 (endo), 114.7 (exo), 125.4 (endo), 125.5 (exo), 128.55 (endo), 128.62 (exo), 149.95 (endo), 150.00 (exo), 159.5, 169.8, 171.1, 175.0, 175.7, 177.4.

HRMS (ESI): m/z [M + H]$^+$ calculated for $C_{19}H_{18}N_4O_6$: 399.1300; found: 399.1301.

(*rac*)-(*endo,exo*)-2'-(2-Methoxyphenyl)hexahydro-1'*H*,2*H*-spiro[pyrimidine-5,4'-pyrrolo[3,4-*a*]pyrrolizine]-1',2,3',4,6(1*H*,2'*H*,3*H*)-pentaone (4p)

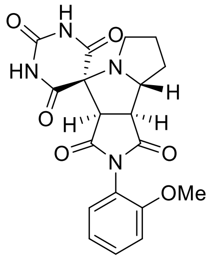

4p 26%
endo:exo 1:2.5

The synthesis was carried out according to procedure **A**. Alloxan (**1**, 0.079 g, 0.49 mmol), L-proline (**2**, 0.113 g, 0.98 mmol) and *N*-(2-methoxyphenyl)maleimide (**3p**, 0.100 g, 0.49 mmol) were mixed with 3 mL of dried 1,4-dioxane and heated under stirring in a closed tube at 100 °C for 16 hours under nitrogen atmosphere. The reaction mixture was filtered off and filtrate was evaporated under reduced pressure. The residue was subjected to silica gel and purified by preparative column chromatography using a mixture of CH_2Cl_2:MeOH (30:1) as eluent. White solid. Yield: 26% (51 mg). There are the signals of Et_2O and CH_2Cl_2, as cocrystallysed solvents, in spectra.

Main exo-isomer (Signals of atropisomers **4p** are present on the spectra):

^{1}H NMR (400 MHz, DMSO-d_6, δ, ppm): 1.65-2.20 (m, 4H), 2.53-2.76 (m, 2H), 3.74 (s, 3H), 3.77-3.84 (m, 1H), 3.84-3.90 (m, 1H), 4.58 (d, *J* = 10.3 Hz, 1H), 6.97-7.21 (m, 3H), 7.38-7.49 (m, 1H), 11.88 (bs, 2H);

^{13}C NMR (101 MHz, DMSO-d_6, δ_C, ppm): 25.4, 29.9, 49.1, 50.3, 54.1, 56.2, 67.2, 74.0, 112.9, 120.9, 121.3, 129.24, 130.9, 150.1, 155.4, 167.0, 169.2, 175.3, 176.9.

HRMS (ESI): m/z [M + H]$^+$ calculated for $C_{19}H_{18}N_4O_6$: 399.1299 found: 399.1297.

Formation murexide from alloxan *via* Strecker degradation

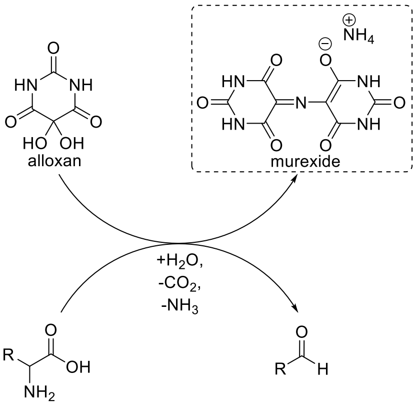

Scheme S1: Murexide formation during Strecker degradation of alloxan

During the 1,3-dipolar cycloaddition reaction of an azomethine ylide, generated from alloxan and an amino acid, with *N*-substituted maleimides, a purple-colored by-product – murexide – is formed (Scheme S1). This phenomenon was described in the work by grigg et al., 1994 [1]. The formation of murexide is based on the Strecker degradation [2]. This process represents a redox reaction in which alloxan is oxidized to murexide, while the amino acid is reduced to an aldehyde, simultaneously undergoing decarboxylation and deamination.

1. Aly, M. F.; El-Nagger, g. M.; El-Emary, T. I.; grigg, R.; Metwally, S. A. M.; Sivagnanam, S. Tetrahedron 1994, 50, 895–906. doi: 10.1016/S0040-4020(01)80804-8
2. Strecker, A. *Justus Liebigs Ann. Chem.* **1862**, *123*, 363–365. doi: 10.1002/jlac.18621230312

The ^{1}H NMR, ^{13}C NMR, ^{19}F NMR, ^{13}C DEPT NMR, COSY ^{1}H,^{1}H NMR, HSQC ^{1}H,^{13}C NMR spectra of compounds 4a–p

Table S1. Chemical shifts, signal forms and spin–spin coupling constants of methine protons of compounds **4a**–**p** in ^{1}H NMR spectra.

	C<u>H</u>^{4} (*endo*)	C<u>H</u>^{5} (*endo*)	C<u>H</u>^{6} (*endo*)	C<u>H</u>^{4} (*exo*)	C<u>H</u>^{5} (*exo*)	C<u>H</u>^{6} (*exo*)
	δ ppm, J_{HH}					
**4a**	3.94 (d, *J* = 7.9 Hz)	3.52 (dd, *J* = 8.6 Hz, *J* = 8.8 Hz)	3.92 (d, *J* = 8.8 Hz)	-	-	-
**4b**	3.93-3.98 (m)	3.57 (dd, *J* = 8.4 Hz, *J* = 8.3 Hz)	4.00 (bd, *J* = 8.4 Hz)	-	-	-
**4c**	4.04 (ddd, *J* = 6.2 Hz, *J* = 8.7 Hz, *J* = 9.0 Hz)	3.80 (dd, *J* = 8.7 Hz, *J* = 8.6 Hz)	4.20 (d, *J* = 9.0 Hz)	-	-	-
**4d**	3.97-4.08 (m)	3.78 (dd, *J* = 8.9 Hz, *J* = 8.7 Hz)	4.14 (d, *J* = 8.9 Hz)	-	-	-
**4e**	4.03 (ddd, *J* = 7.3 Hz, *J* = 8.0 Hz *J* = 8.5 Hz)	3.83 (dd, *J* = 8.7 Hz, *J* = 8.5 Hz)	4.20 (d, *J* = 8.7 Hz)	-	-	-
**4f**	3.95-4.05 (m)	3.63 (dd, *J* = 8.6 Hz, *J* = 8.7 Hz)	4.10 (d, *J* = 8.6 Hz)	3.71-3.78 (m)	3.56 (dd, *J* = 7.2 Hz, *J* = 10.7 Hz)	4.39-4.51 (m)
**4g**	3.91-4.03 (m)	3.46-3.58 (m)	3.99 (d, *J* = 8.7 Hz)	3.70-3.76 (m)	3.44-3.58 (m)	4.37 (d, *J* = 10.5 Hz)
**4h**	3.96-4.08 (m)	3.76-3.83 (m)	4.16 (d, *J* = 9.0 Hz)	3.82-3.88 (m)	3.68 (dd, *J* = 10.6 Hz, *J* = 6.9 Hz)	4.53 (d, *J* = 10.6 Hz)
**4i**	3.97-4.07 (m)	3.79 (dd, *J* = 8.4 Hz, *J* = 8.7 Hz)	4.17 (d, *J* = 8.7 Hz)	3.82-3.89 (m)	3.71 (dd, *J* = 10.8 Hz, *J* = 6.9 Hz)	4.54 (d, *J* = 10.8 Hz)
**4j**	3.98-4.06 (m)	3.76-3.82 (m)	4.17 (d, *J* = 8.9 Hz)	3.81-3.89 (m)	3.70 (dd, *J* = 10.6 Hz, *J* = 6.8 Hz)	4.54 (d, *J* = 10.6 Hz)
**4k**	4.09 (ddd, *J* = 8.4 Hz, *J* = 7.7 Hz, *J* = 7.8 Hz)	3.83 (dd, *J* = 8.4 Hz, *J* = 8.5 Hz)	4.21 (d, *J* = 8.5 Hz)	3.86-3.91 (m)	3.75 (dd, *J* = 7.0 Hz, *J* = 10.4 Hz)	4.58 (d, *J* = 10.4 Hz)
**4l**	4.02 (ddd, *J* = 7.8 Hz, *J* = 8.0 Hz, *J* = 8.1 Hz)	3.76-3.80 (m)	4.16 (d, *J* = 8.8 Hz)	3.81-3.89 (m)	3.70 (dd, *J* = 6.8 Hz, *J* = 10.6 Hz)	4.54 (d, *J* = 10.6 Hz)

**4m**	4.03 (ddd, *J* = 6.9 Hz, *J* = 7.5 Hz, *J* = 8.5 Hz)	3.82 (dd, *J* = 8.5 Hz, *J* = 8.7 Hz)	4.19 (d, *J* = 8.7 Hz)	3.85-3.90 (m)	3.72 (dd, *J* = 7.0 Hz, *J* = 10.5 Hz)	4.57 (d, *J* = 10.5 Hz)
**4n**	4.00-4.10 (m)	3.79 (dd, *J* = 8.5 Hz, *J* = 10.3 Hz)	4.15 (d, *J* = 8.8 Hz)	3.83-3.90 (m)	3.67 (dd, *J* = 7.0 Hz, *J* = 10.7 Hz)	4.53 (d, *J* = 10.6 Hz)
**4o**	3.99-4.09 (m)	3.74-3.81 (m)	4.14 (d, *J* = 8.9 Hz)	3.82-3.87 (m)	3.65 (dd, *J* = 6.9 Hz, *J* = 10.6 Hz)	4.51 (d, *J* = 10.5 Hz)
**4p**	mixture of rotamers			3.77-3.84 (m)	3.84-3.90 (m)	4.58 (d, *J* = 10.3 Hz)

(*rac*)-(3a’*S*,8a’*R*,8b’*R*)-Hexahydro-1'*H*,2*H*-spiro[pyrimidine-5,4'-pyrrolo[3,4-*a*]pyrrolizine]-1',2,3',4,6(1*H*,2'*H*,3*H*)-pentaone (**4a**)

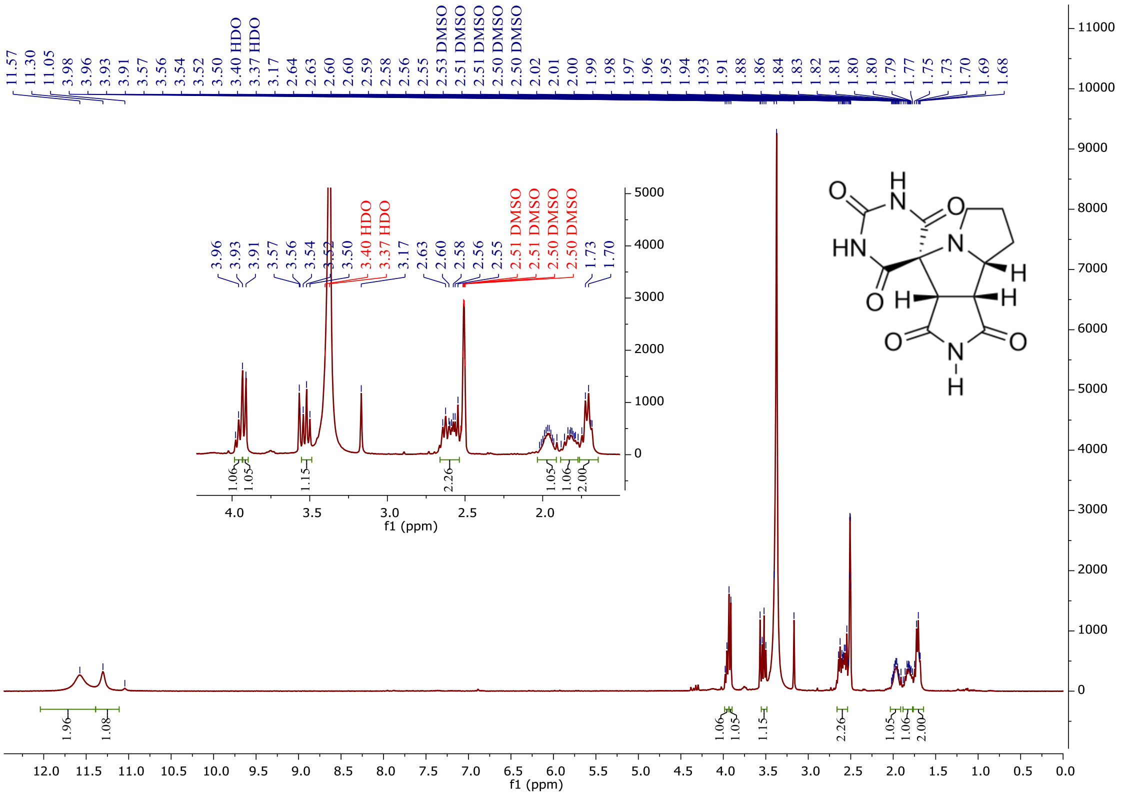

Figure S1. ^{1}H NMR spectrum (DMSO-d_6) of **4a**

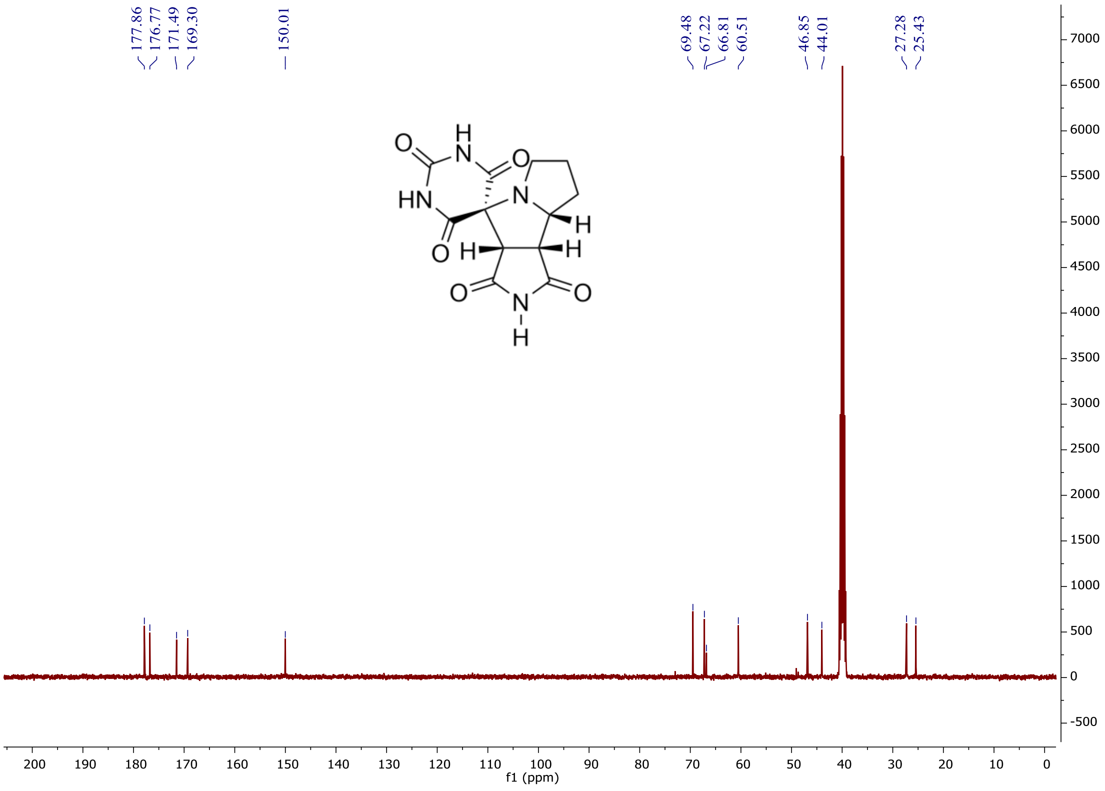

Figure S2. ^{13}C NMR spectrum (DMSO-d_6) of **4a**

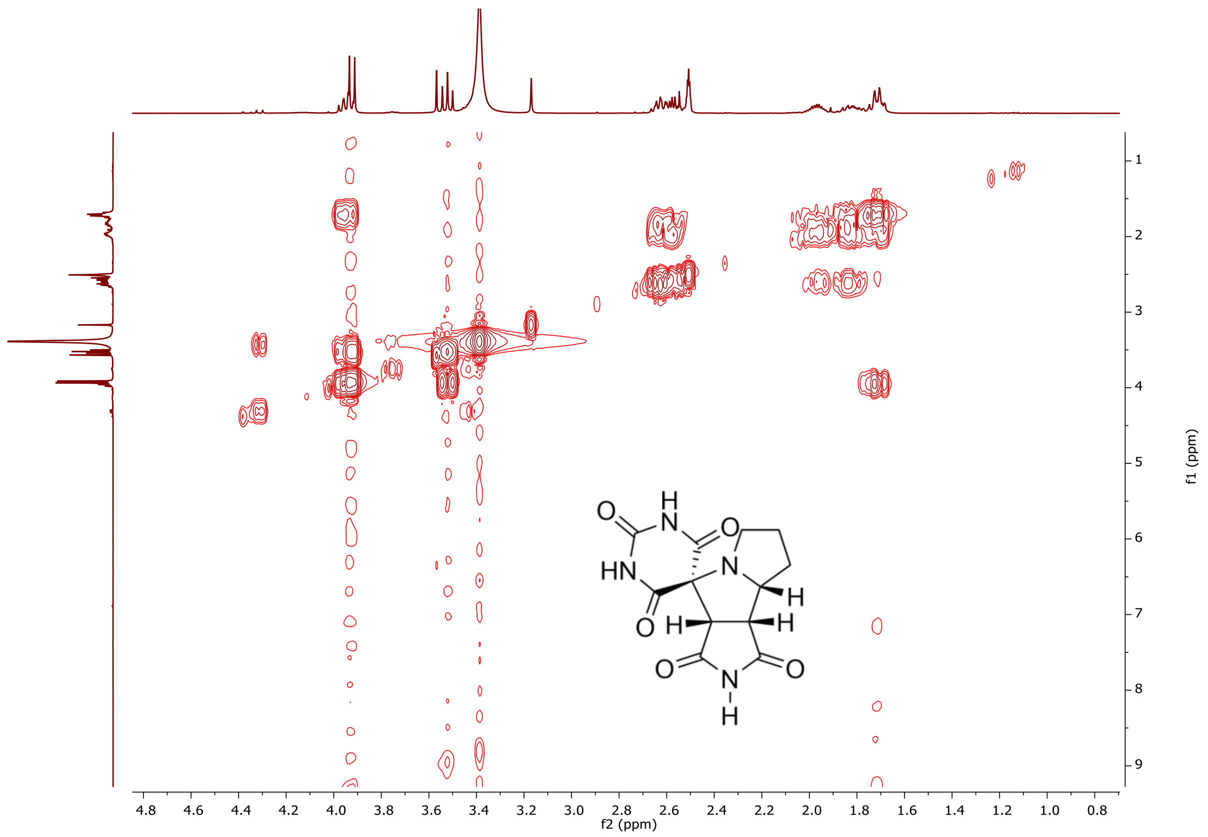

Figure S3. ^1H-^1H COSY NMR spectrum (DMSO-d_6) of **4a**

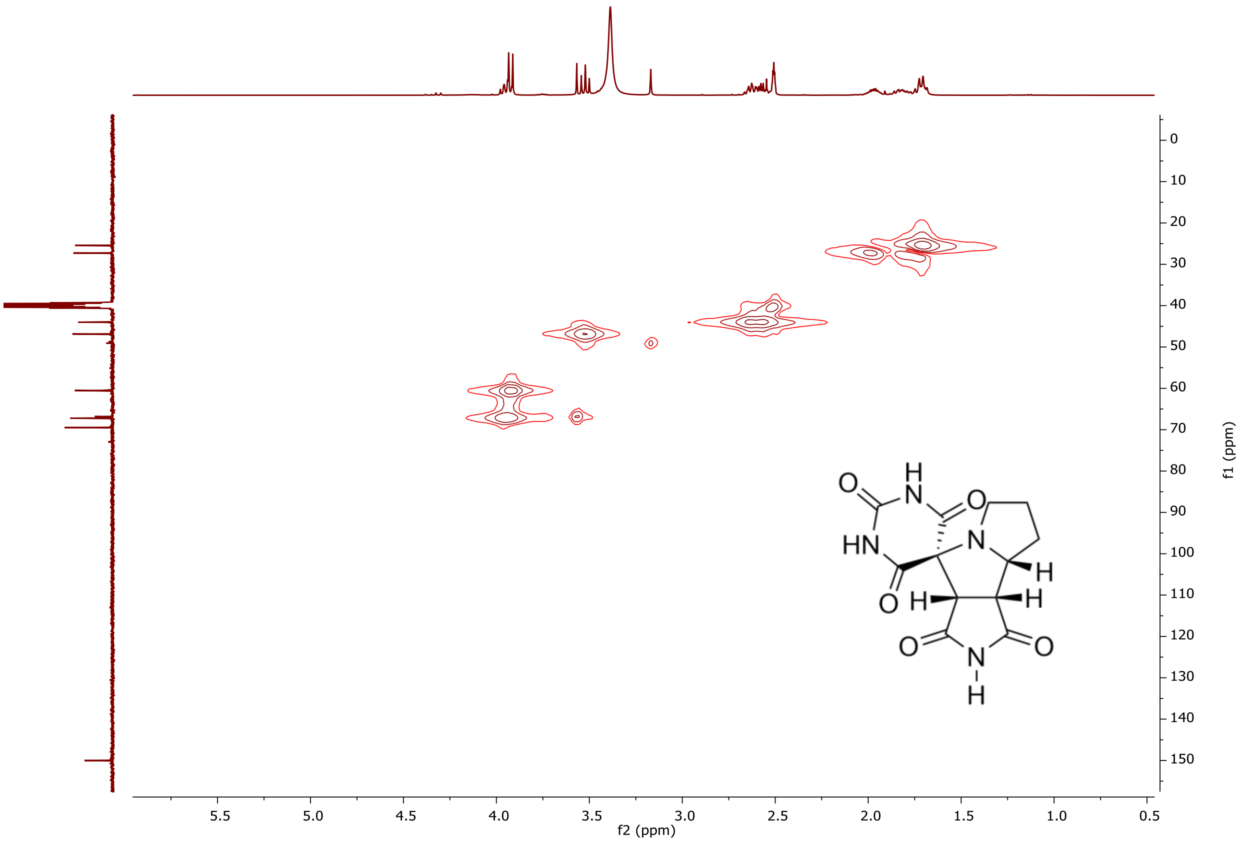

Figure S4. ^1H-^{13}C HSQC NMR spectrum (DMSO-d_6) of **4a**

(*rac*)-(3a'*S*,8a'*R*,8b'*R*)-2'-Methylhexahydro-1'*H*,2*H*-spiro[pyrimidine-5,4'-pyrrolo[3,4-*a*]pyrrolizine]-1',2,3',4,6(1*H*,2'*H*,3*H*)-pentaone (**4b**)

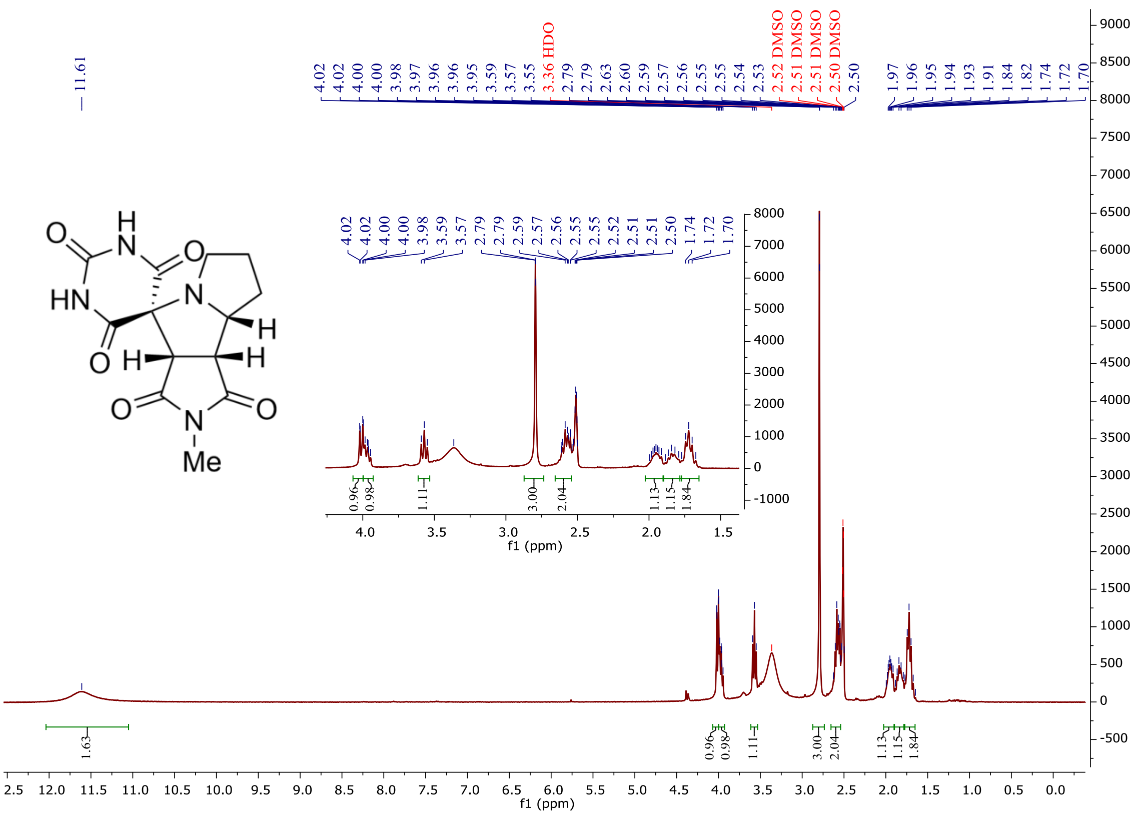

Figure S5. ^{1}H NMR spectrum (DMSO-d_6) of **4b**

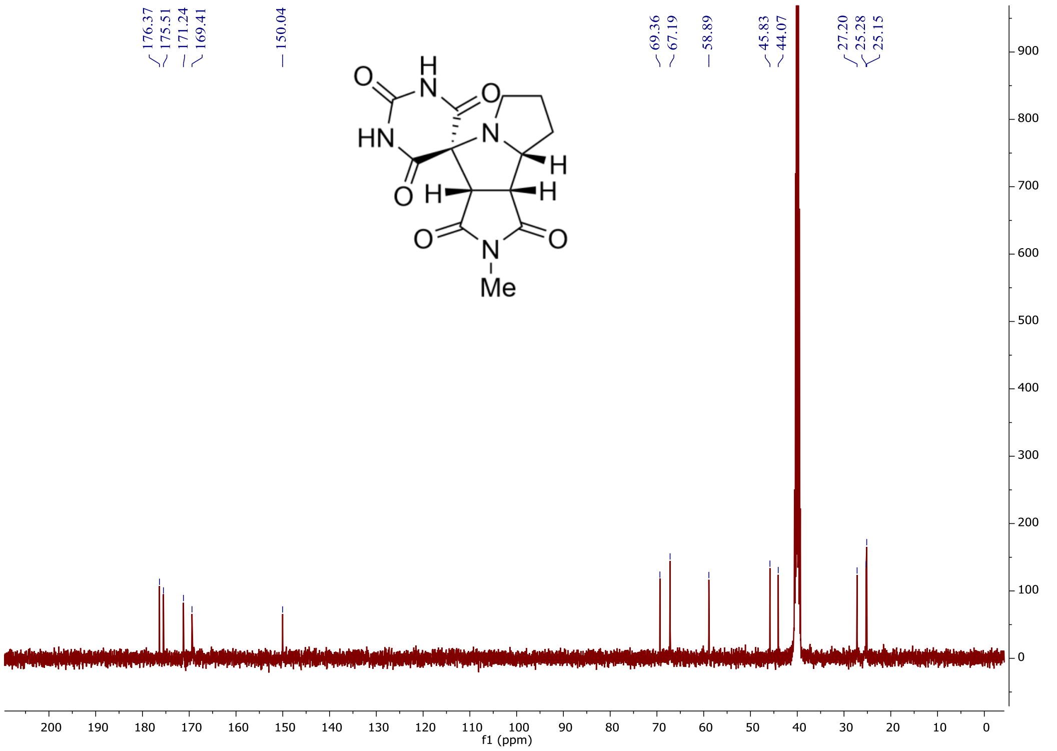

Figure S6. ^{13}C NMR spectrum (DMSO-d_6) of **4b**

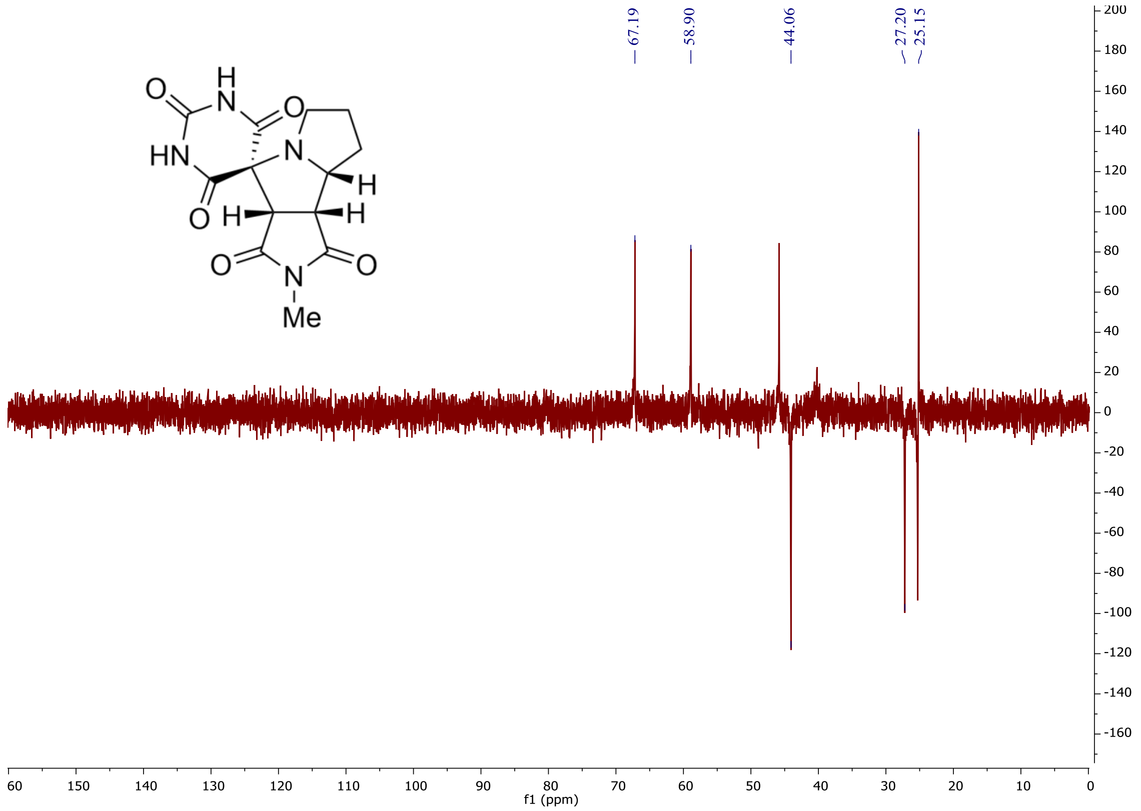

Figure S7. ^{13}C DEPT NMR spectrum (DMSO-d_6) of **4b**

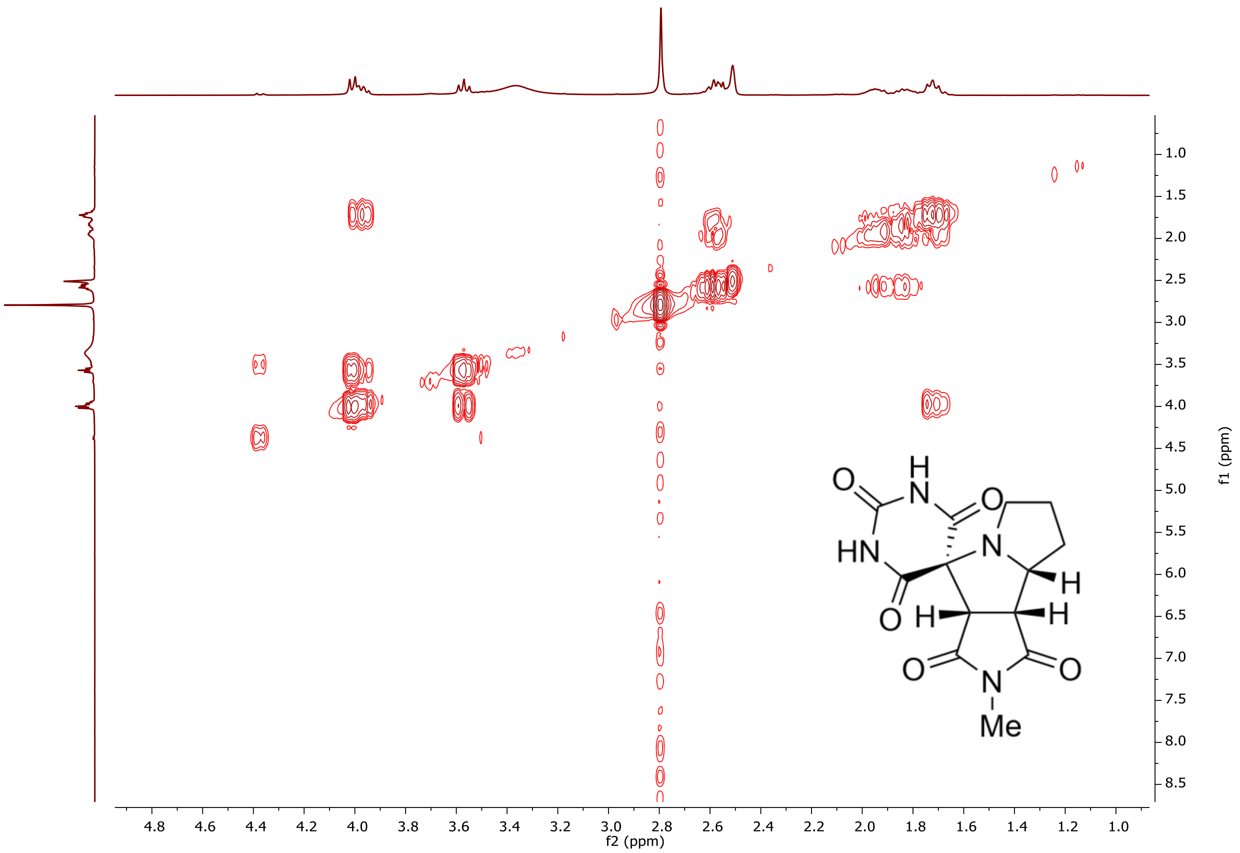

Figure S8. ^{1}H-^{1}H COSY NMR spectrum (DMSO-d_6) of **4b**

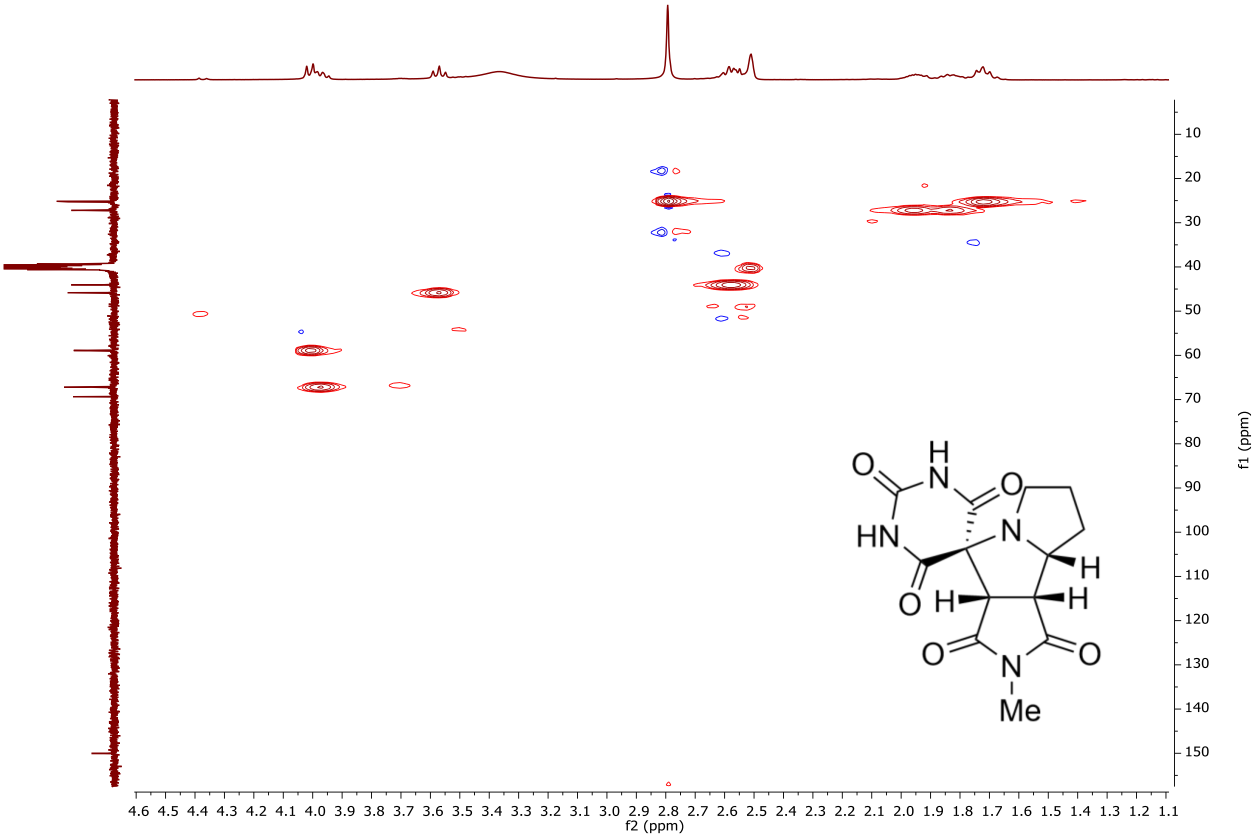

Figure S9. ^{1}H-^{13}C HSQC NMR spectrum (DMSO-d_6) of **4b**

(*rac*)-(3a'*S*,8a'*R*,8b'*R*)-2'-Phenylhexahydro-1'*H*,2*H*-spiro[pyrimidine-5,4'-pyrrolo[3,4-*a*]pyrrolizine]-1',2,3',4,6(1*H*,2'*H*,3*H*)-pentaone (**4c**)

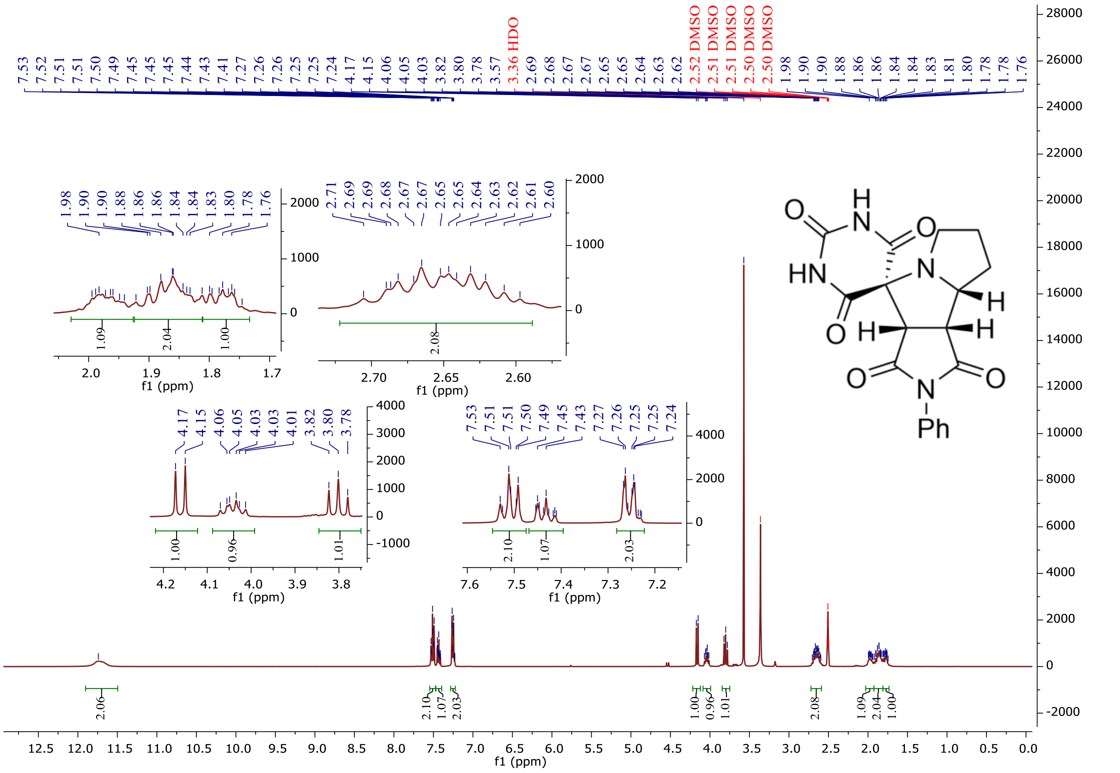

Figure S10. ^{1}H NMR spectrum (DMSO-d_6) of **4c**

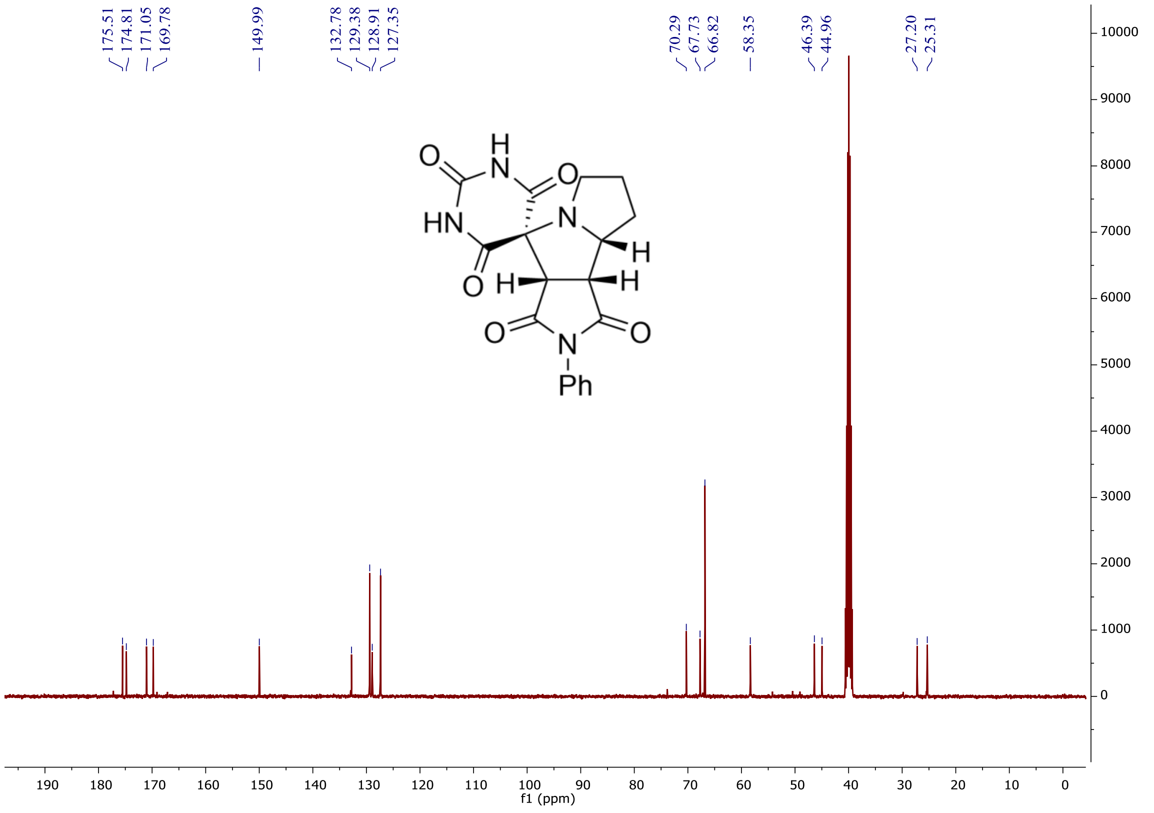

Figure S11. ^{13}C NMR spectrum (DMSO-d_6) of **4c**

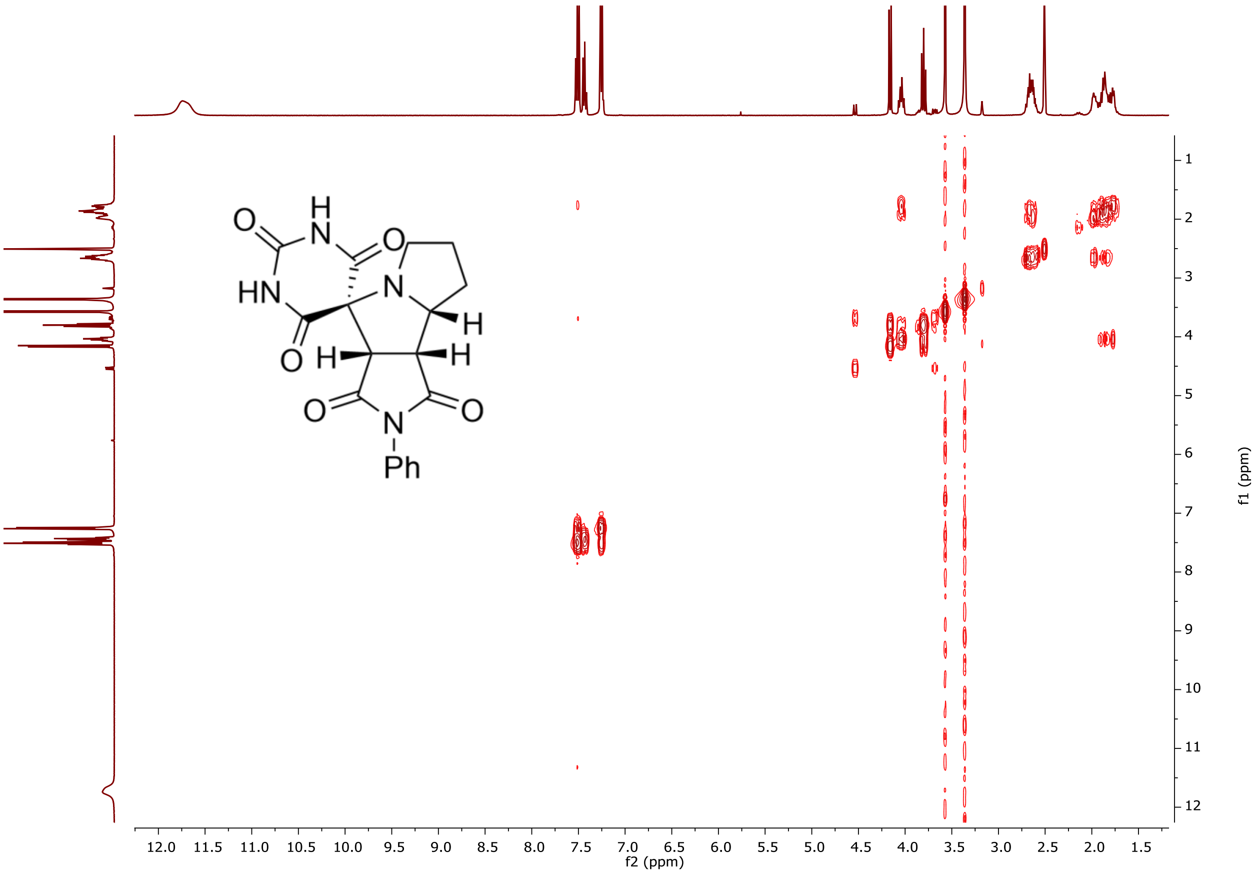

Figure S12. ^{1}H-^{1}H COSY NMR spectrum (DMSO-d_6) of **4c**

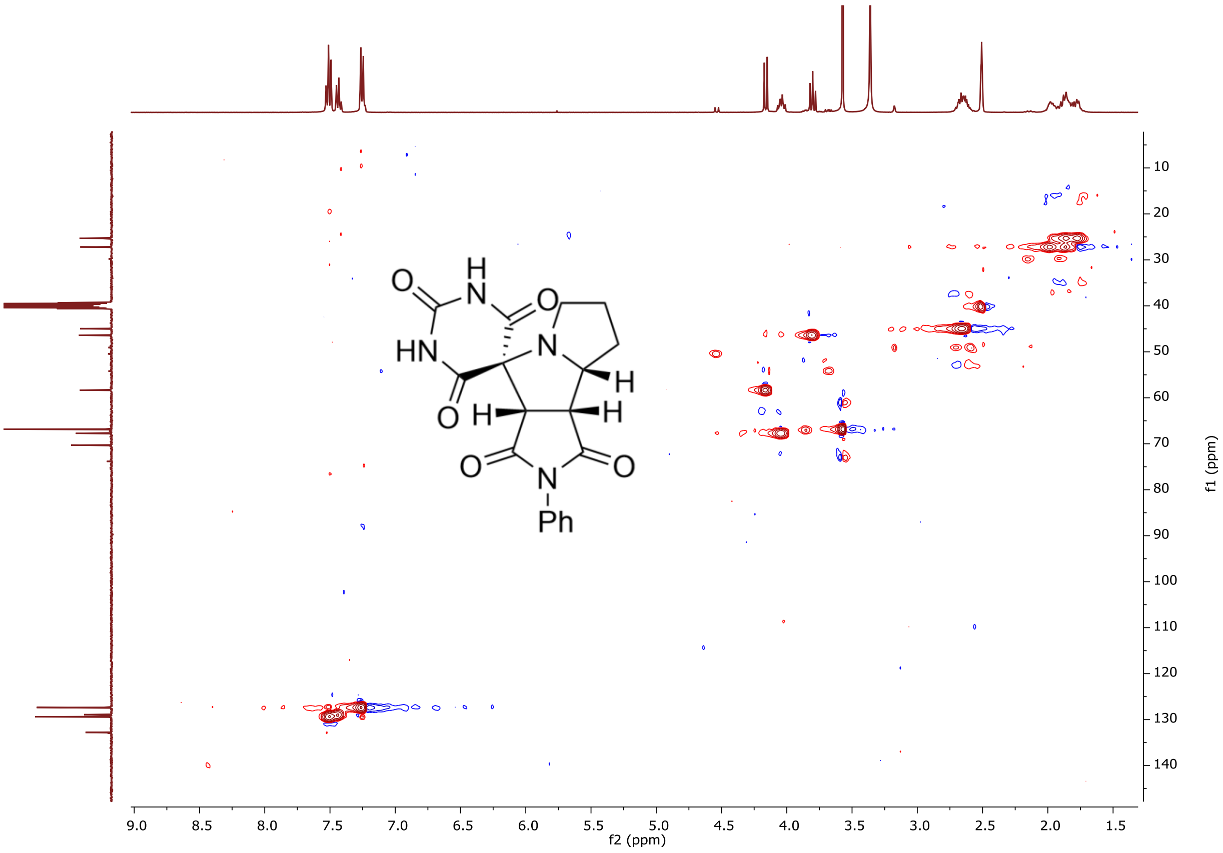

Figure S13. ^{1}H-^{13}C HSQC NMR spectrum (DMSO-d_6) of **4c**

(*rac*)-(3a'*S*,8a'*R*,8b'*R*)-2'-(*p*-Tolyl)hexahydro-1'*H*,2*H*-spiro[pyrimidine-5,4'-pyrrolo[3,3-*a*]pyrrolizine]-1',2,3',4,6(1*H*,2'*H*,3*H*)-pentaone (**4d**)

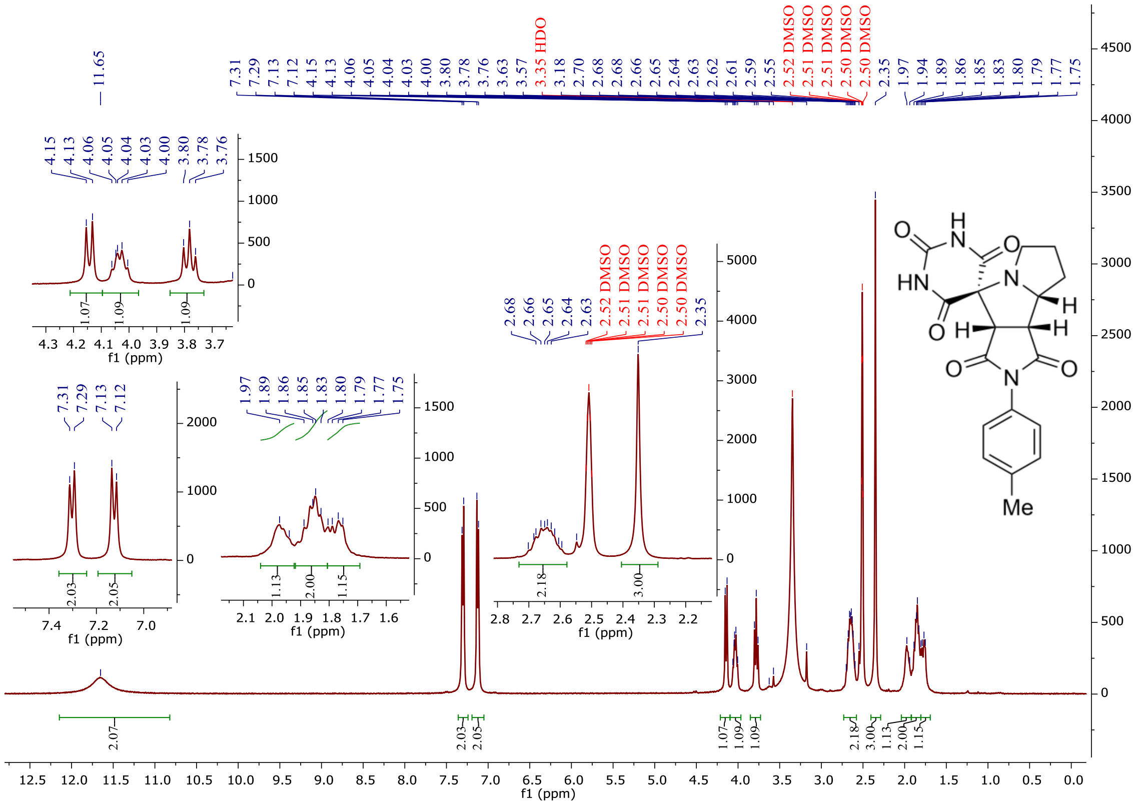

Figure S14. ^{1}H NMR spectrum (DMSO-d_6) of **4d**

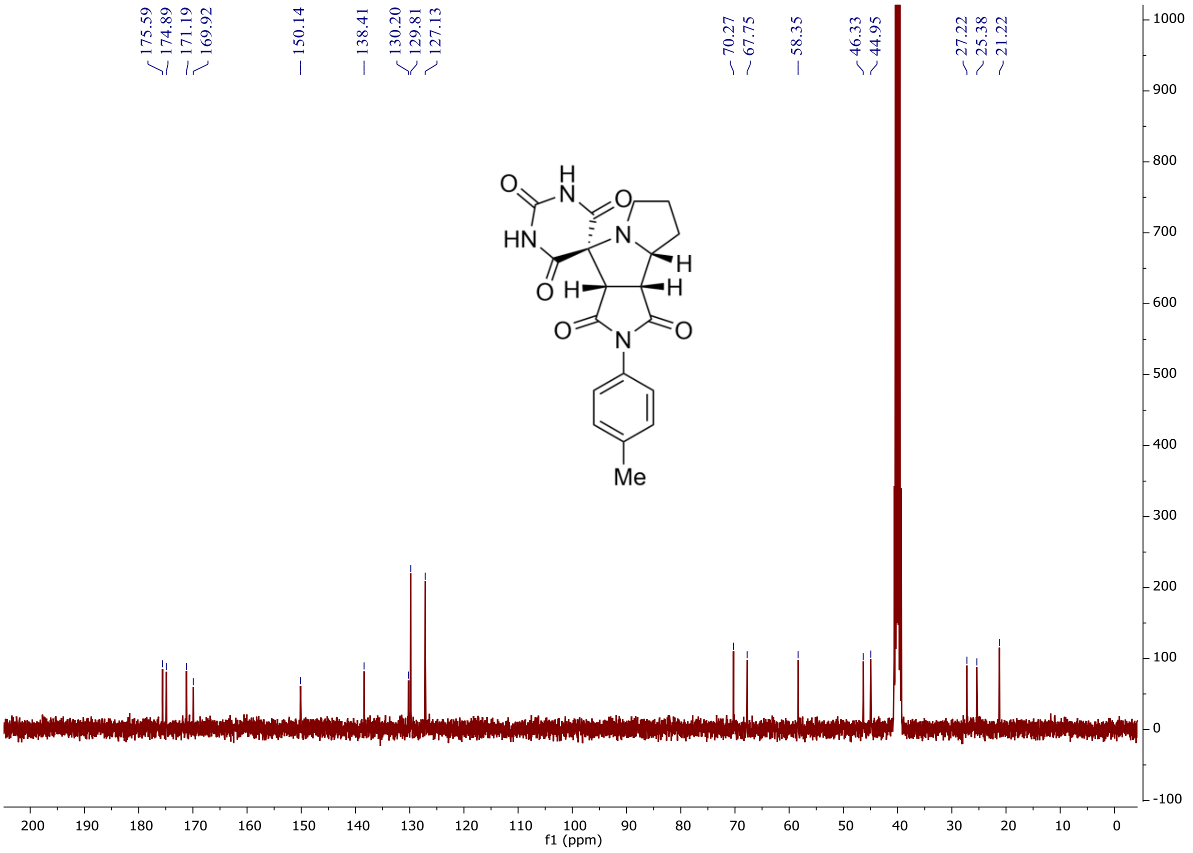

Figure S15. ^{13}C NMR spectrum (DMSO-d_6) of **4d**

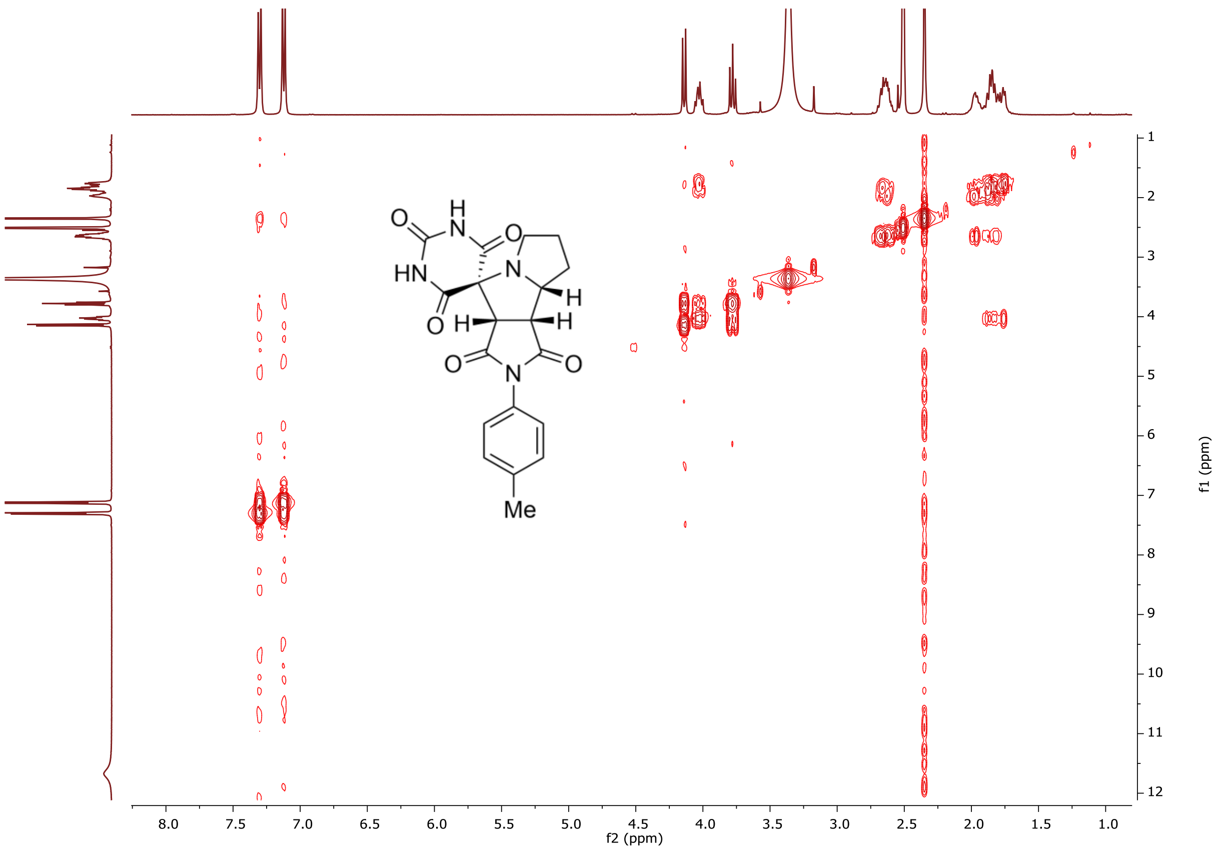

Figure S16. ^{1}H-^{1}H COSY NMR spectrum (DMSO-d_6) of **4d**

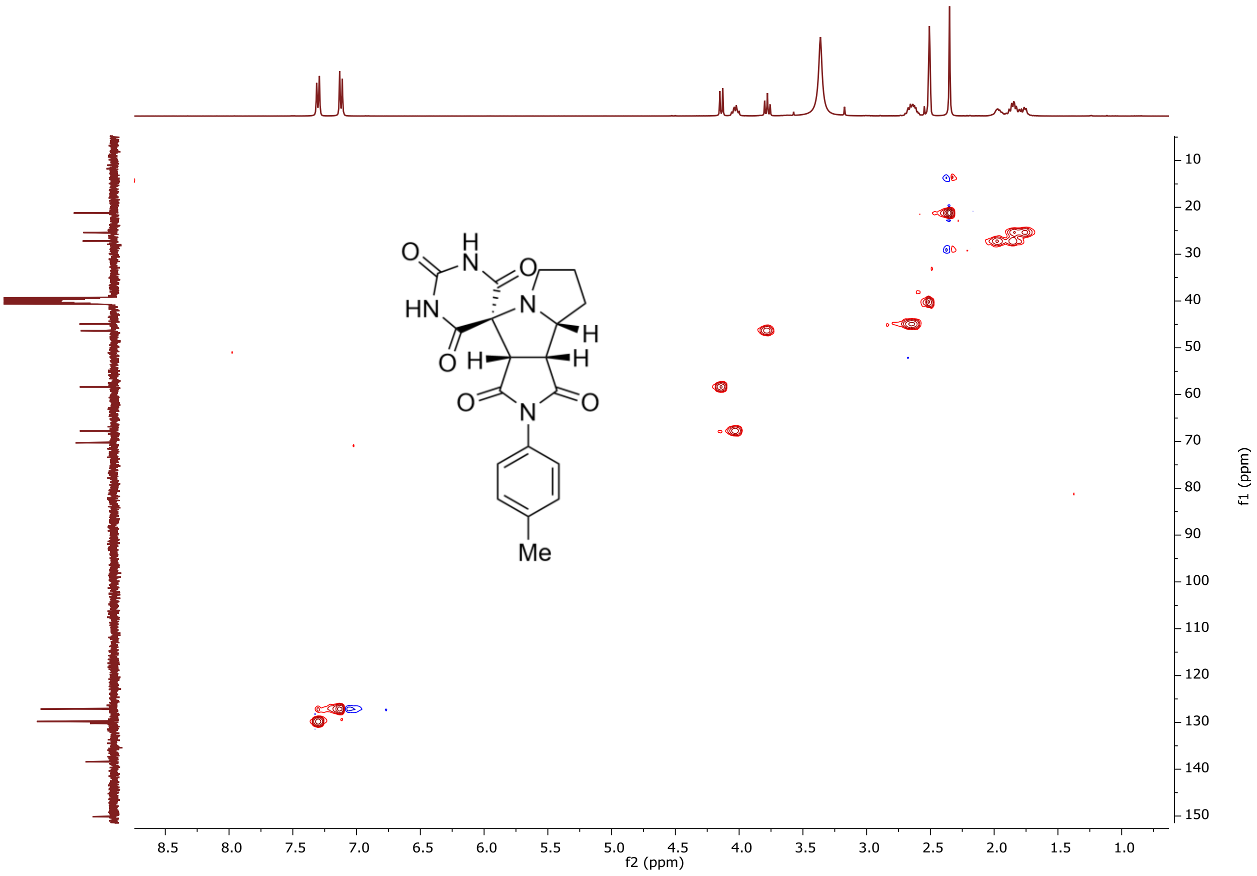

Figure S17. ^{1}H-^{13}C HSQC NMR spectrum (DMSO-d_6) of **4d**

(*rac*)-(3a′*S*,8a′*R*,8b′*R*)-2′-(4-Nitrophenyl)hexahydro-1′*H*,2*H*-spiro[pyrimidine-5,4′-pyrrolo[3,3-*a*]pyrrolizine]-1′,2,3′,4,6(1*H*,2′*H*,3*H*)-pentaone (**4e**)

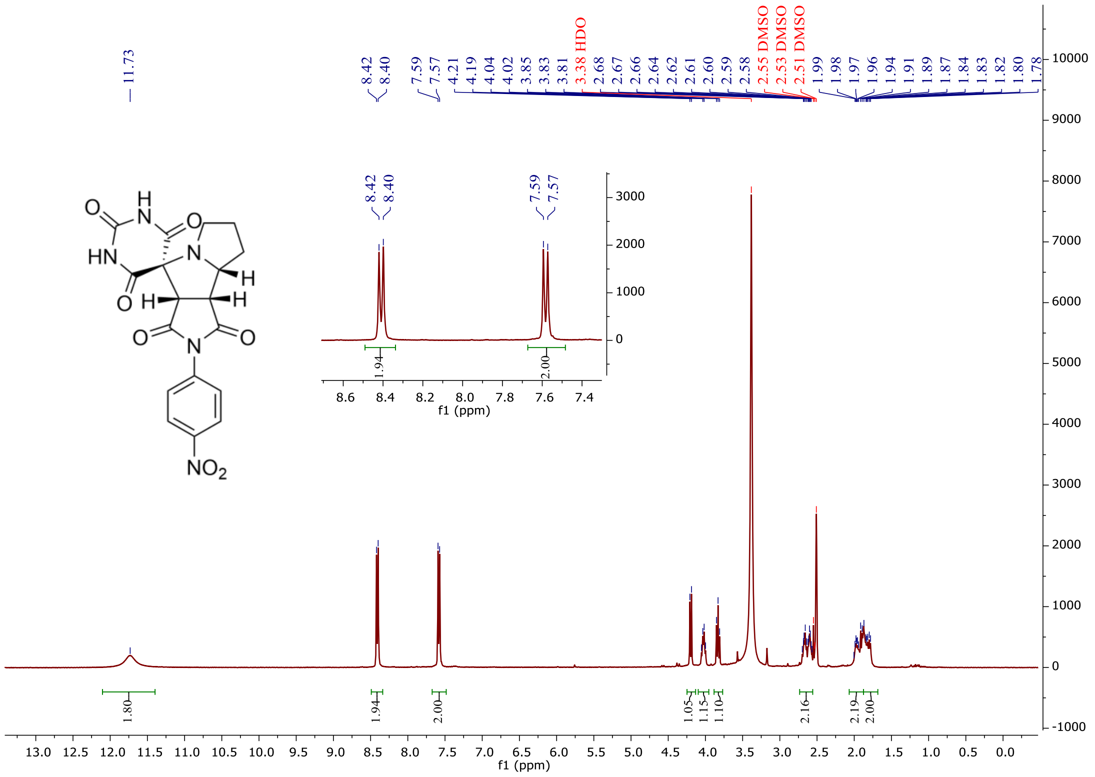

Figure S18. ^{1}H NMR spectrum (DMSO-d_6) of **4e**

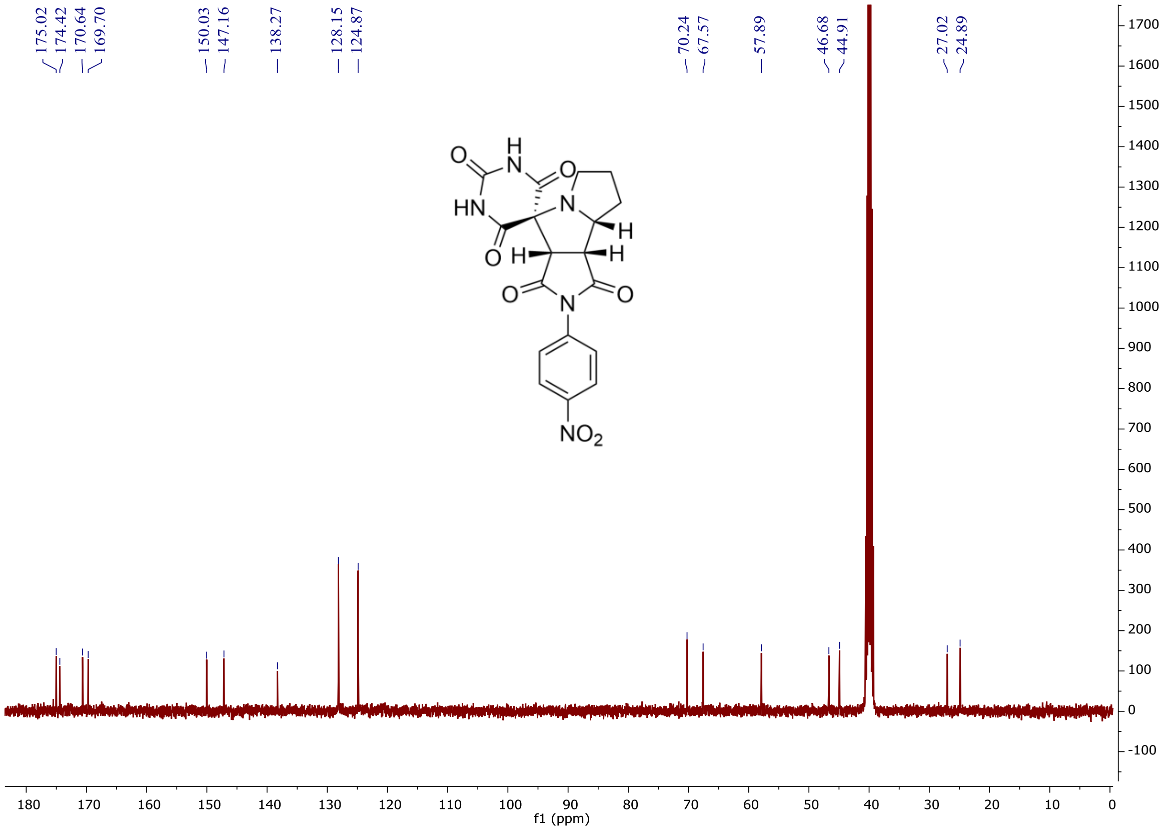

Figure S19. ^{13}C NMR spectrum (DMSO-d_6) of **4e**

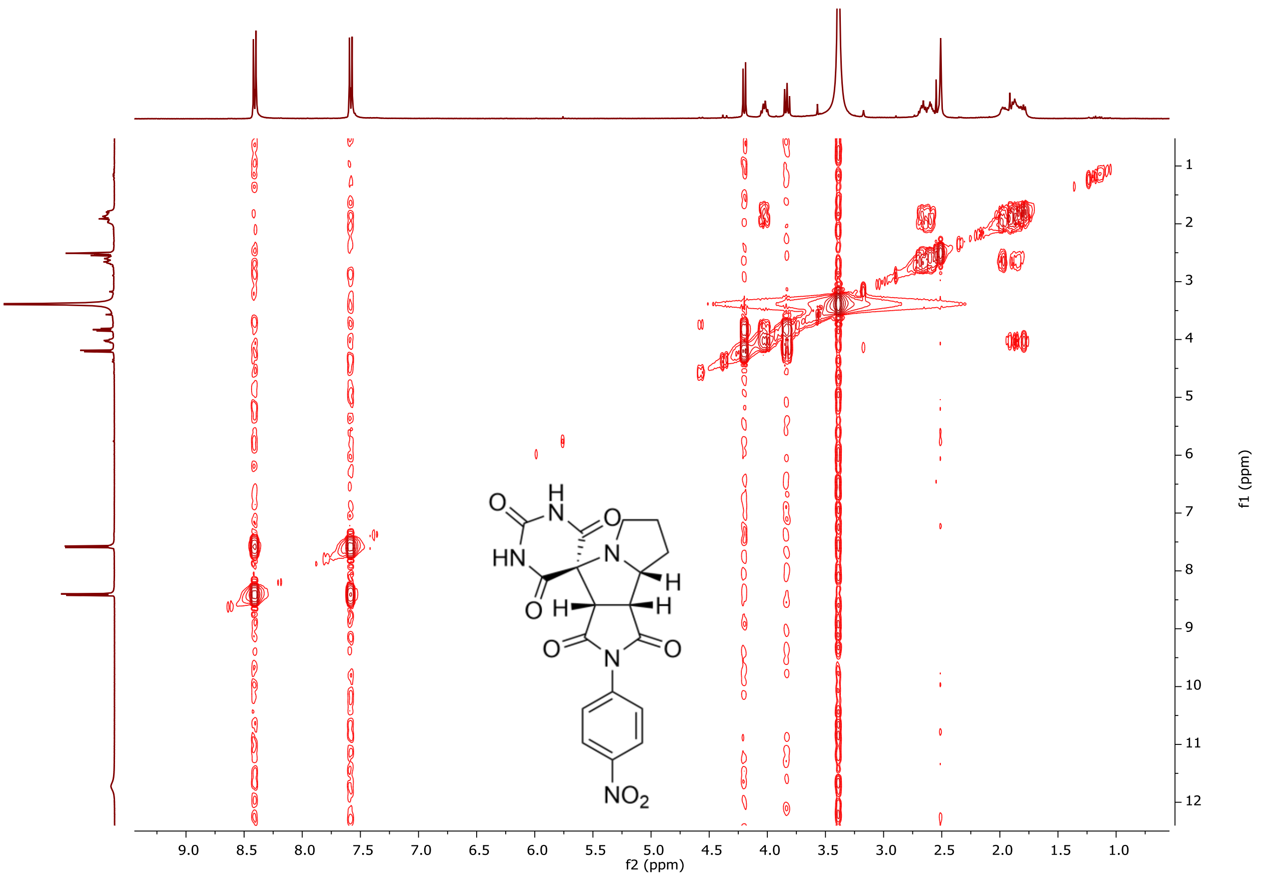

Figure S20. ^{1}H-^{1}H COSY NMR spectrum (DMSO-d_6) of **4e**

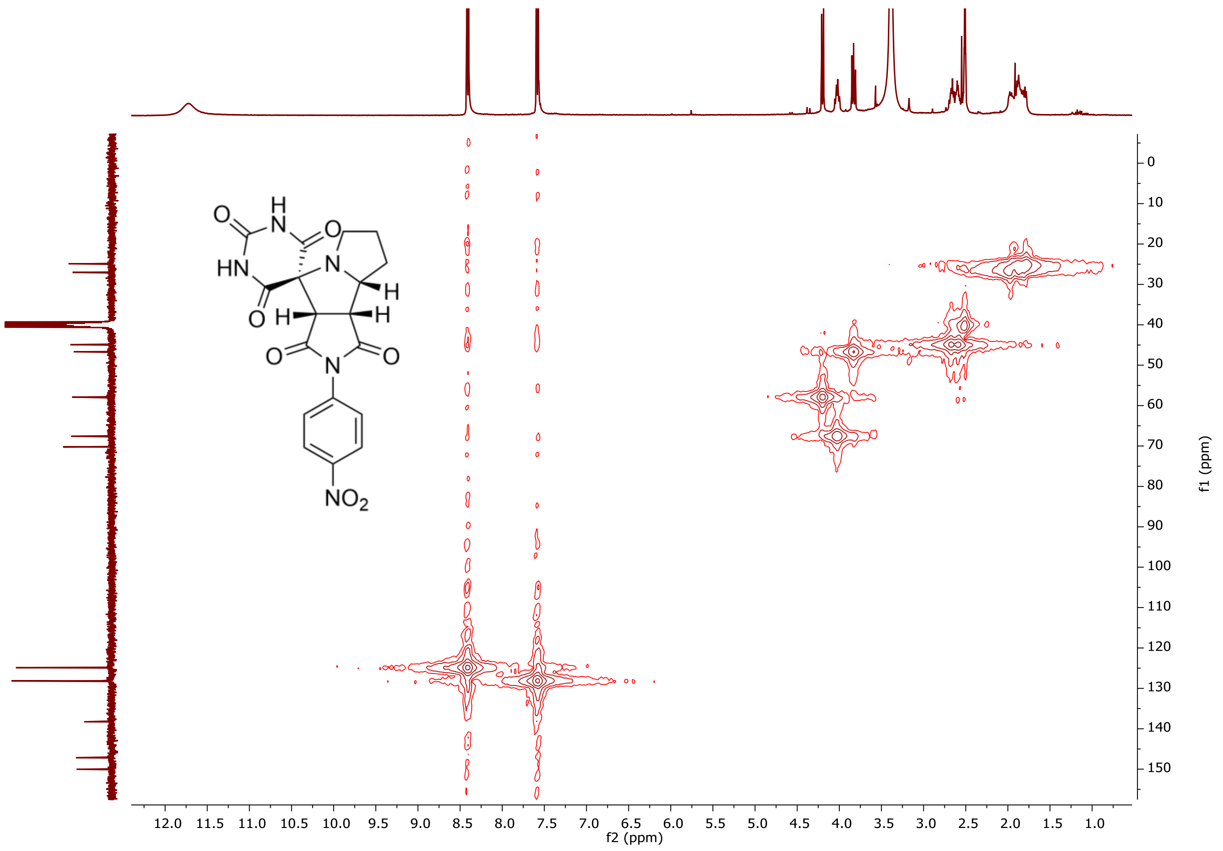

Figure S21. ^{1}H-^{13}C HSQC NMR spectrum (DMSO-d_6) of **4e**

(*rac*)-(*endo,exo*)-2'-Benzylhexahydro-1'*H*,2*H*-spiro[pyrimidine-5,4'-pyrrolo[3,4-*a*]pyrrolizine]-1',2,3',4,6(1*H*,2'*H*,3*H*)-pentaone (**4f**)

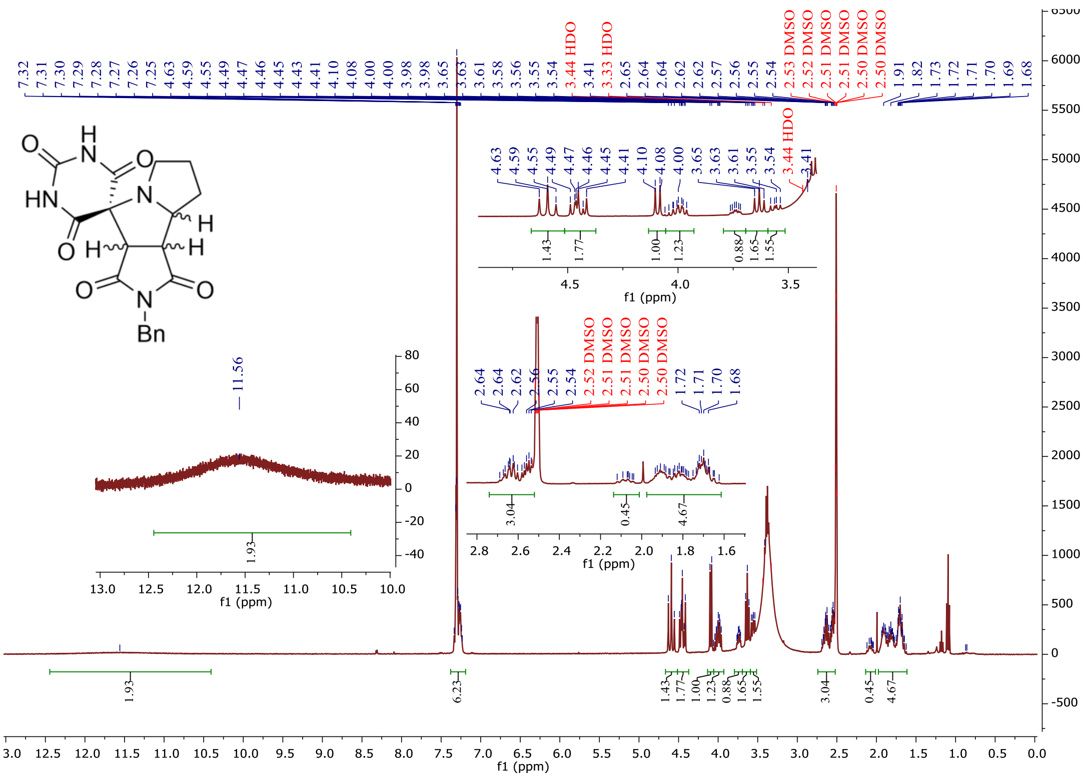

Figure S22. ^{1}H NMR spectrum (DMSO-d_6) of **4f**

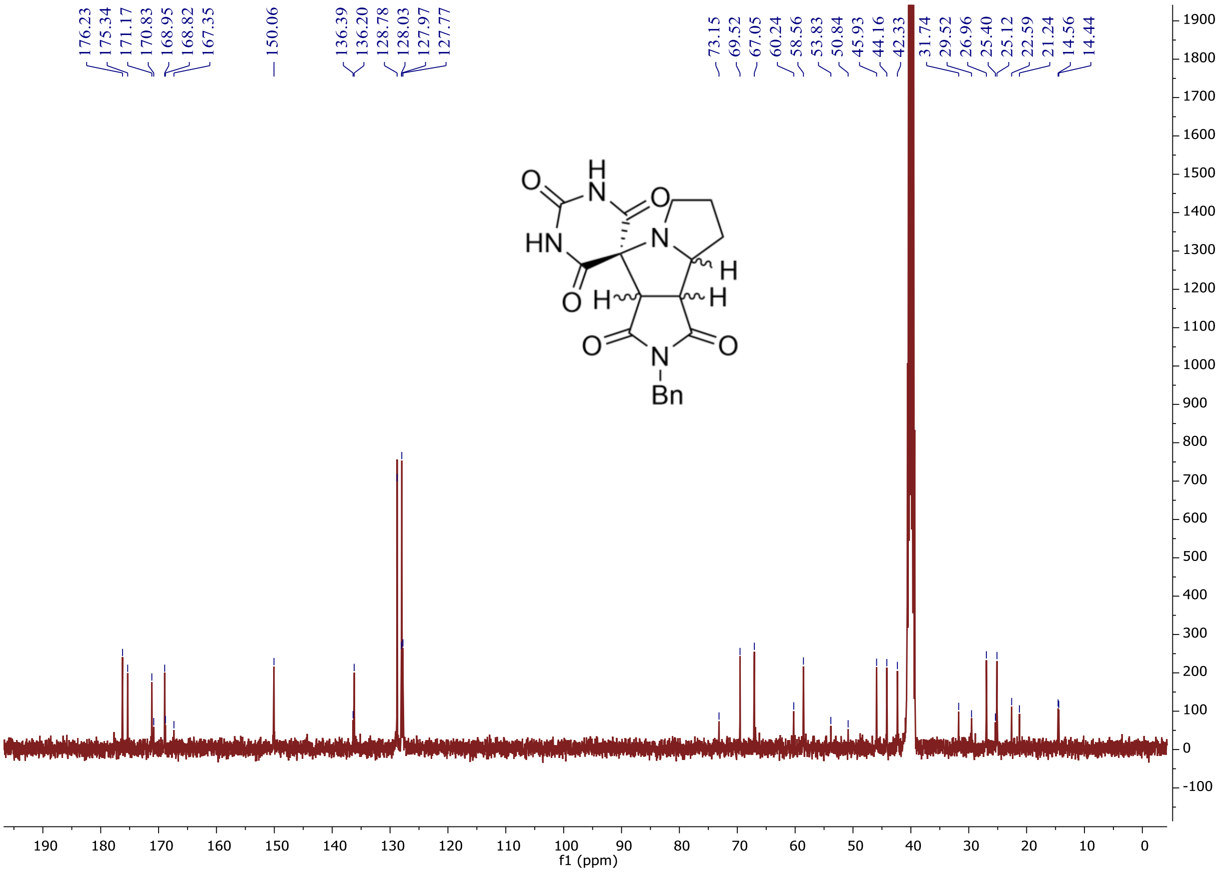

Figure S23. ^{13}C NMR spectrum (DMSO-d_6) of **4f**

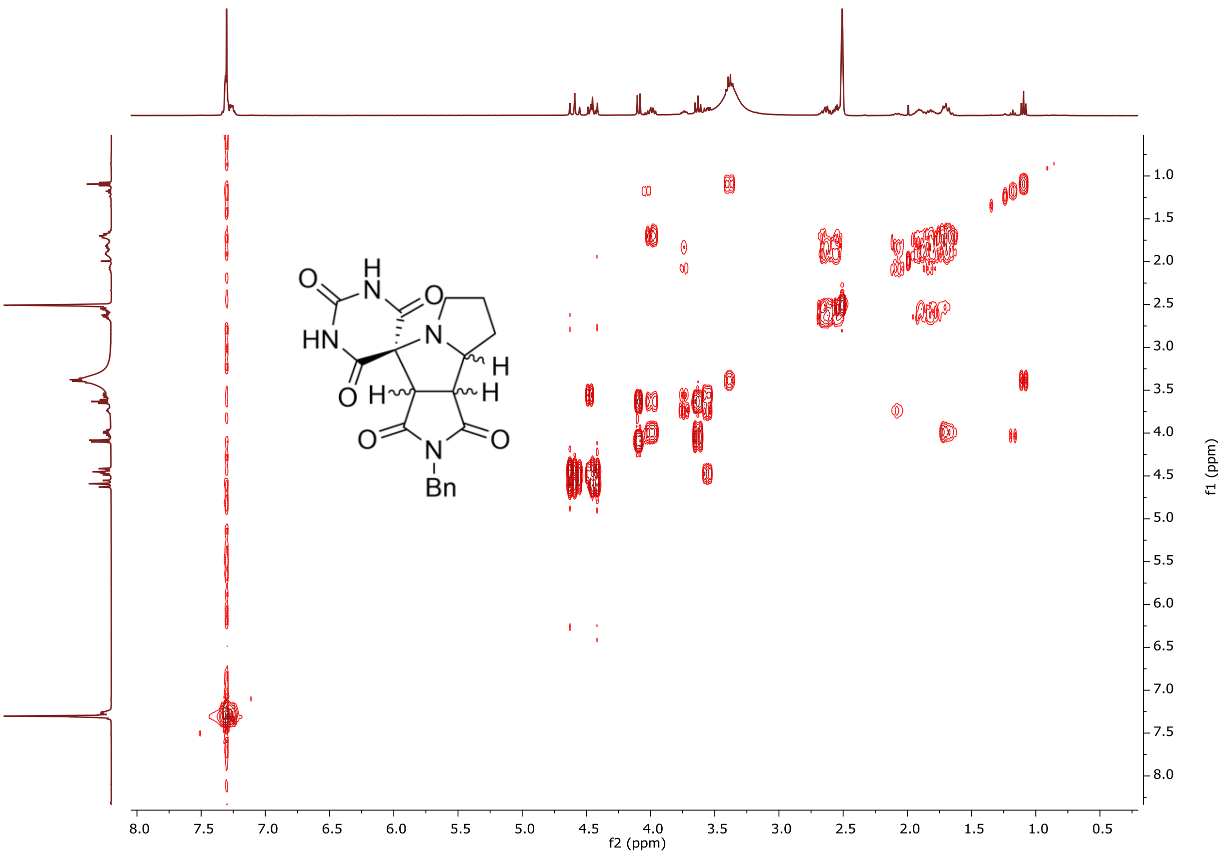

Figure S24. ^{1}H-^{1}H COSY NMR spectrum (DMSO-d_6) of **4f**

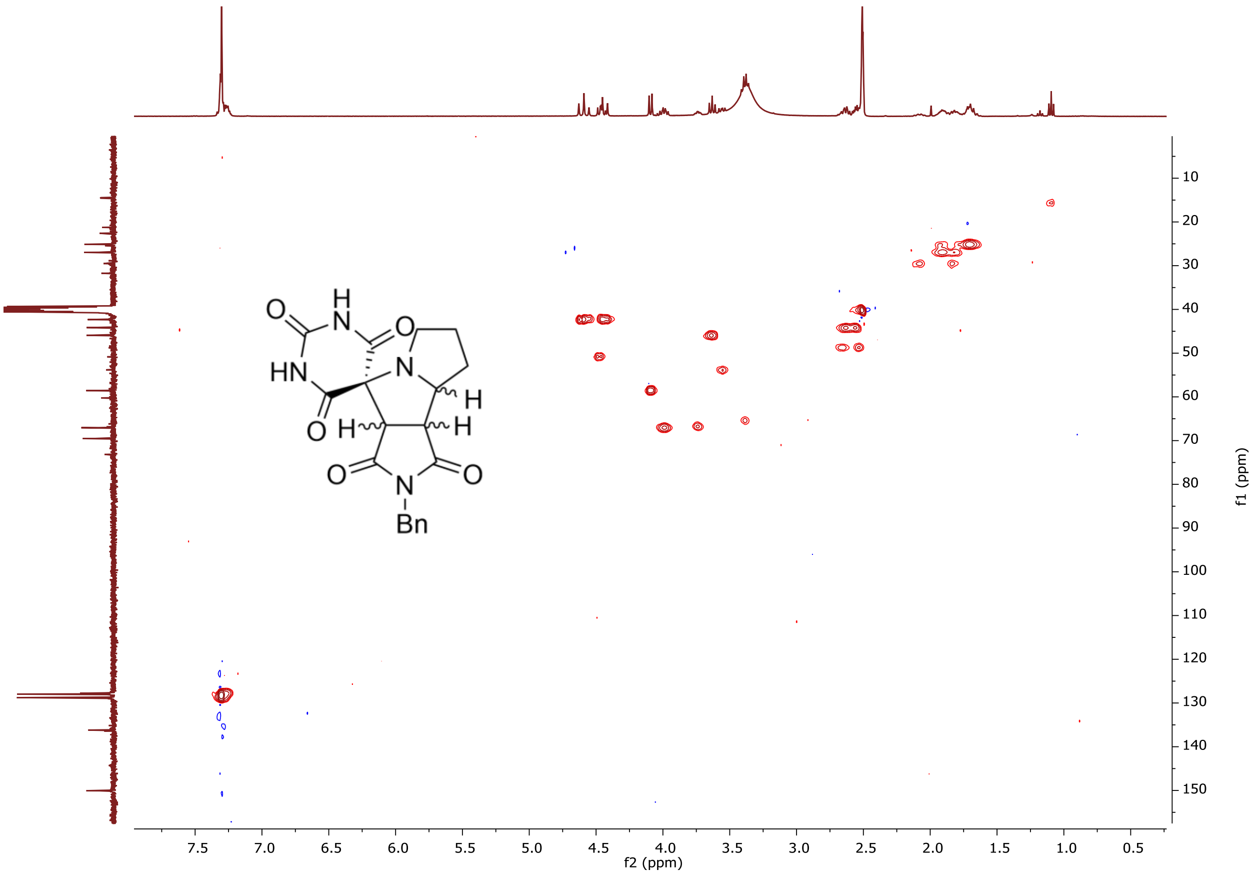

Figure S25. ^{1}H-^{13}C HSQC NMR spectrum (DMSO-d_6) of **4f**

(*rac*)-(*endo,exo*)-2'-Phenethylhexahydro-1'*H*,2*H*-spiro[pyrimidine-5,4'-pyrrolo[3,4-*a*]pyrrolizine]-1',2,3',4,6(1*H*,2'*H*,3*H*)-pentaone (**4g**)

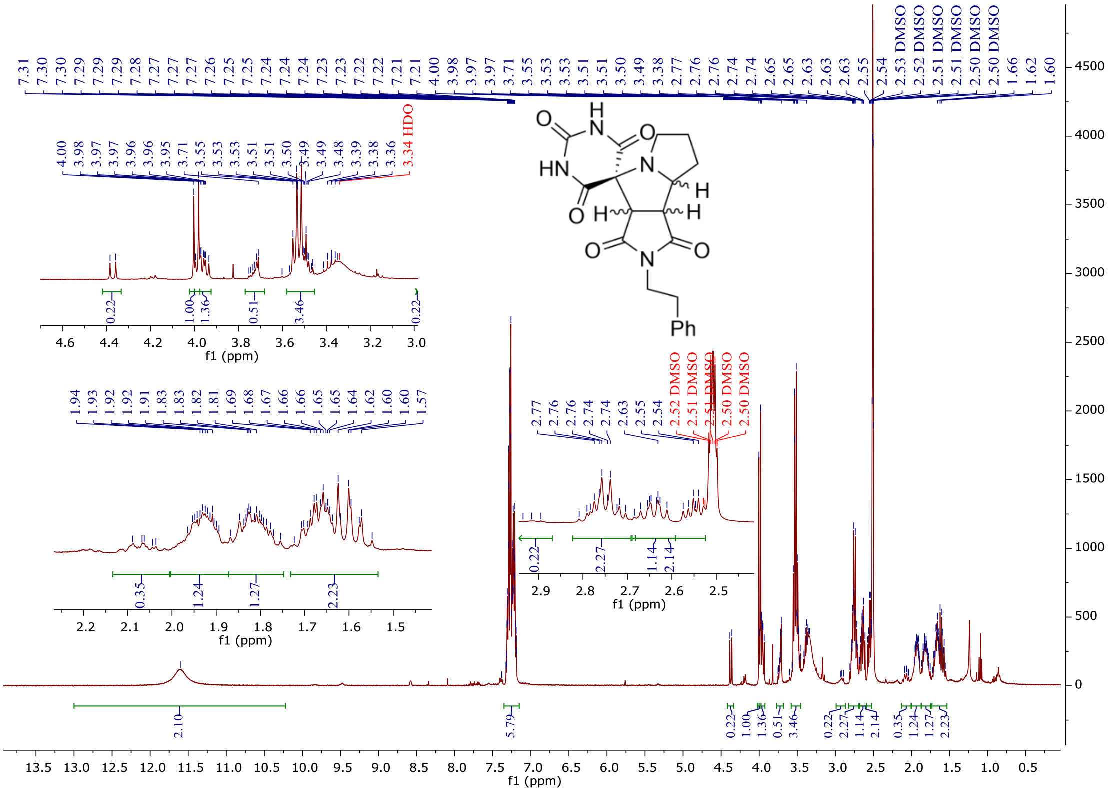

Figure S26. ^{1}H NMR spectrum (DMSO-d_6) of **4g**

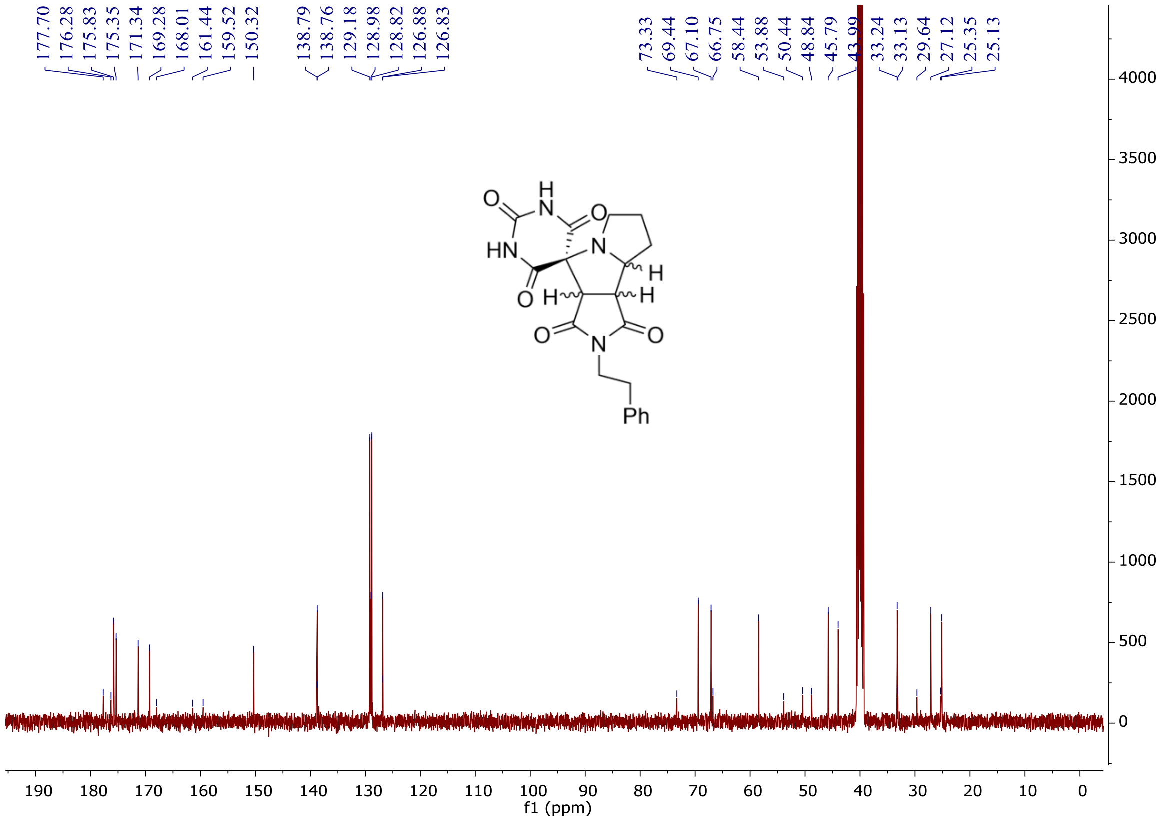

Figure S27. ^{13}C NMR spectrum (DMSO-d_6) of **4g**

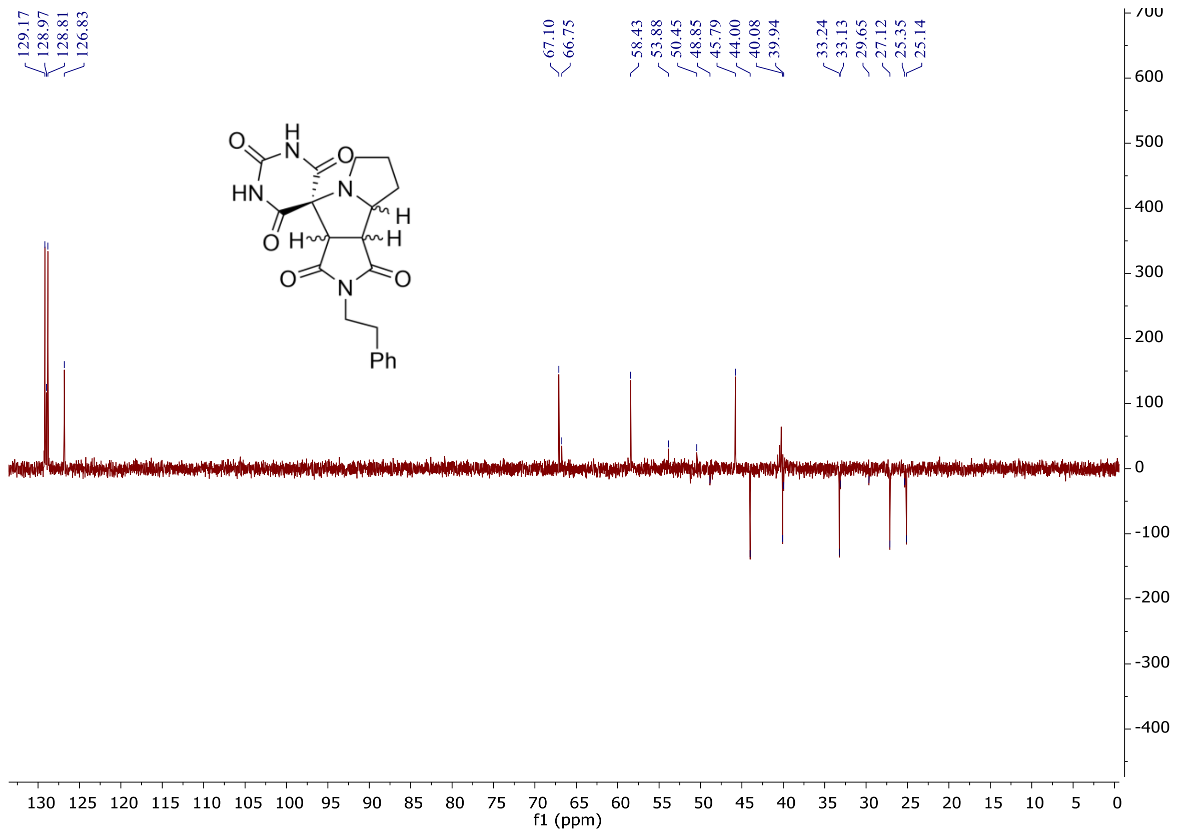

Figure S28. ^{13}C DEPT NMR spectrum (DMSO-d_6) of **4g**

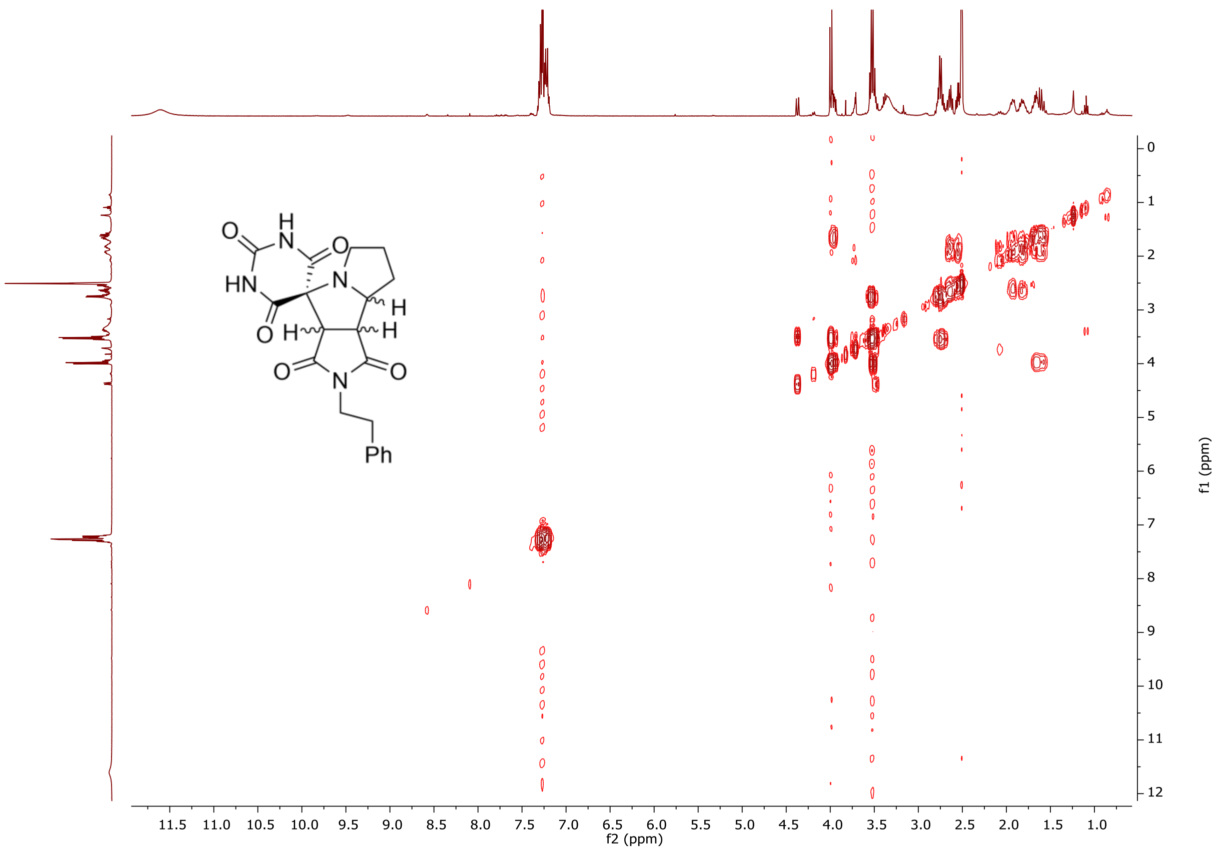

Figure S29. ^{1}H-^{1}H COSY NMR spectrum (DMSO-d_6) of **4g**

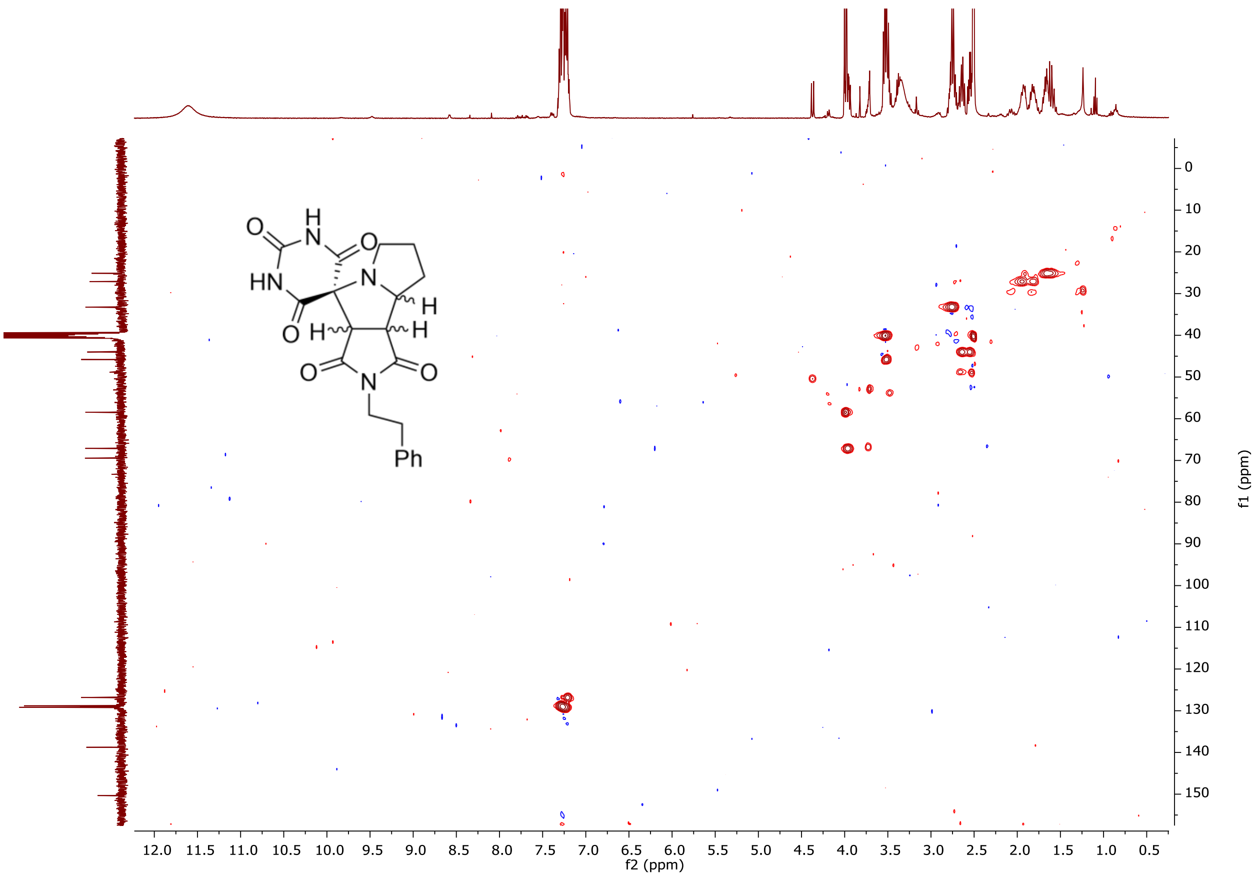

Figure S30. ^{1}H-^{13}C HSQC NMR spectrum (DMSO-d_6) of **4g**

(*rac*)-(*endo,exo*)-2'-(4-Chlorophenyl)hexahydro-1'*H*,2*H*-spiro[pyrimidine-5,4'-pyrrolo[3,3-*a*]pyrrolizine]-1',2,3',4,6(1*H*,2'*H*,3*H*)-pentaone (**4h**)

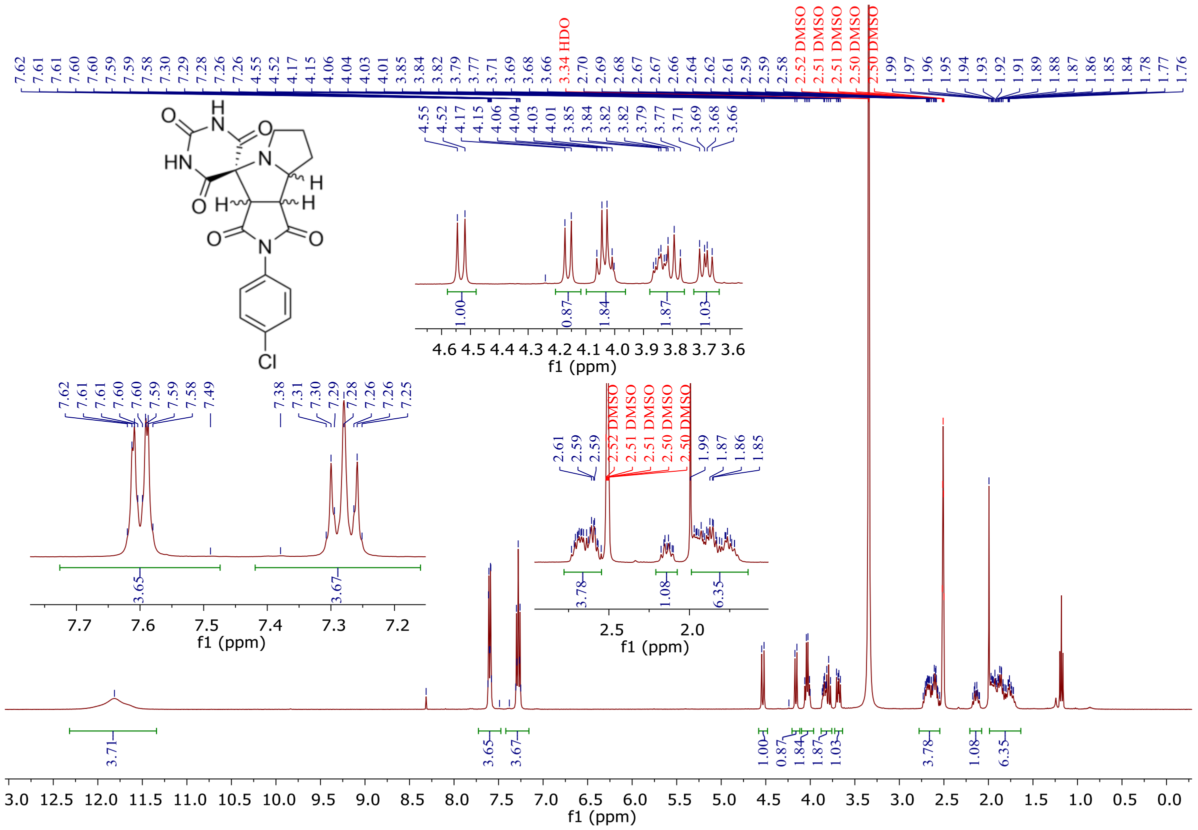

Figure S31. ^{1}H NMR spectrum (DMSO-d_6) of **4h**

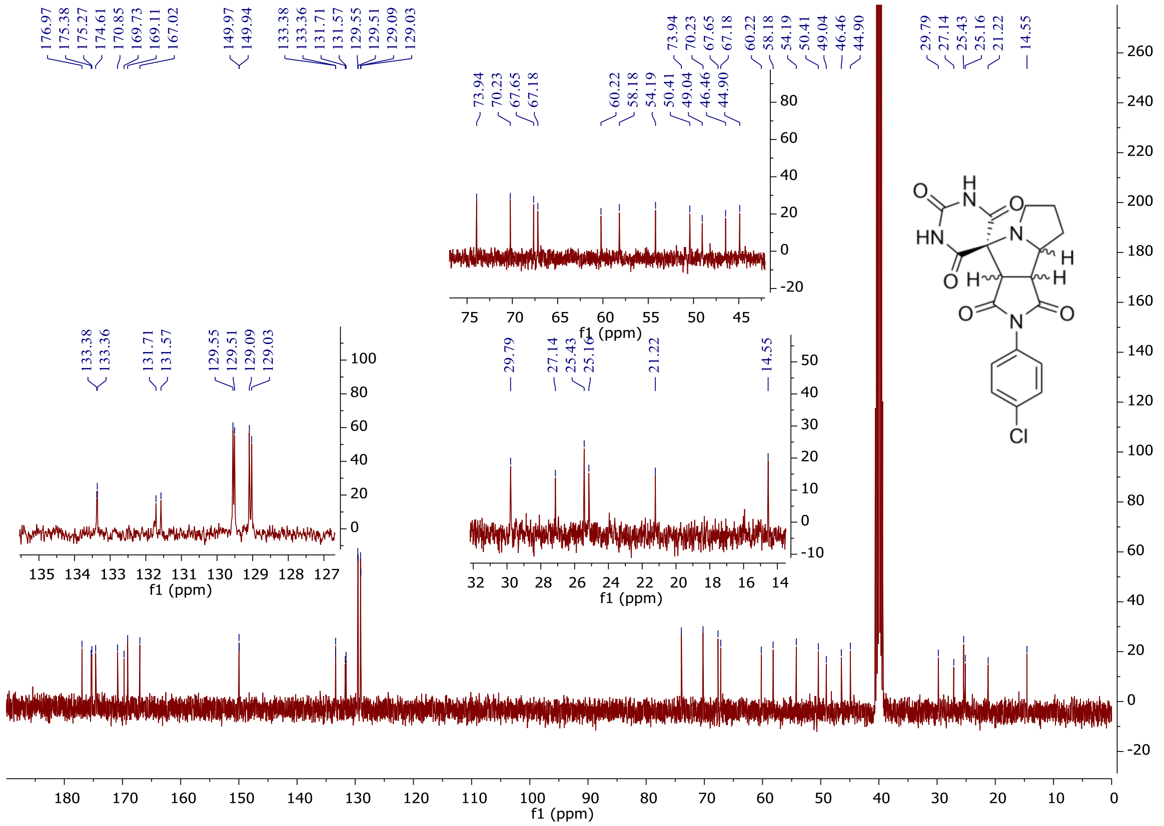

Figure S32. ^{13}C NMR spectrum (DMSO-d_6) of **4h**

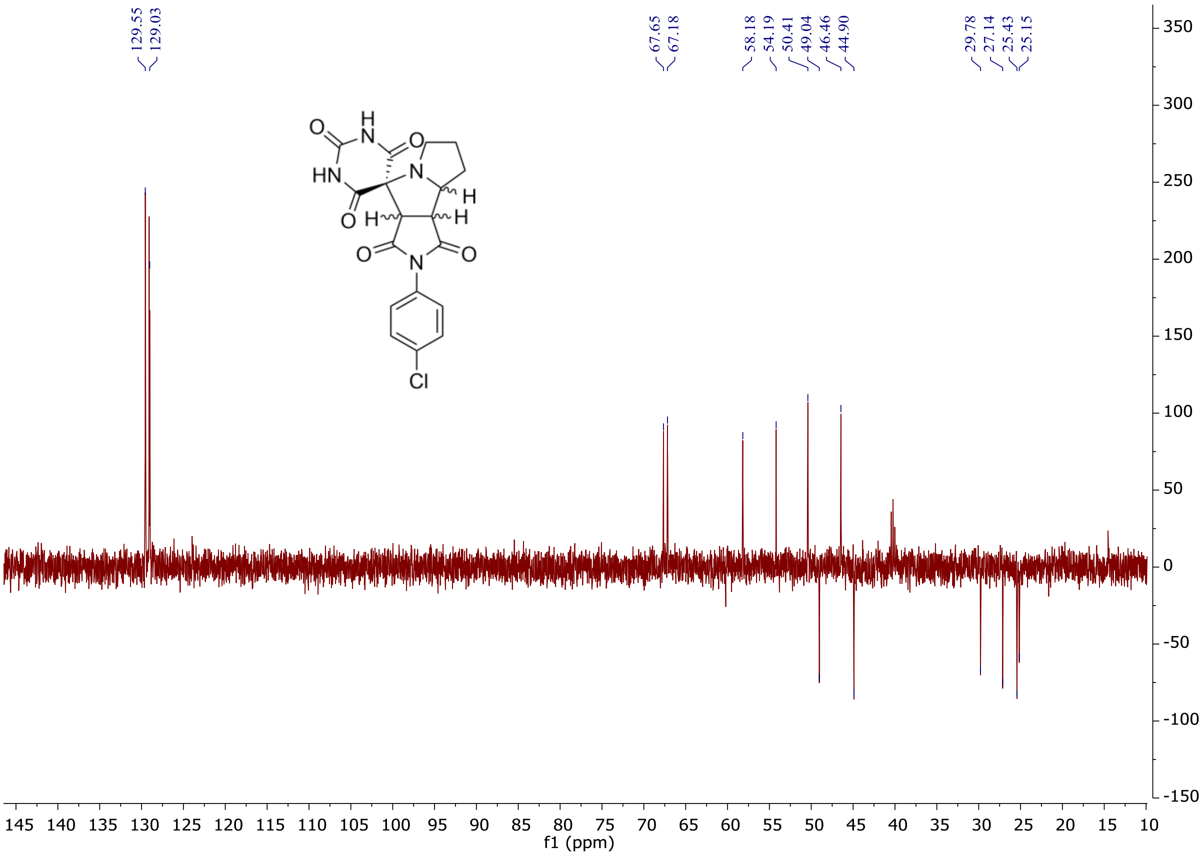

Figure S33. ^{13}C DEPT NMR spectrum (DMSO-d_6) of **4h**

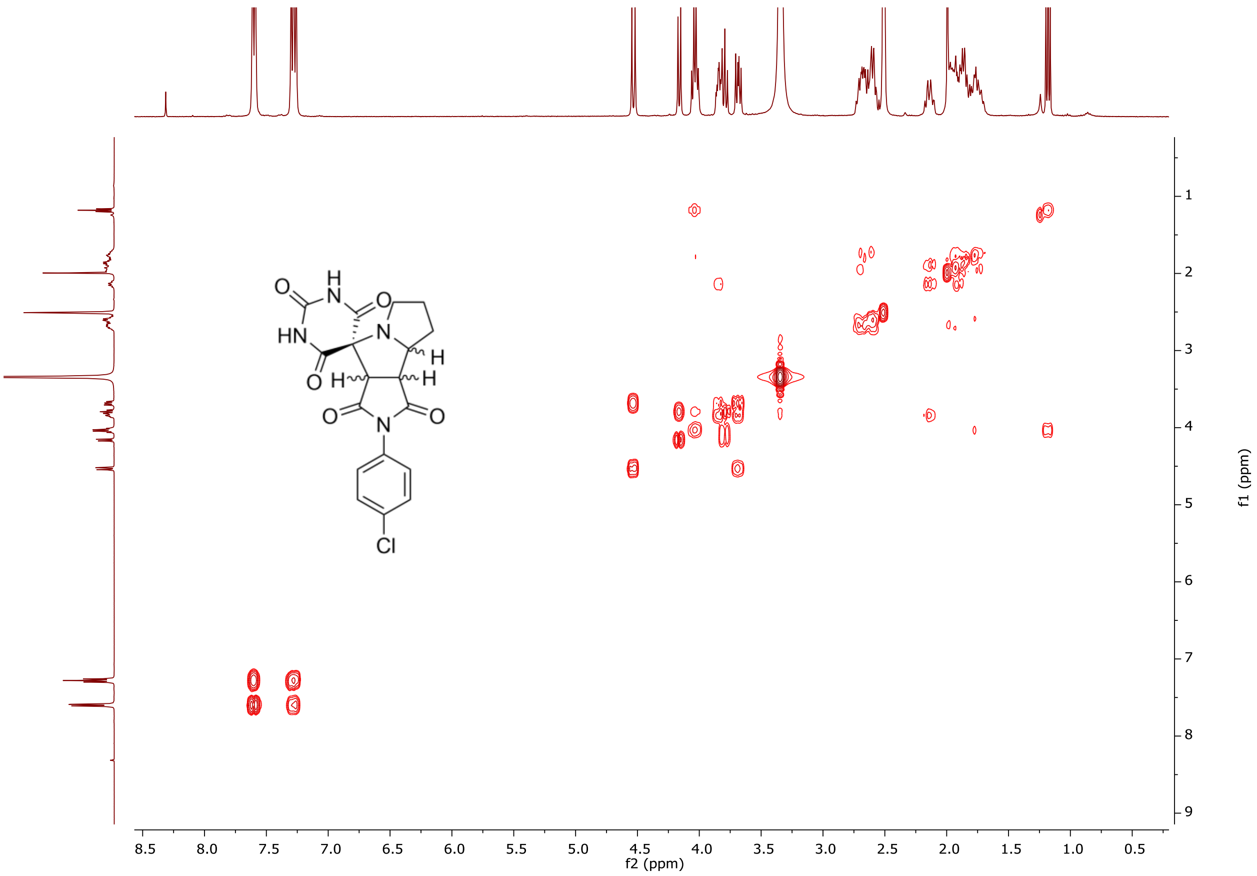

Figure S34. ^{1}H-^{1}H COSY NMR spectrum (DMSO-d_6) of **4h**

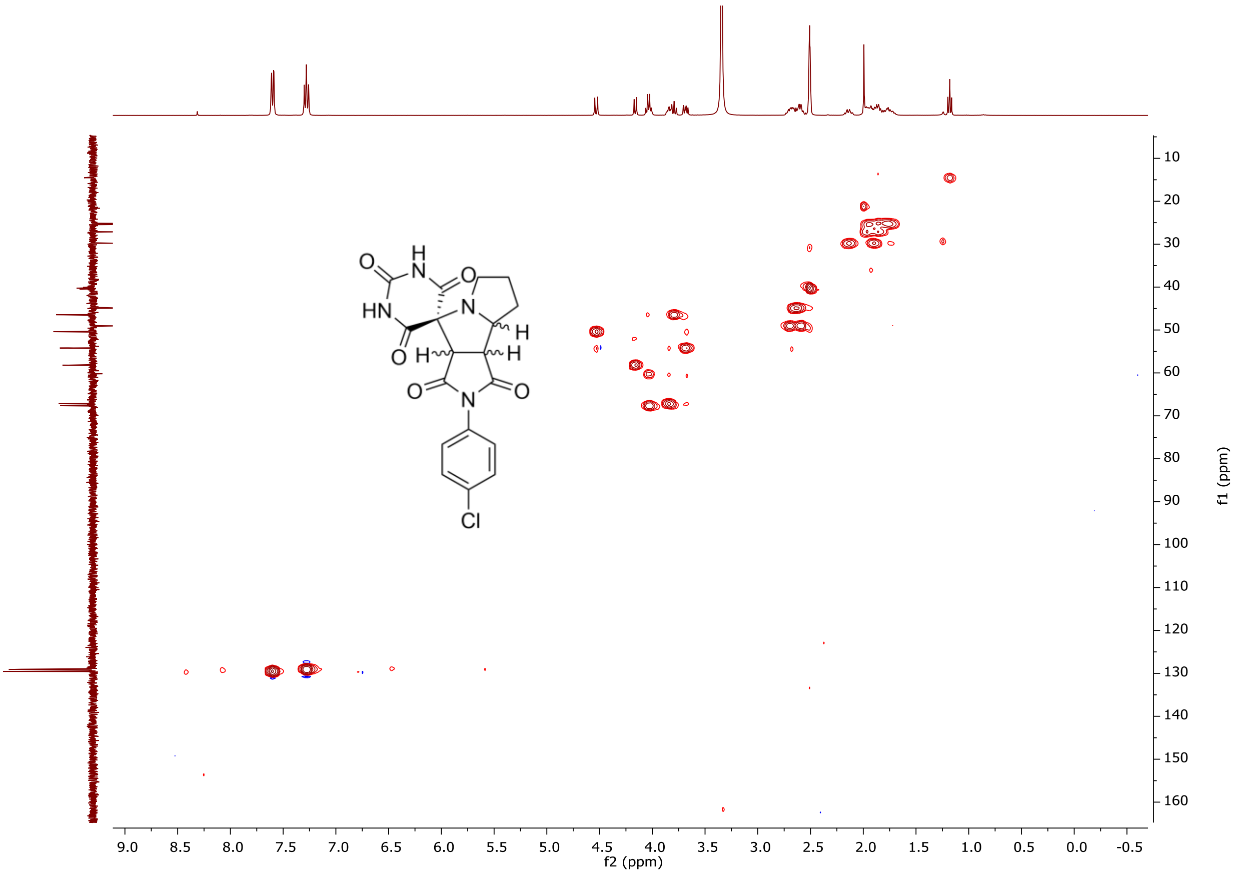

Figure S35. ^{1}H-^{13}C HSQC NMR spectrum (DMSO-d_6) of **4h**

(*rac*)-(*endo,exo*)-2'-(3,4-Dichlorophenyl)hexahydro-1'*H*,2*H*-spiro[pyrimidine-5,4'-pyrrolo[3,3-*a*]pyrrolizine]-1',2,3',4,6(1*H*,2'*H*,3*H*)-pentaone (**4i**)

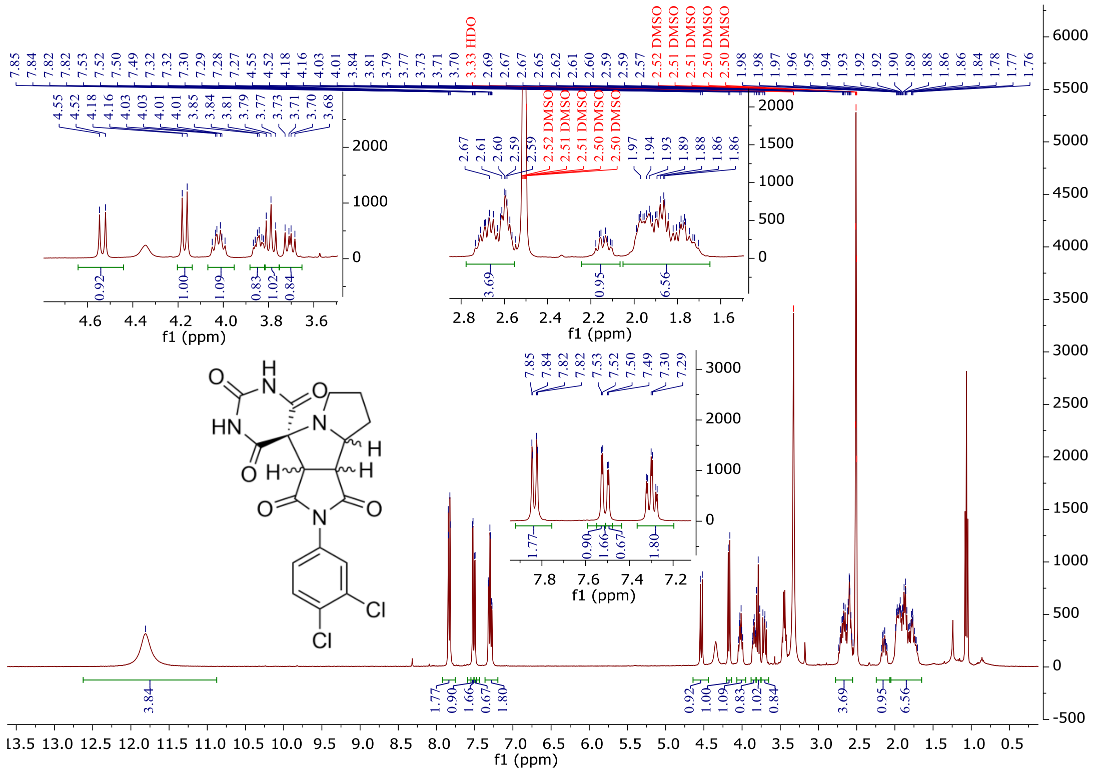

Figure S36. ^{1}H NMR spectrum (DMSO-d_6) of **4i**

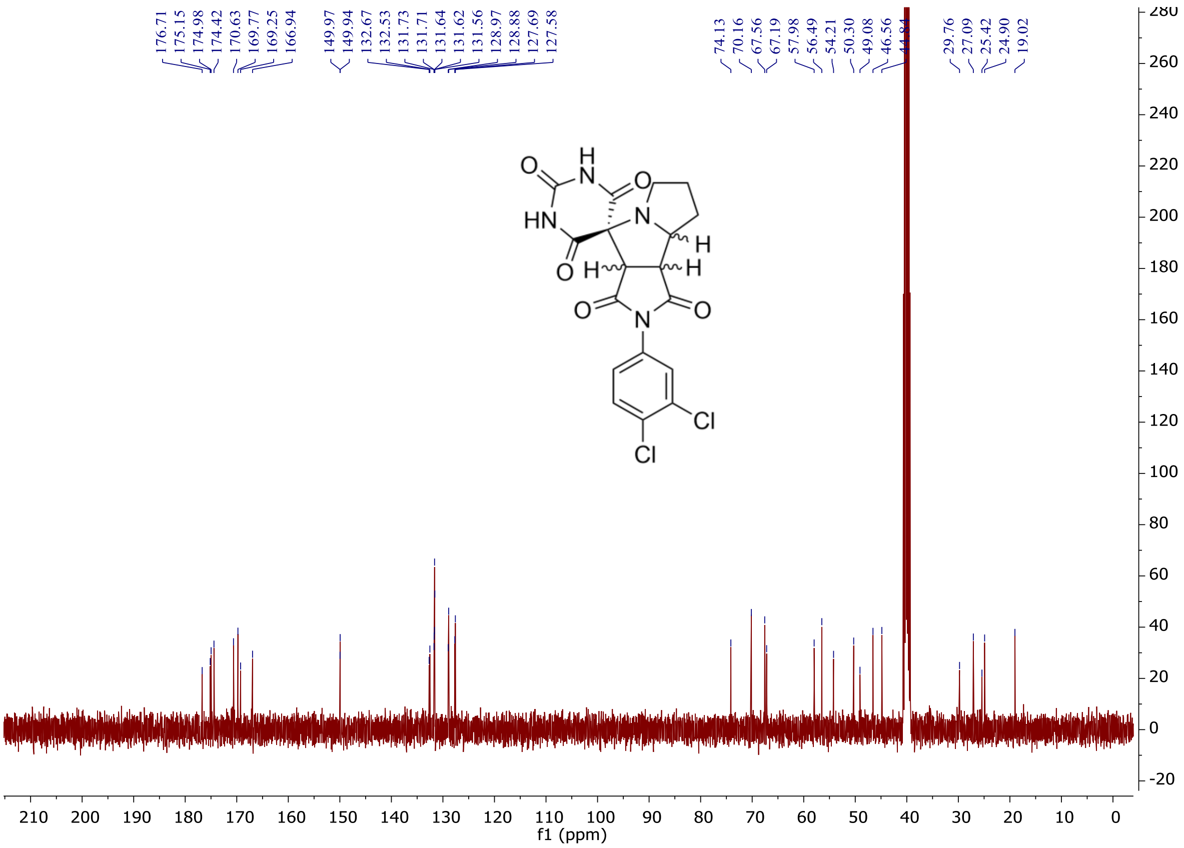

Figure S37. ^{13}C NMR spectrum (DMSO-d_6) of **4i**

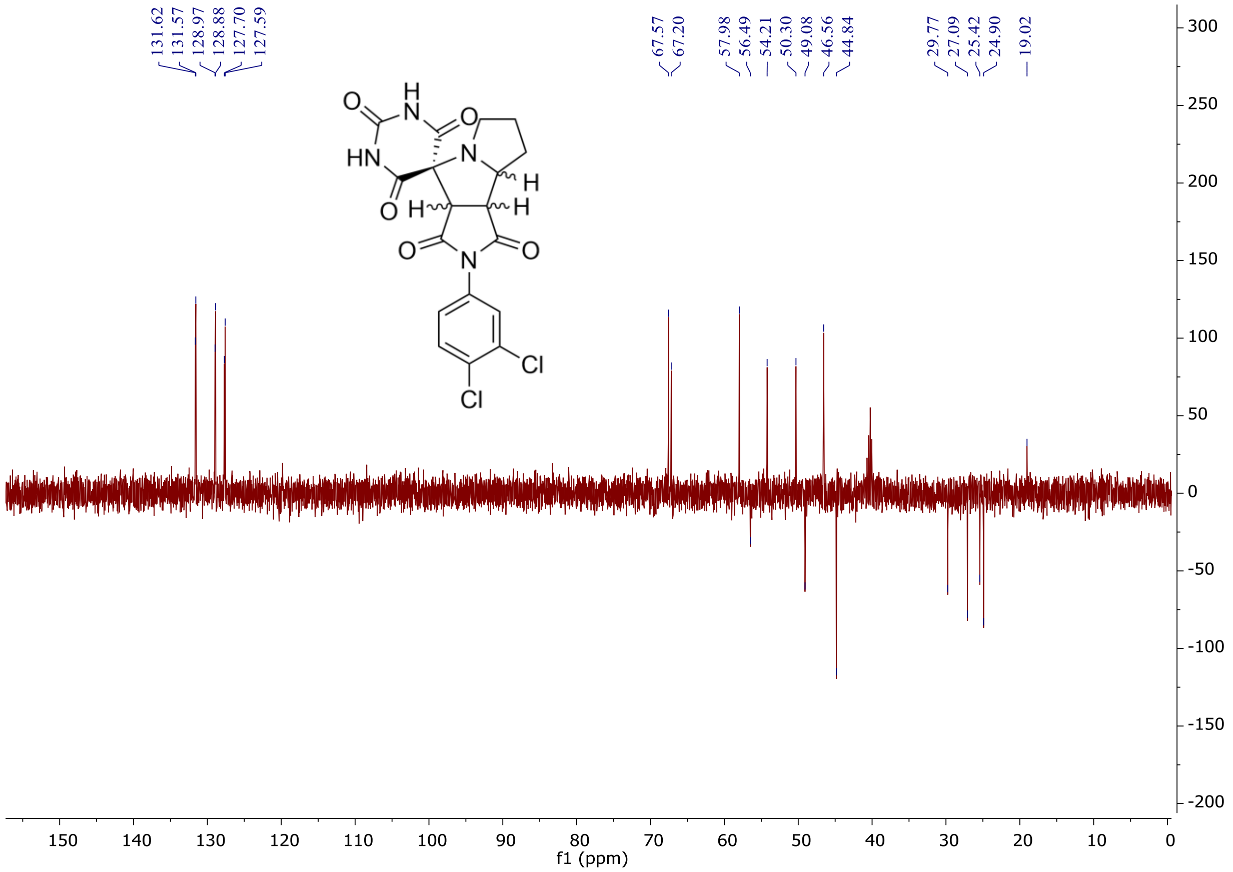

Figure S38. ^{13}C DEPT NMR spectrum (DMSO-d_6) of **4i**

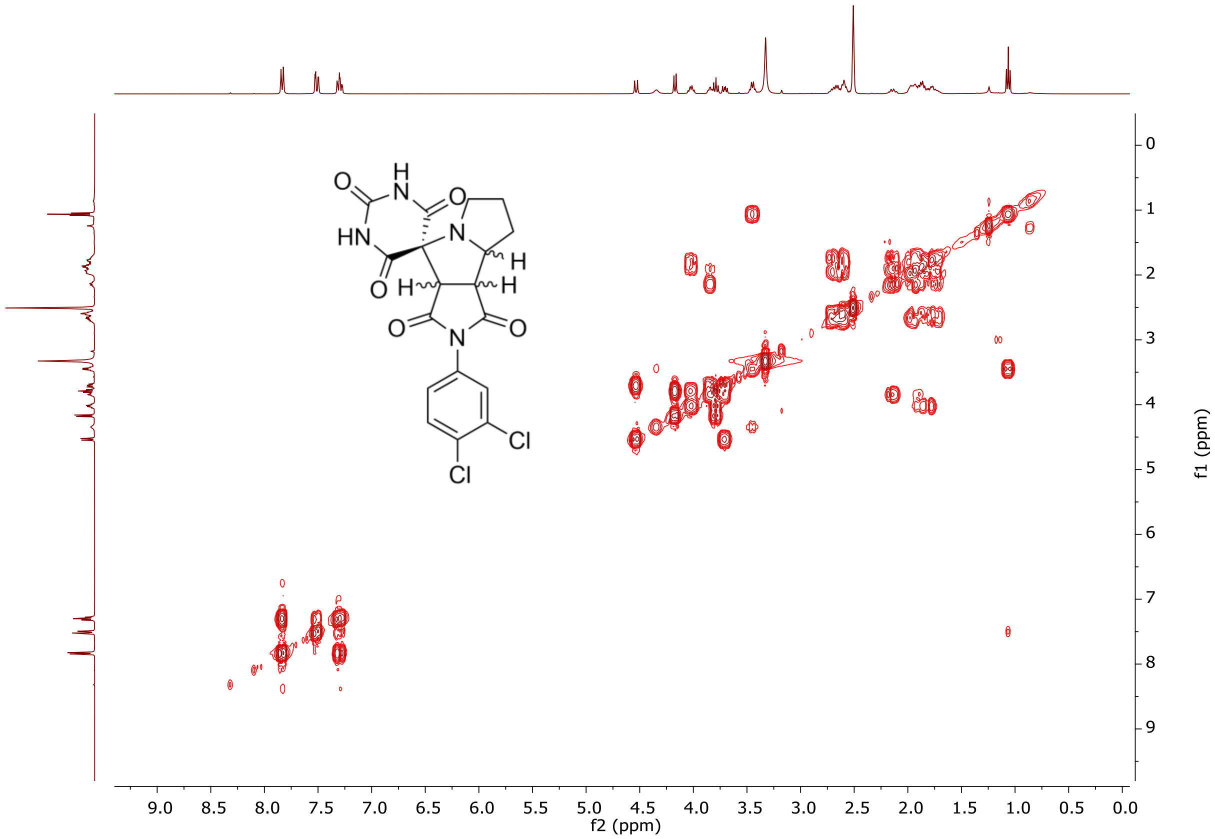

Figure S39. ^{1}H-^{1}H COSY NMR spectrum (DMSO-d_6) of **4i**

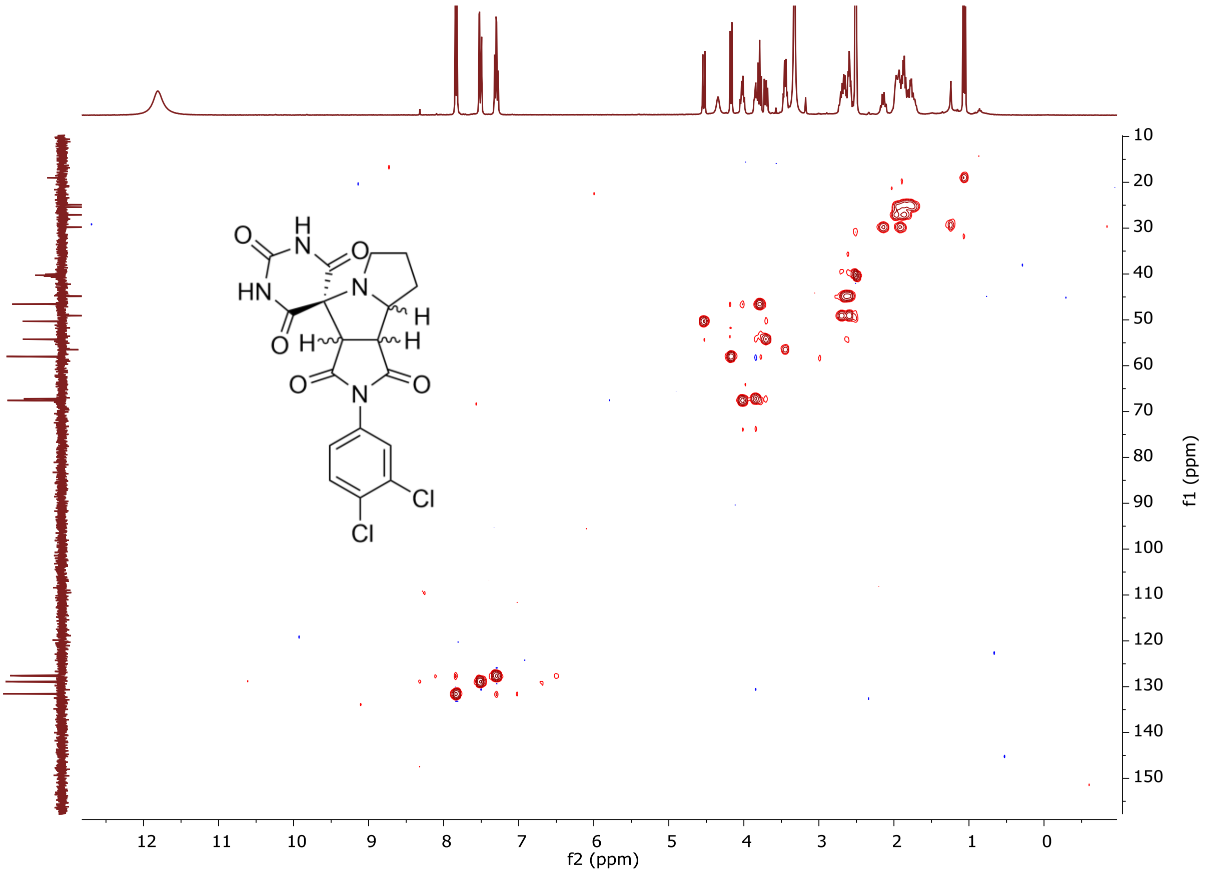

Figure S40. ^{1}H-^{13}C HSQC NMR spectrum (DMSO-d_6) of **4i**

(*rac*)-(*endo,exo*)-2'-(4-Chloro-3-fluorophenyl)hexahydro-1'*H*,2*H*-spiro[pyrimidine-5,4'-pyrrolo[3,3-*a*]pyrrolizine]-1',2,3',4,6(1*H*,2'*H*,3*H*)-pentaone (**4j**)

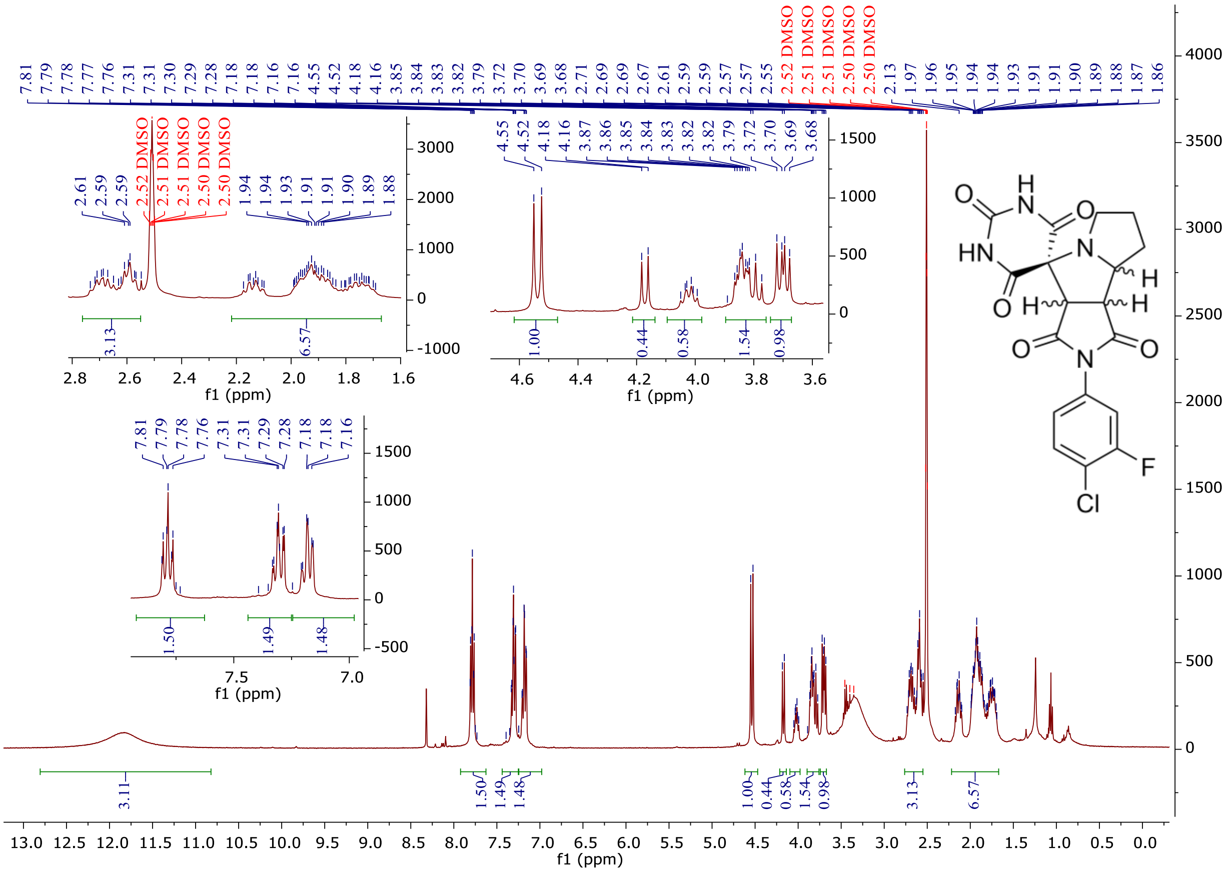

Figure S41. ^{1}H NMR spectrum (DMSO-d_6) of **4j**

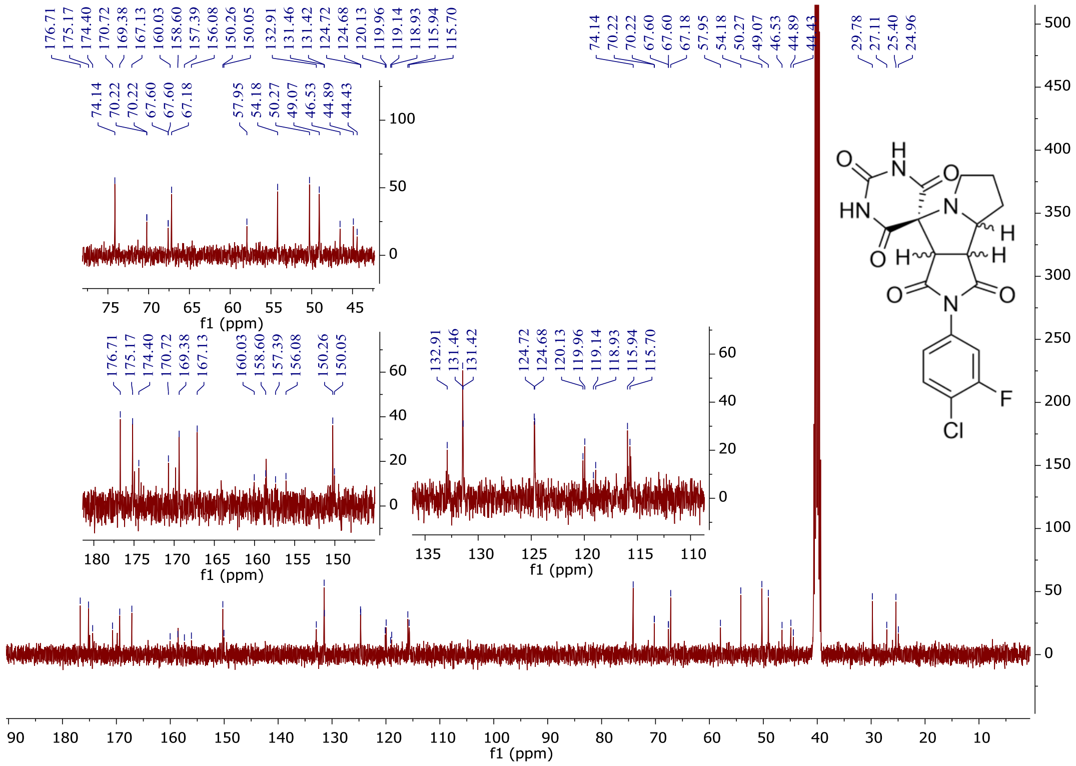

Figure S42. ^{13}C NMR spectrum (DMSO-d_6) of **4j**

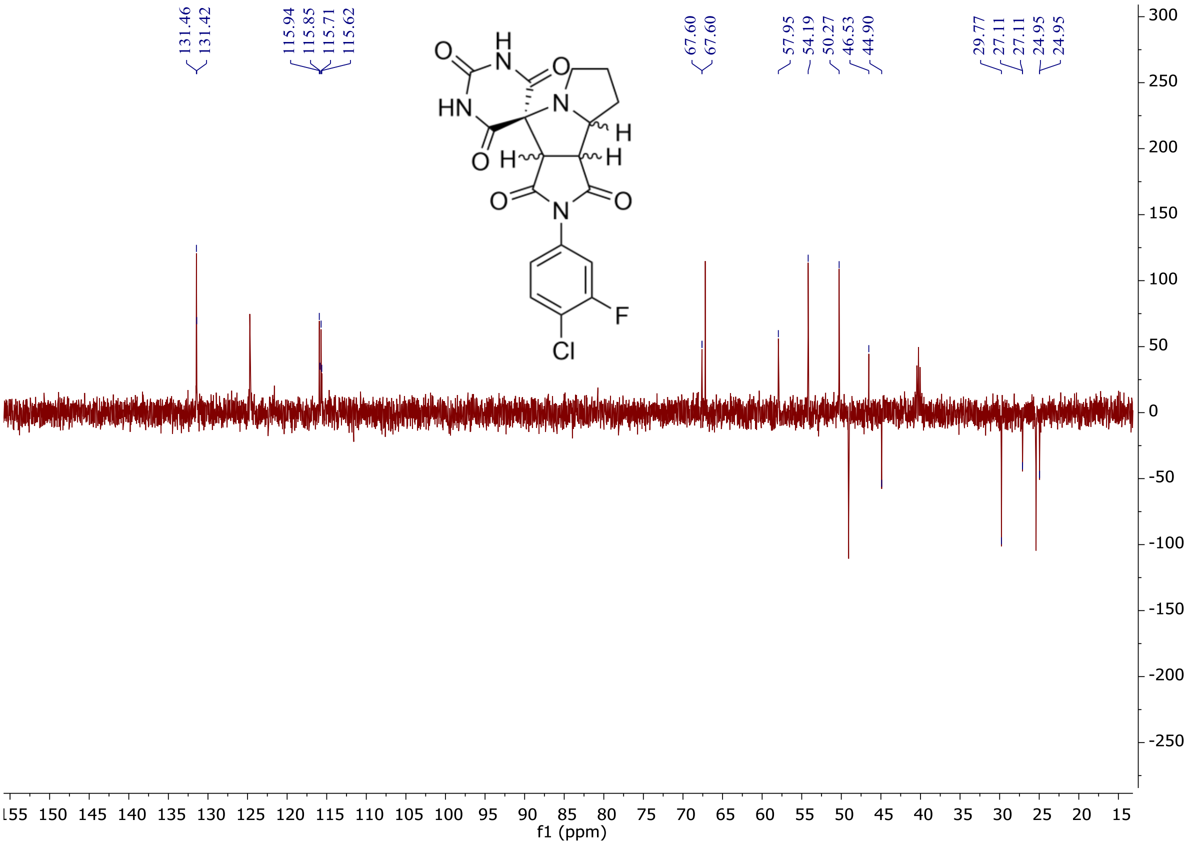

Figure S43. ^{13}C DEPT NMR spectrum (DMSO-d_6) of **4j**

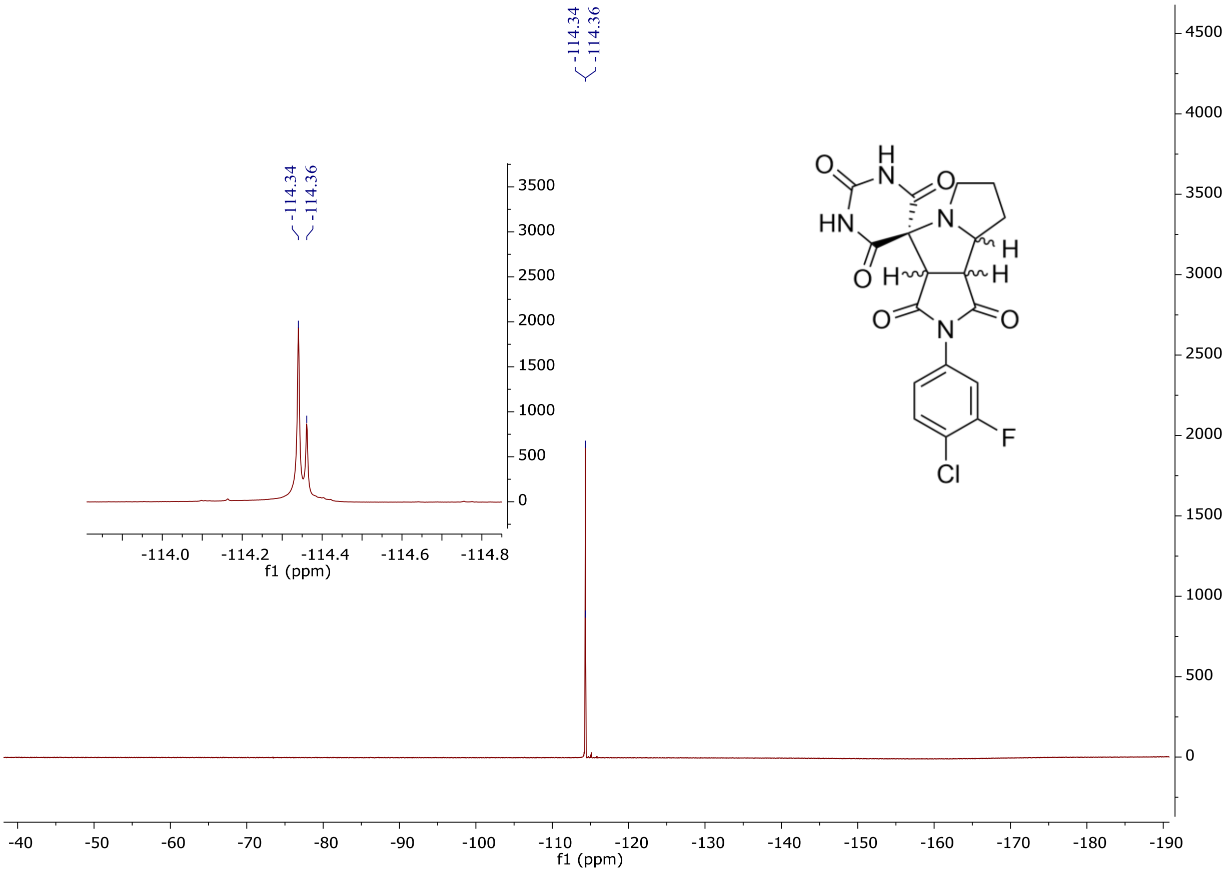

Figure S44. ^{19}F NMR spectrum (DMSO-d_6) of **4j**

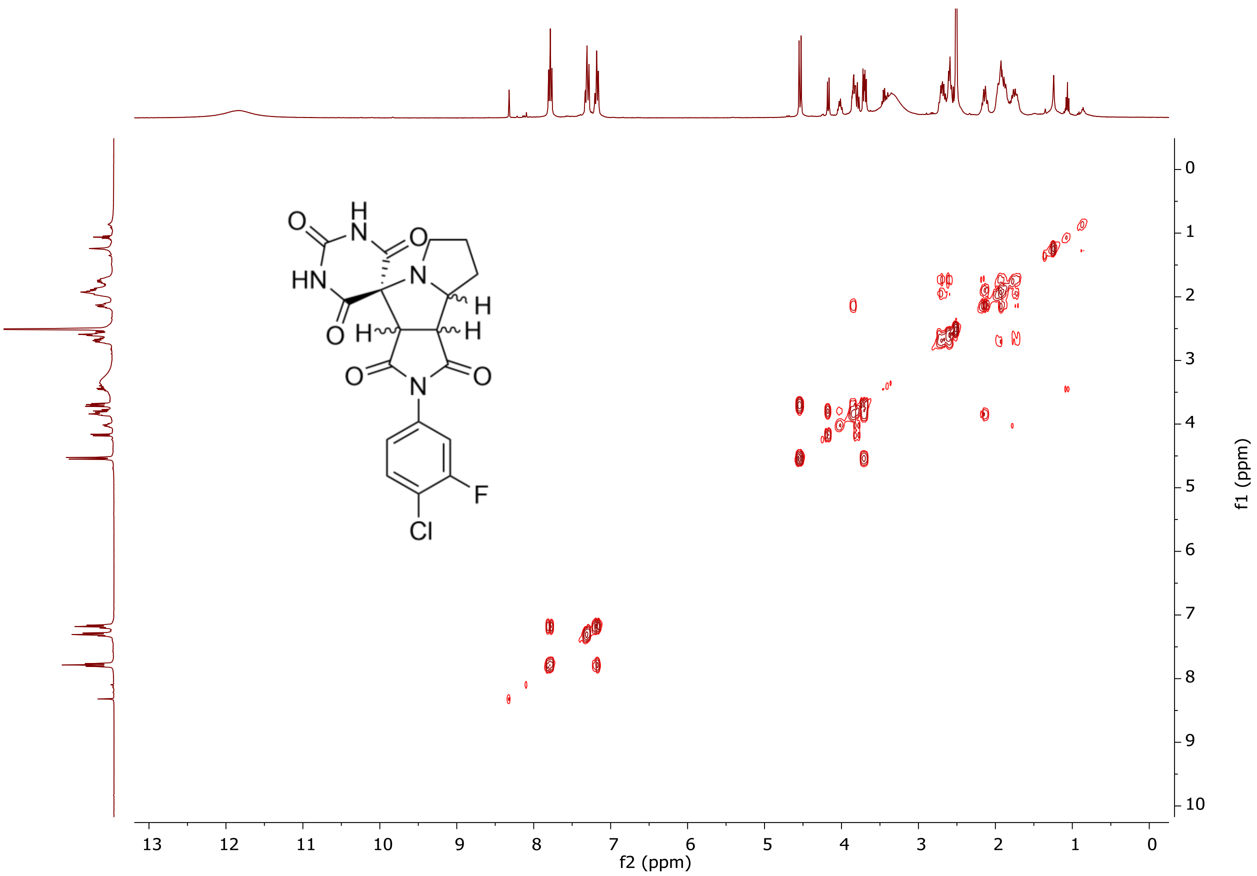

Figure S45. ^{1}H-^{1}H COSY NMR spectrum (DMSO-d_6) of **4j**

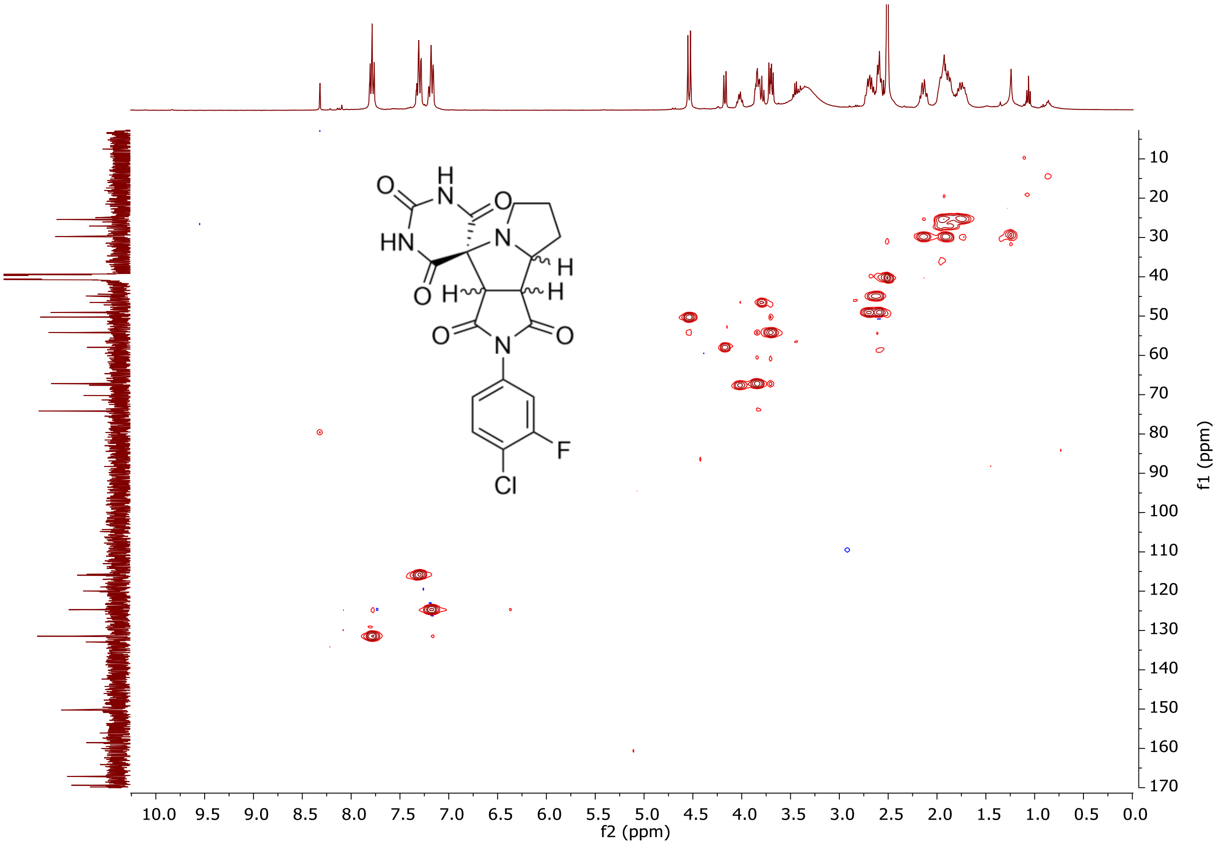

Figure S46. ^{1}H-^{13}C HSQC NMR spectrum (DMSO-d_6) of **4j**

(*rac*)-(*endo,exo*)-2'-(3-Nitrophenyl)hexahydro-1'*H*,2*H*-spiro[pyrimidine-5,4'-pyrrolo[3,4-*a*]pyrrolizine]-1',2,3',4,6(1*H*,2'*H*,3*H*)-pentaone (**4k**)

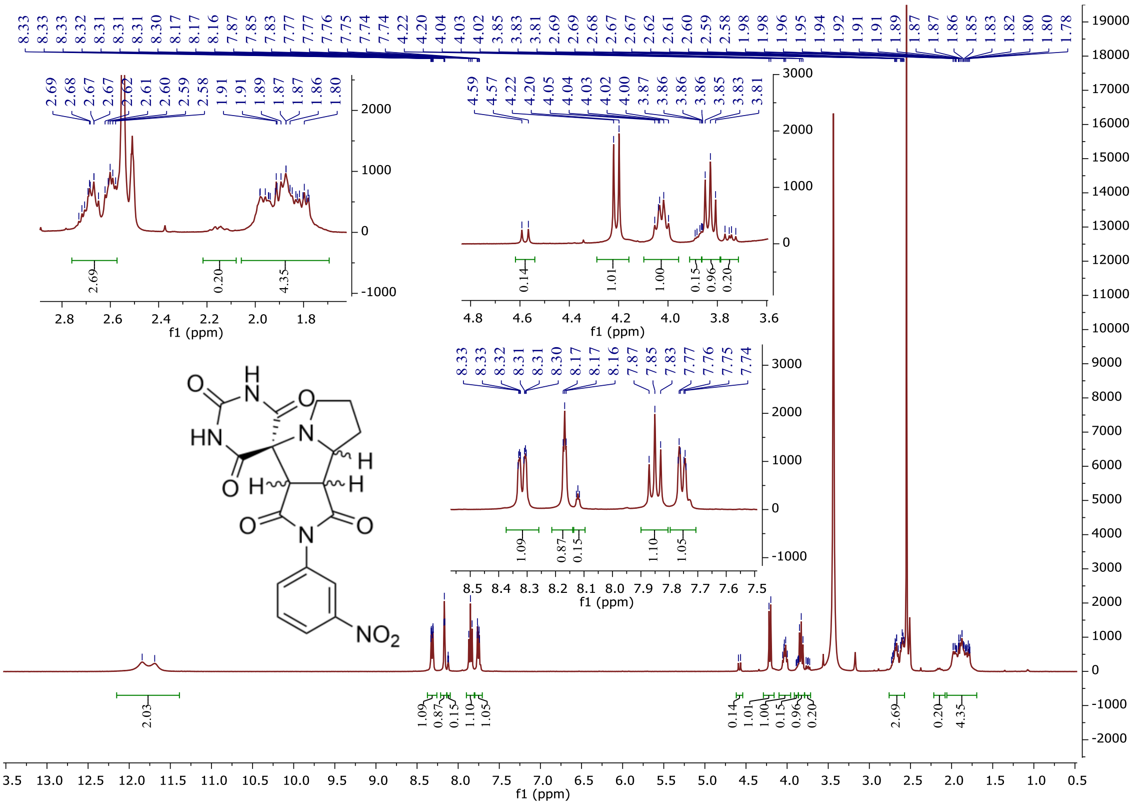

Figure S47. ^{1}H NMR spectrum (DMSO-d_6) of **4k**

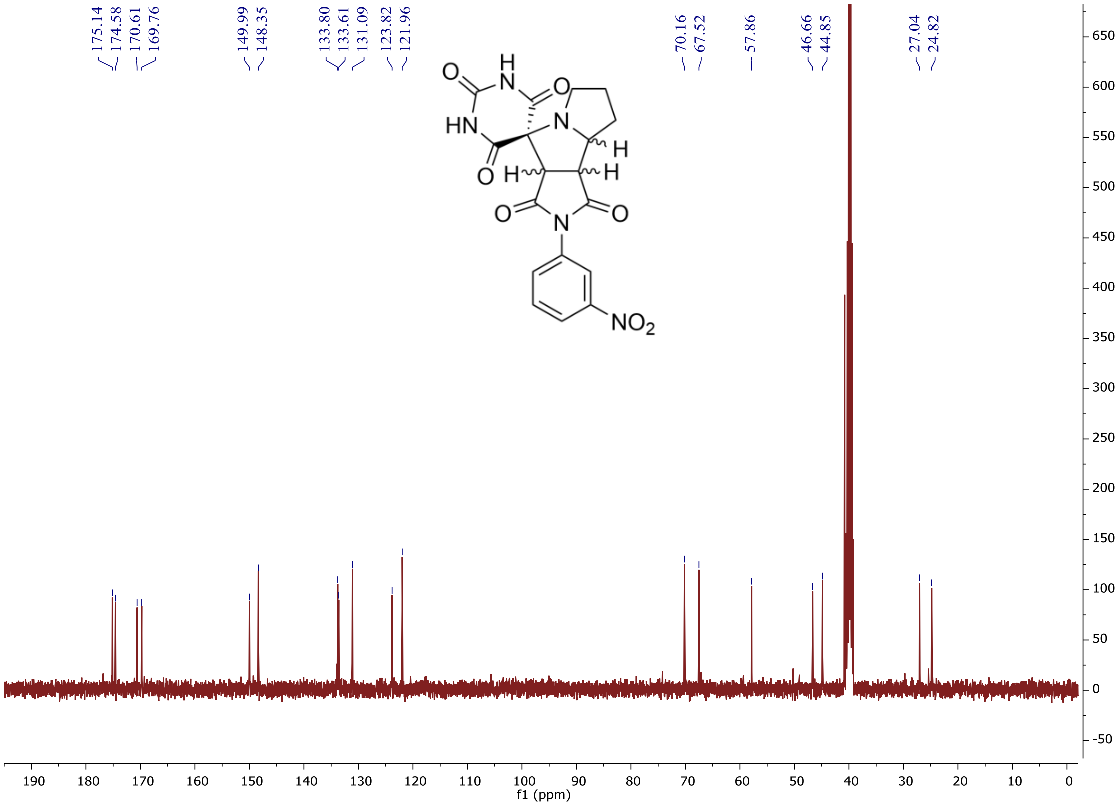

Figure S48. ^{13}C NMR spectrum (DMSO-d_6) of **4k**

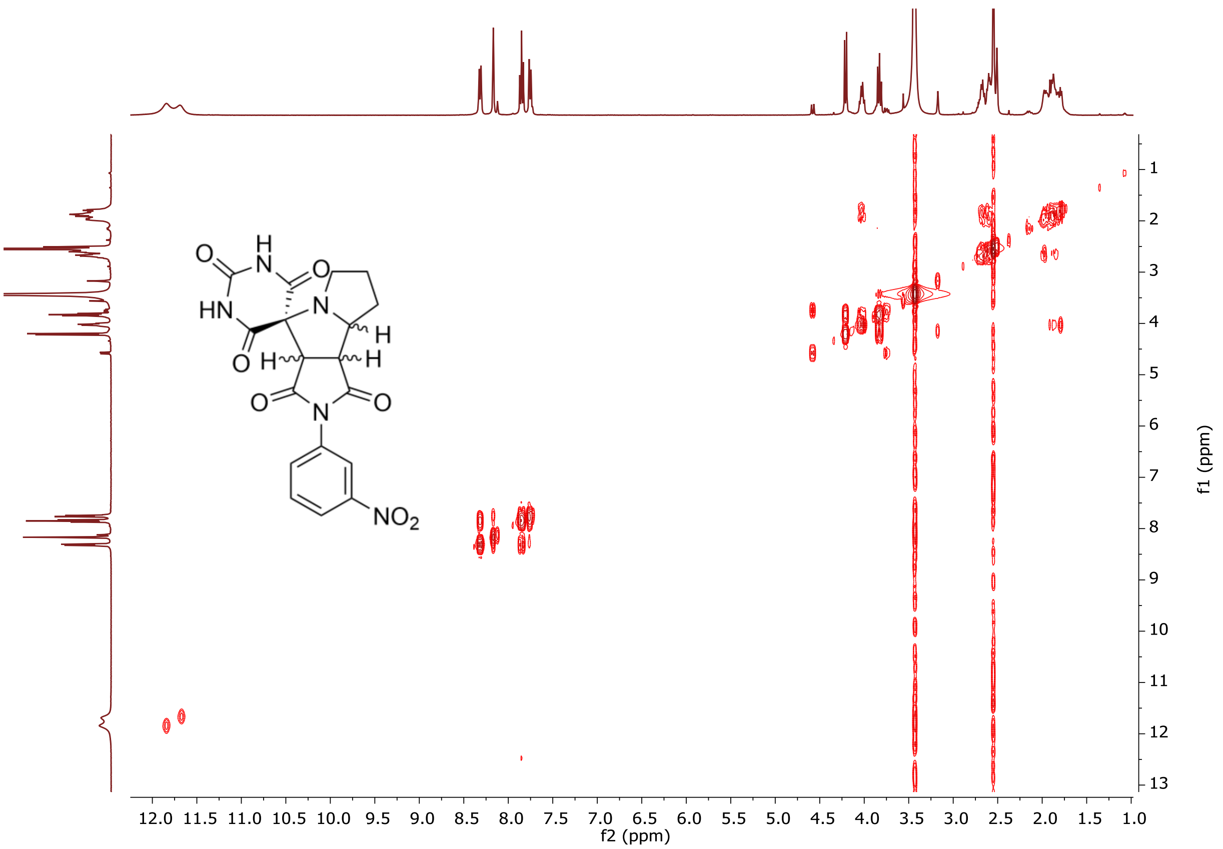

Figure S49. ^{1}H-^{1}H COSY NMR spectrum (DMSO-d_6) of **4k**

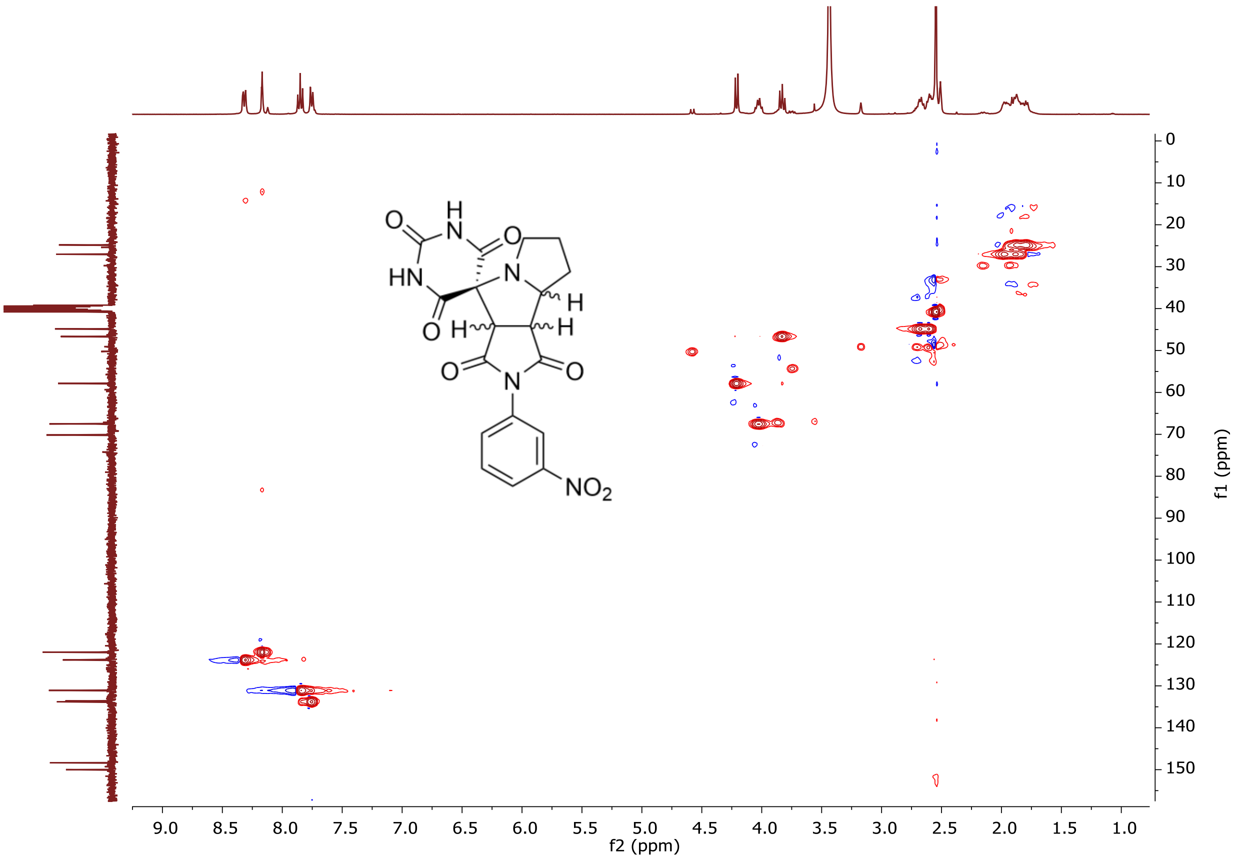

Figure S50. ^{1}H-^{13}C HSQC NMR spectrum (DMSO-d_6) of **4k**

(*rac*)-(*endo,exo*)-2'-(3-Chlorophenyl)hexahydro-1'*H*,2*H*-spiro[pyrimidine-5,4'-pyrrolo[3,4-*a*]pyrrolizine]-1',2,3',4,6(1*H*,2'*H*,3*H*)-pentaone (**4l**)

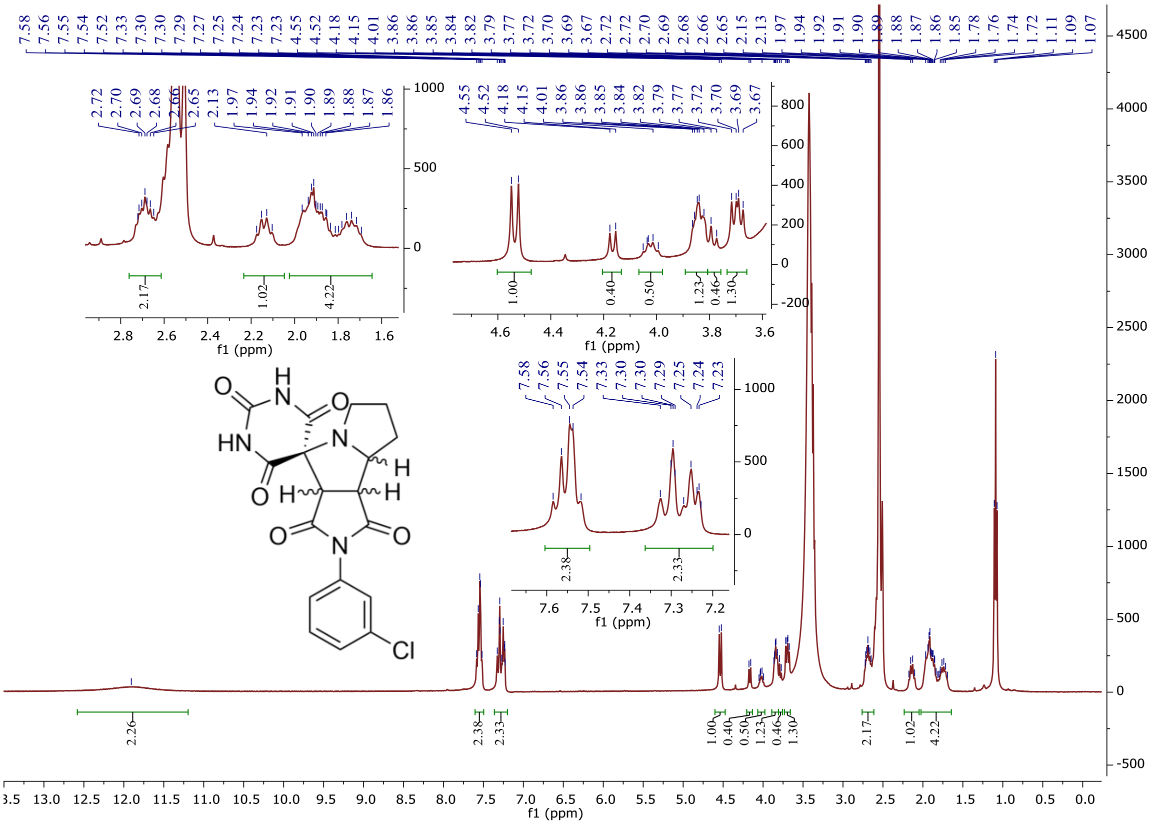

Figure S51. ^{1}H NMR spectrum (DMSO-d_6) of **4l**

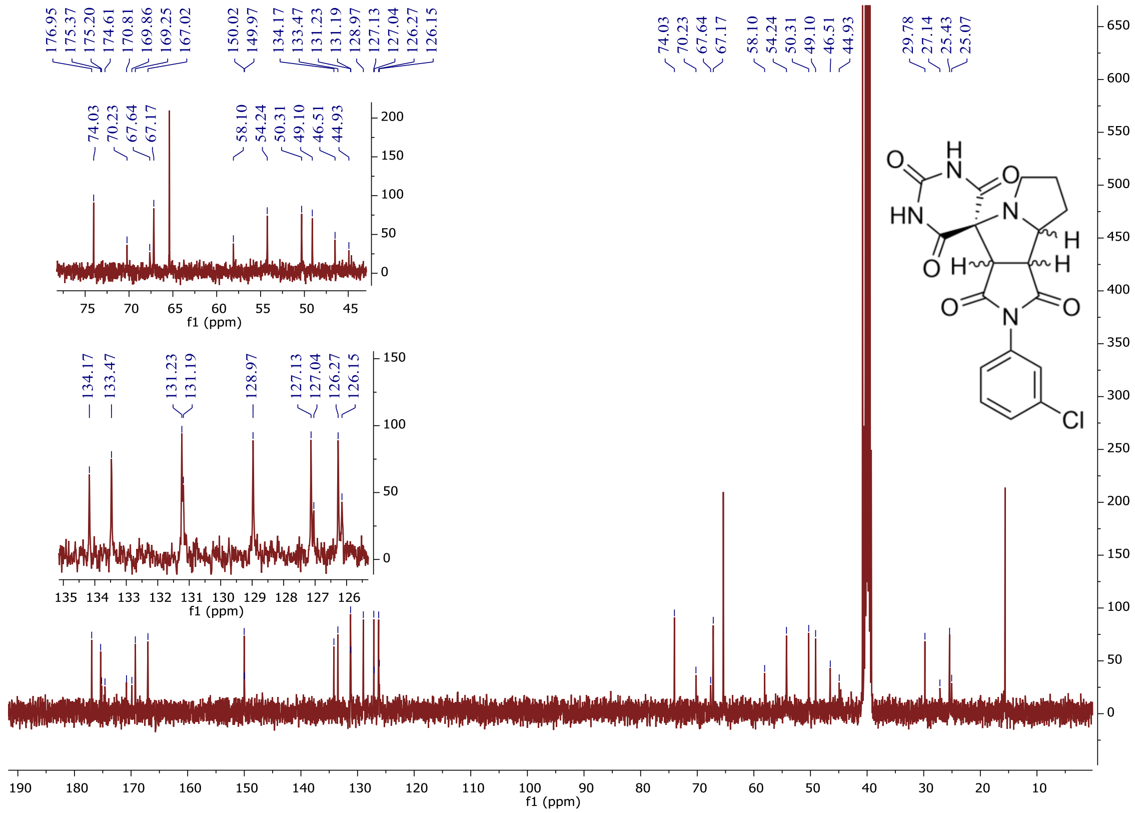

Figure S52. ^{13}C NMR spectrum (DMSO-d_6) of **4l**

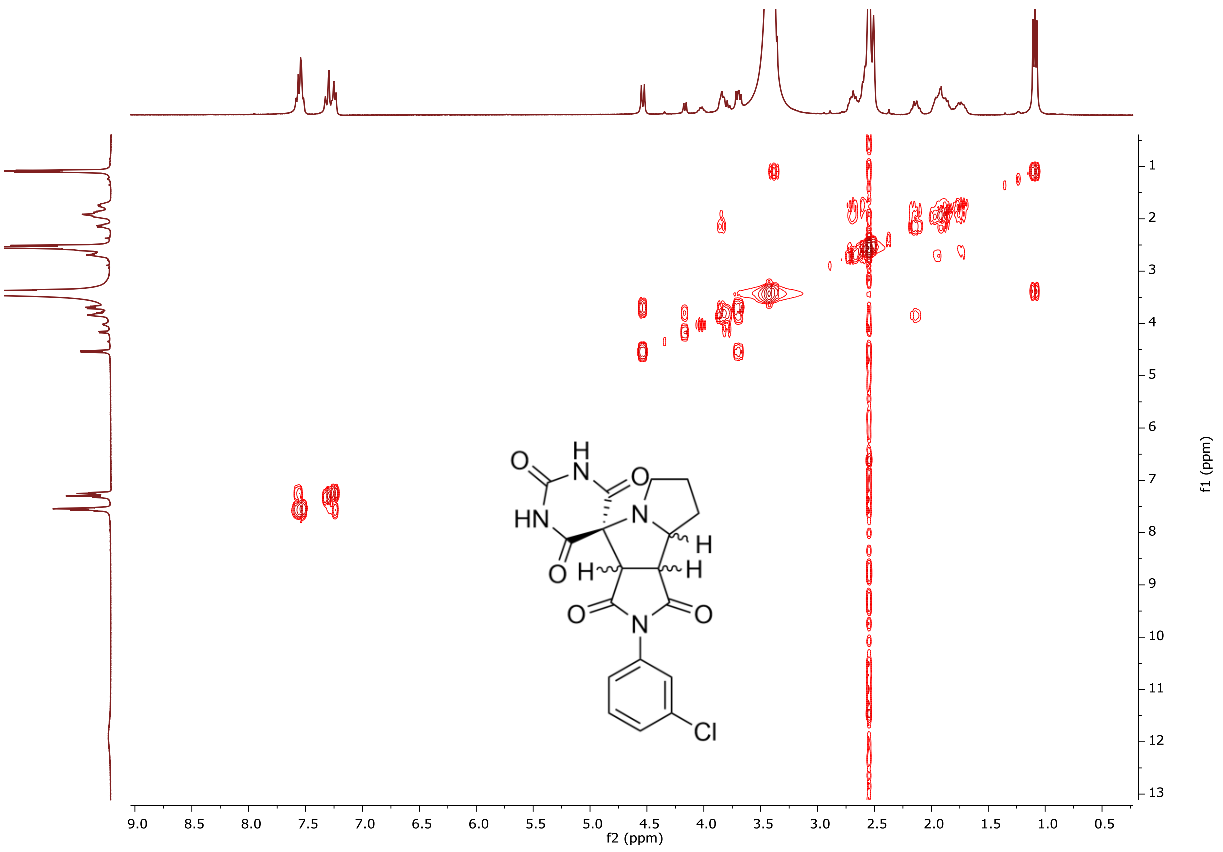

Figure S53. ^{1}H-^{1}H COSY NMR spectrum (DMSO-d_6) of **4l**

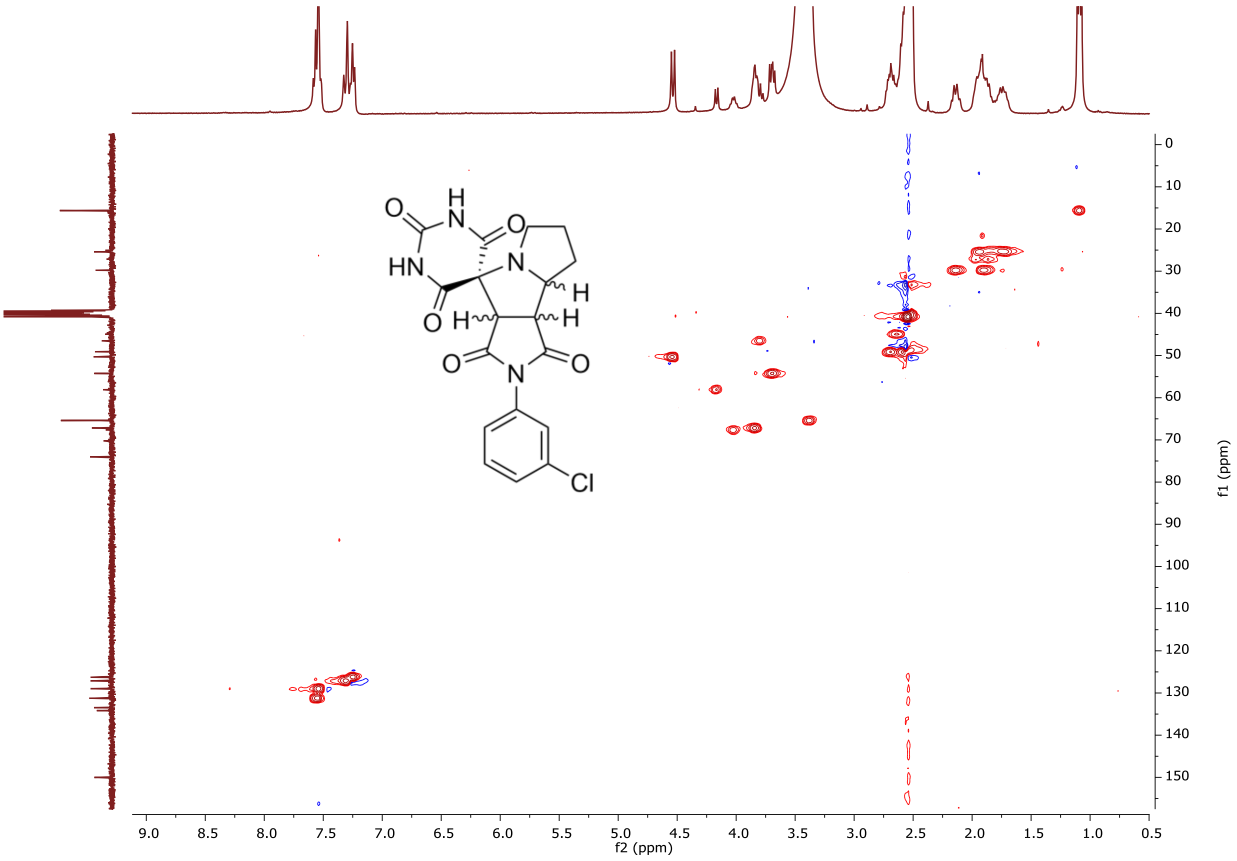

Figure S54. ^{1}H-^{13}C HSQC NMR spectrum (DMSO-d_6) of **4l**

(*rac*)-(*endo,exo*)-2'-(3-(Trifluoromethyl)phenyl)hexahydro-1'*H*,2*H*-spiro[pyrimidine-5,4'-pyrrolo[3,4-*a*]pyrrolizine]-1',2,3,3',4,6(1*H*,2'*H*,3*H*)-pentaone (**4m**)

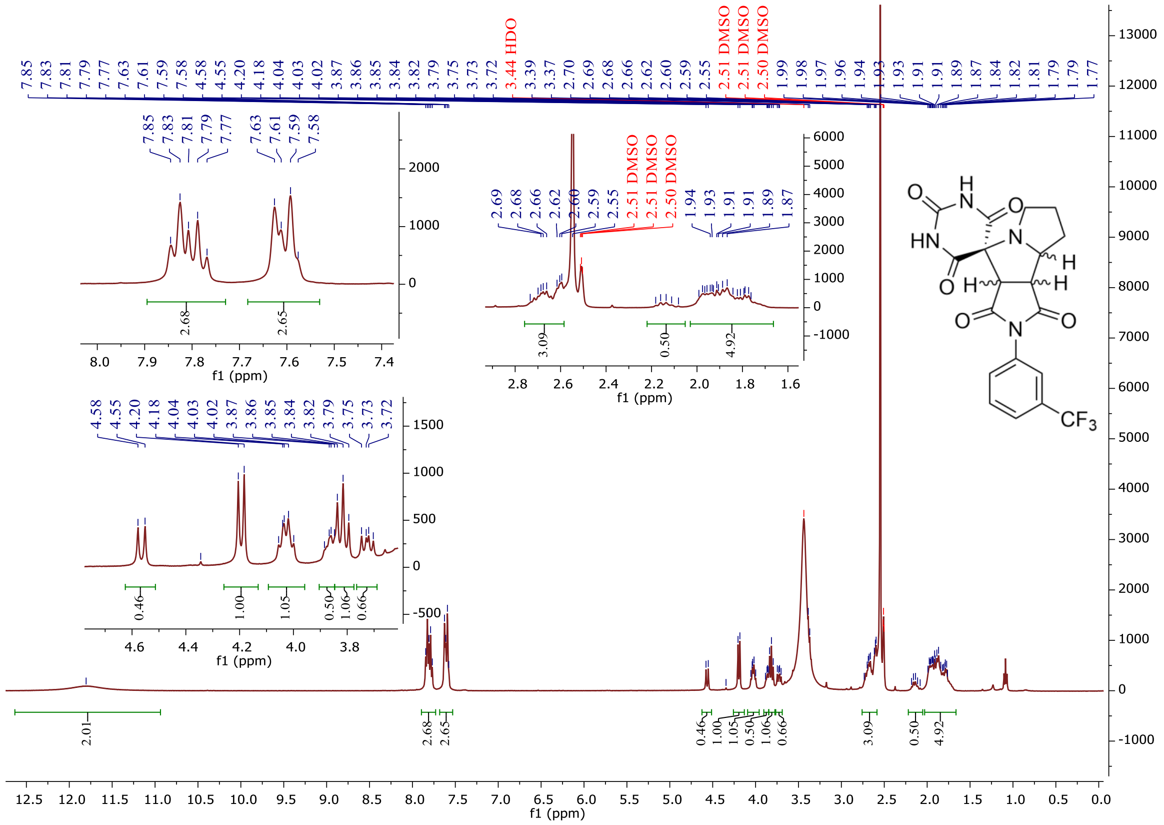

Figure S55. ^{1}H NMR spectrum (DMSO-d_6) of **4m**

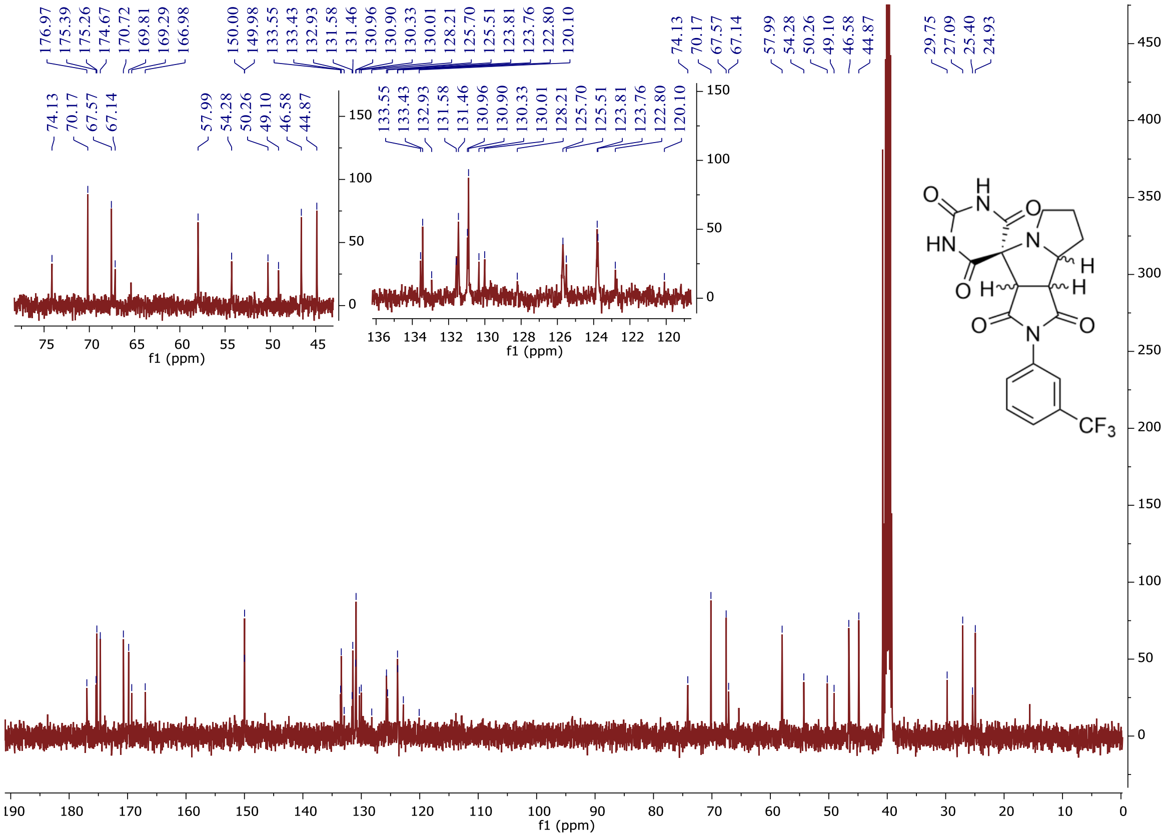

Figure S56. ^{13}C NMR spectrum (DMSO-d_6) of **4m**

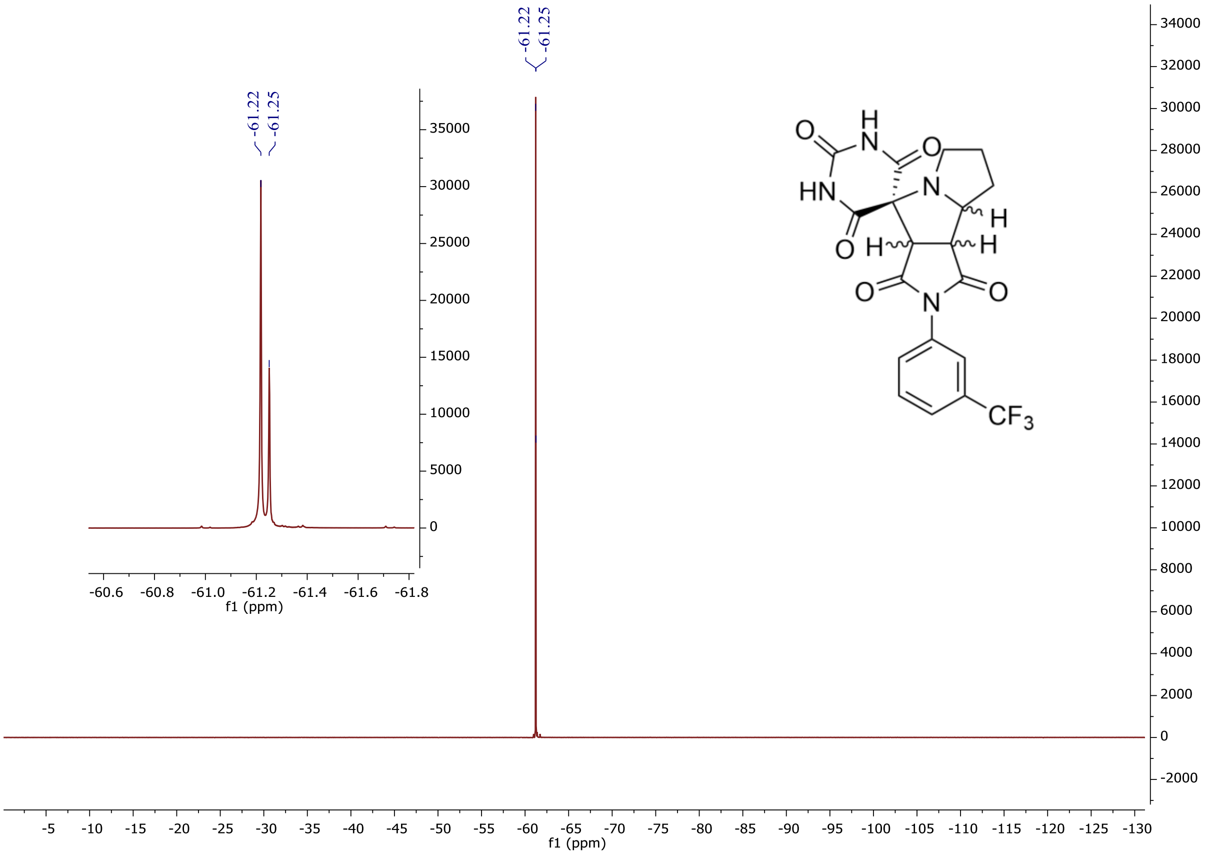

Figure S57. ^{19}F NMR spectrum (DMSO-d_6) of **4m**

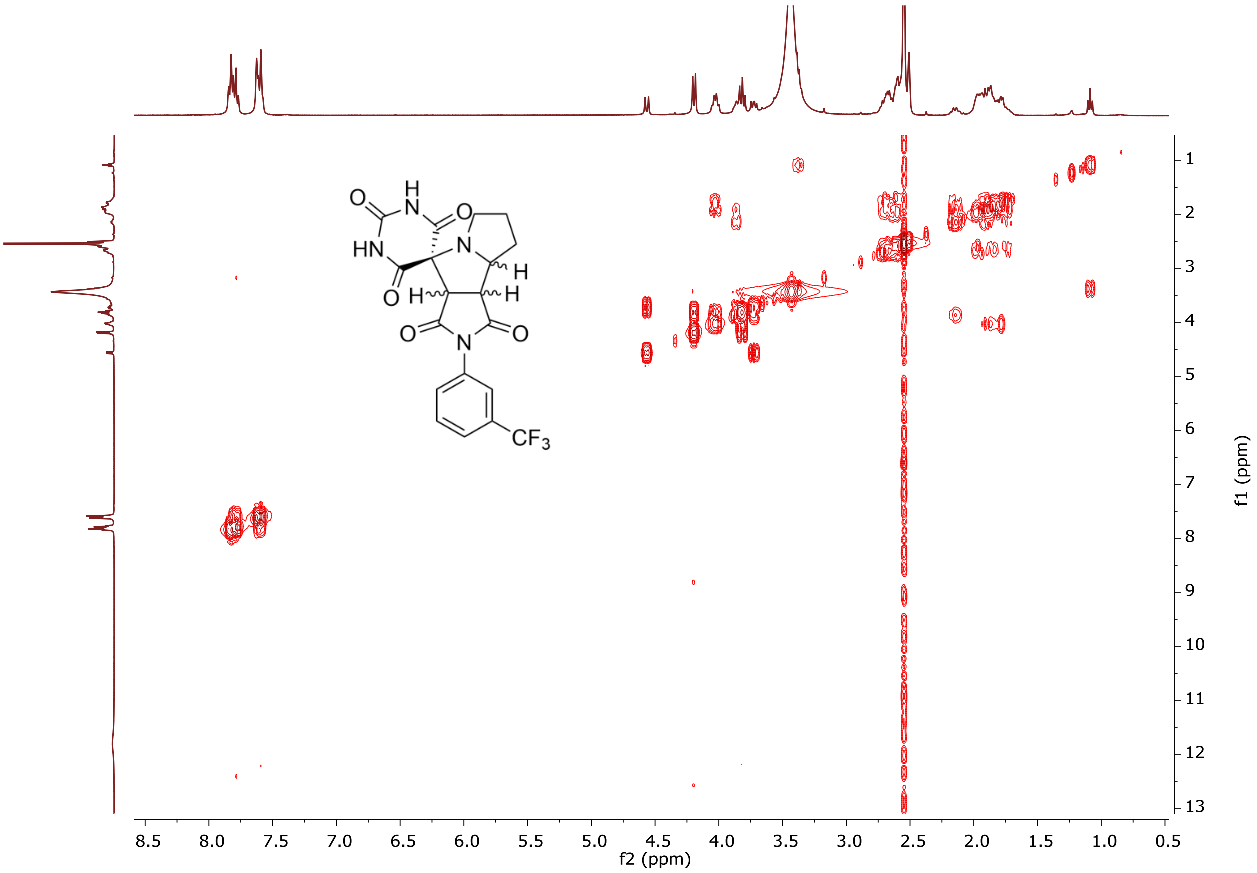

Figure S58. ^{1}H-^{1}H COSY NMR spectrum (DMSO-d_6) of **4m**

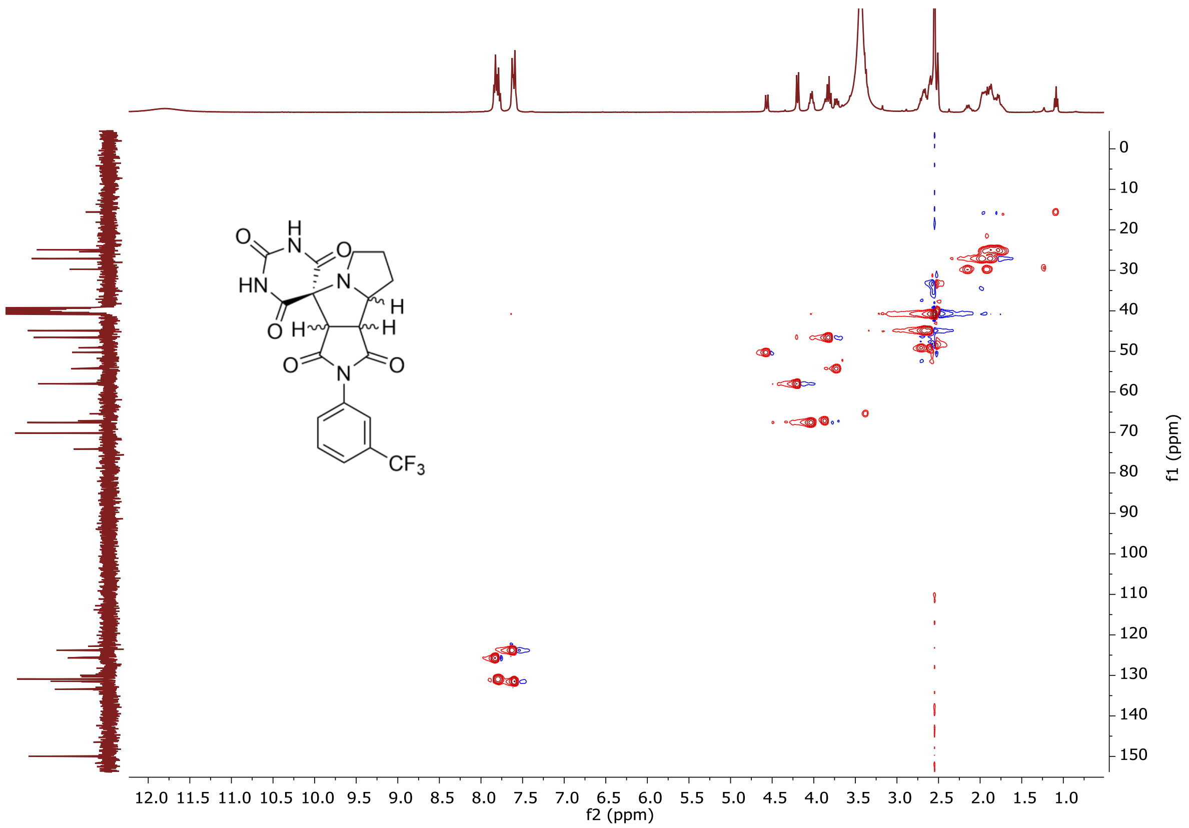

Figure S59. ^{1}H-^{13}C HSQC NMR spectrum (DMSO-d_6) of **4m**

(*rac*)-(*endo,exo*)-2'-(*m*-Tolyl)hexahydro-1'*H*,2*H*-spiro[pyrimidine-5,4'-pyrrolo[3,4-*a*]pyrrolizine]-1',2,3',4,6(1*H*,2'*H*,3*H*)-pentaone (**4n**)

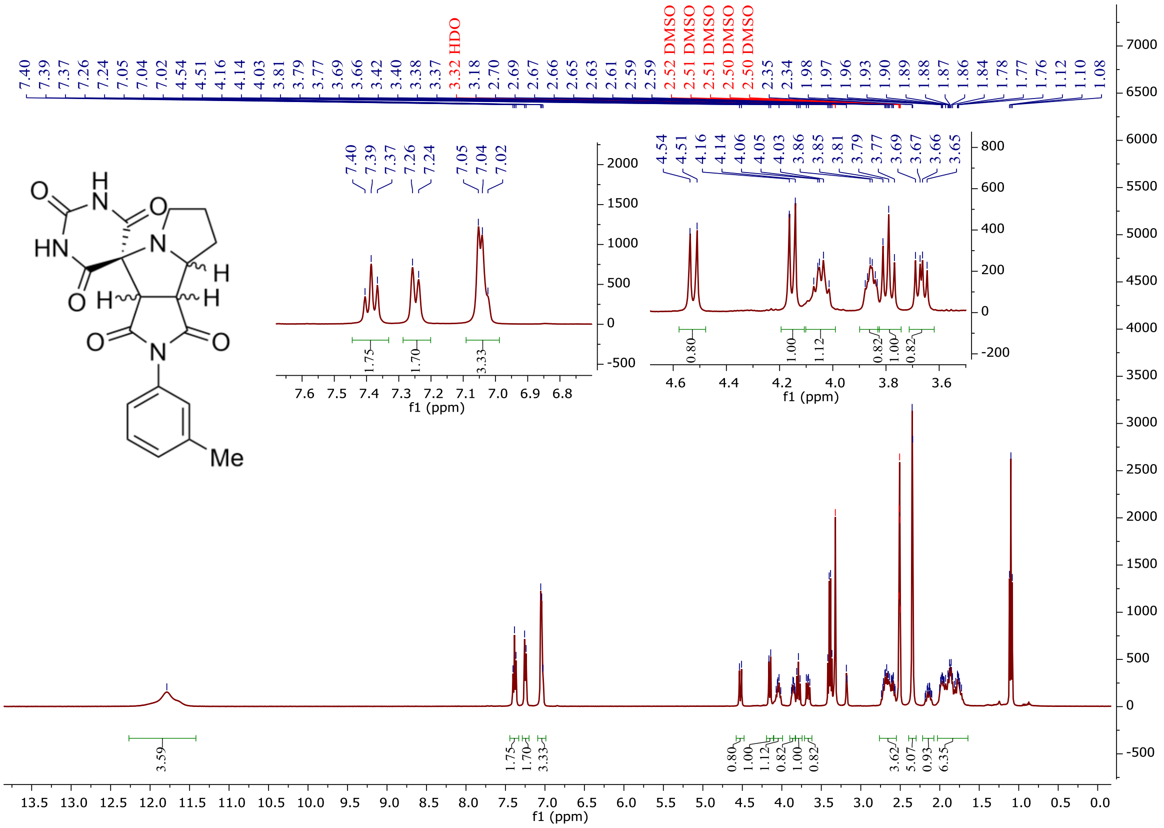

Figure S60. ^{1}H NMR spectrum (DMSO-d_6) of **4n**

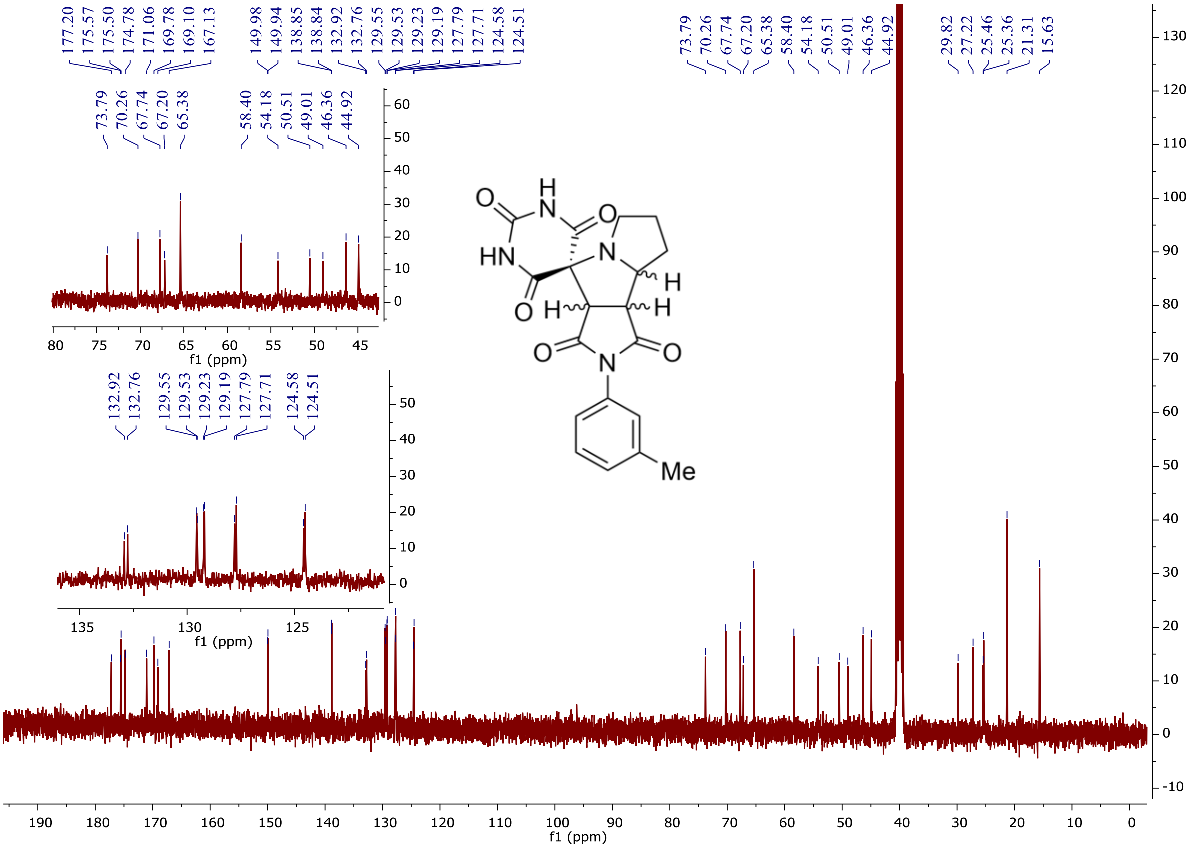

Figure S61. ^{13}C NMR spectrum (DMSO-d_6) of **4n**

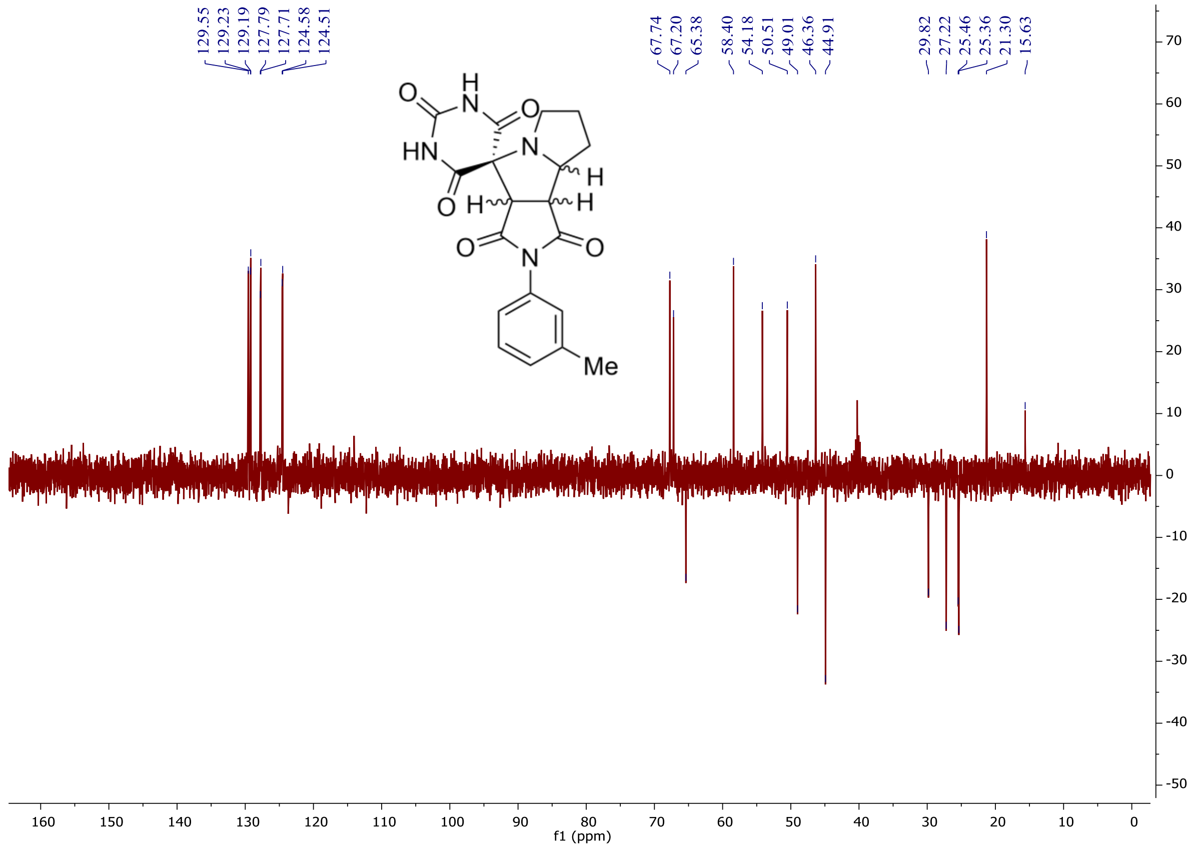

Figure S62. ^{13}C DEPT NMR spectrum (DMSO-d_6) of **4n**

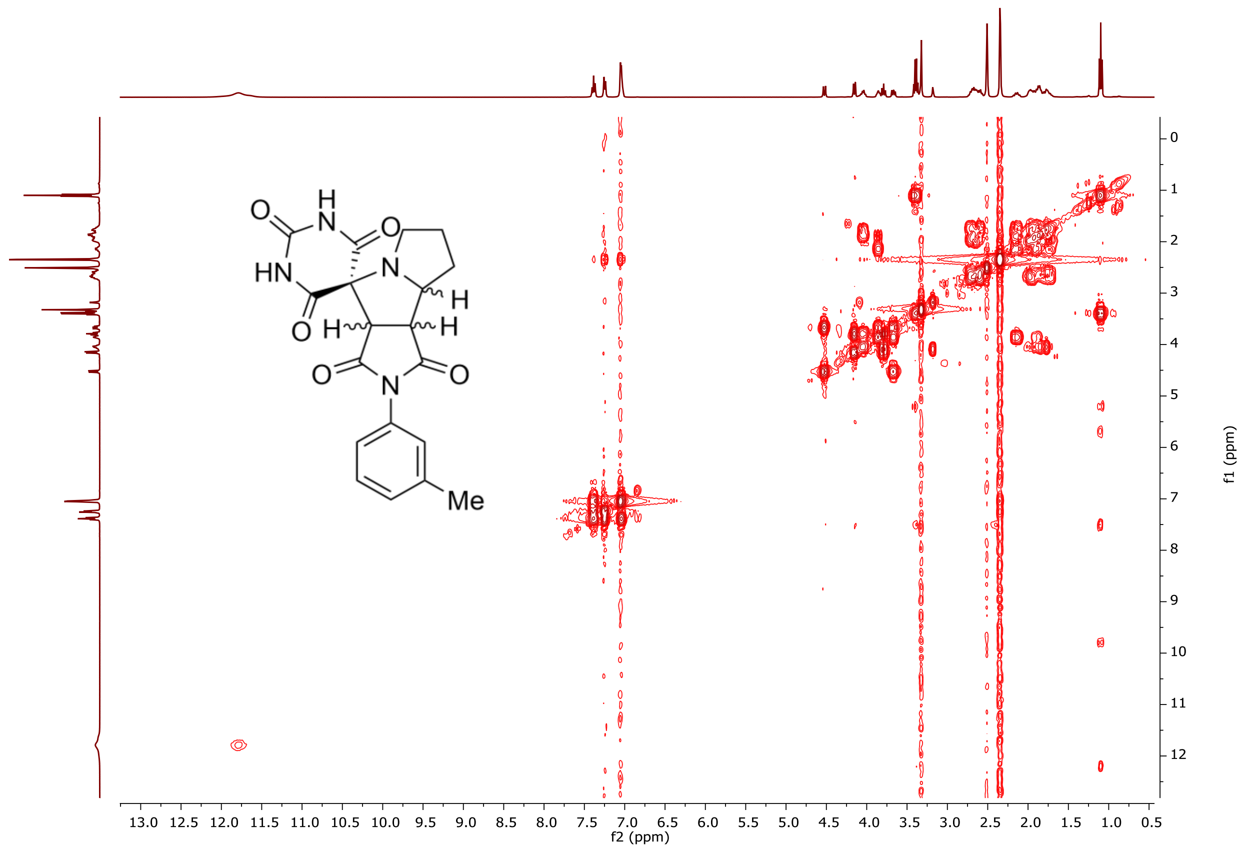

Figure S63. ^{1}H-^{1}H COSY NMR spectrum (DMSO-d_6) of **4n**

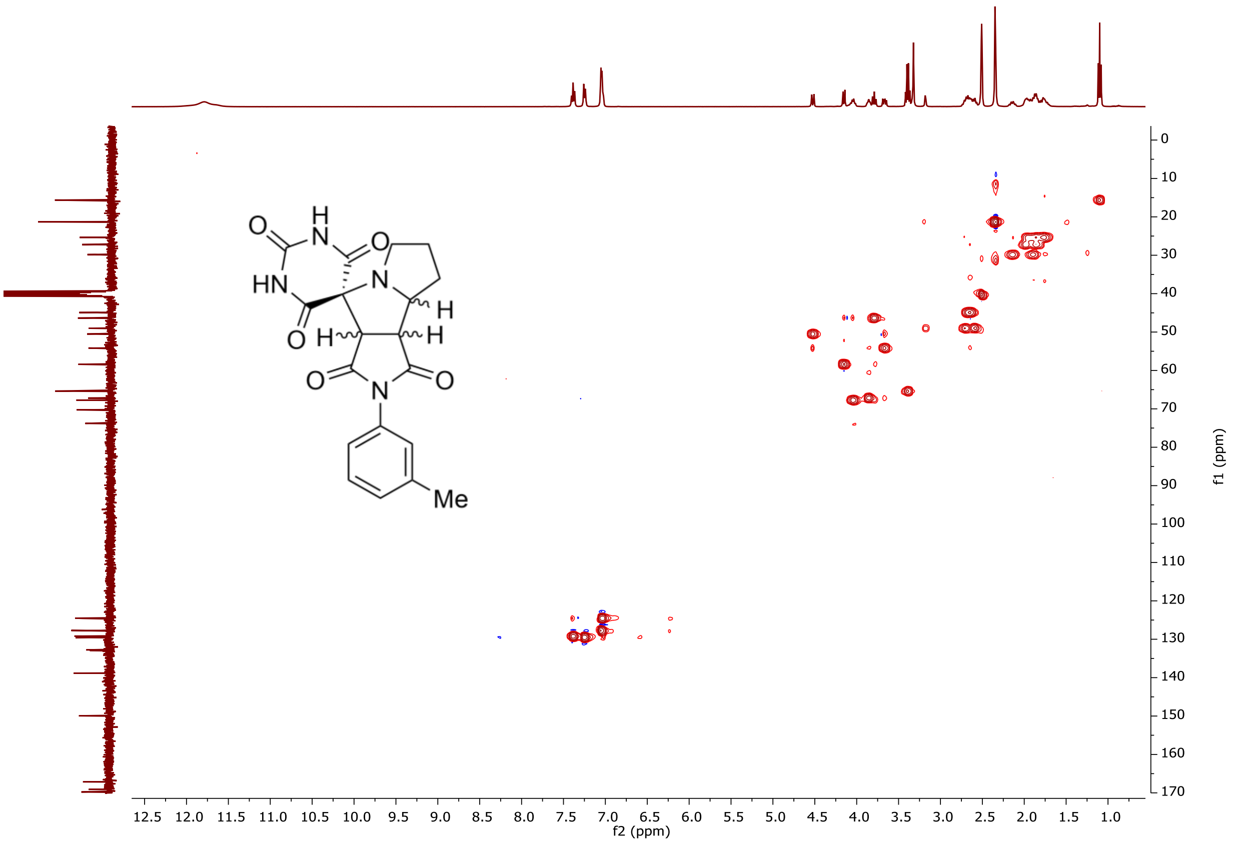

1) **Figure S64.** ^{1}H-^{13}C HSQC NMR spectrum (DMSO-d_6) of **4n**

(*rac*)-(*endo,exo*)-2’-(3-Methoxyphenyl)hexahydro-1’*H*,2*H*-spiro[pyrimidine-5,4’-pyrrolo[3,4-*a*]pyrrolizine]-1’,2,3’,4,6(1*H*,2’*H*,3*H*)-pentaone (**4o**)

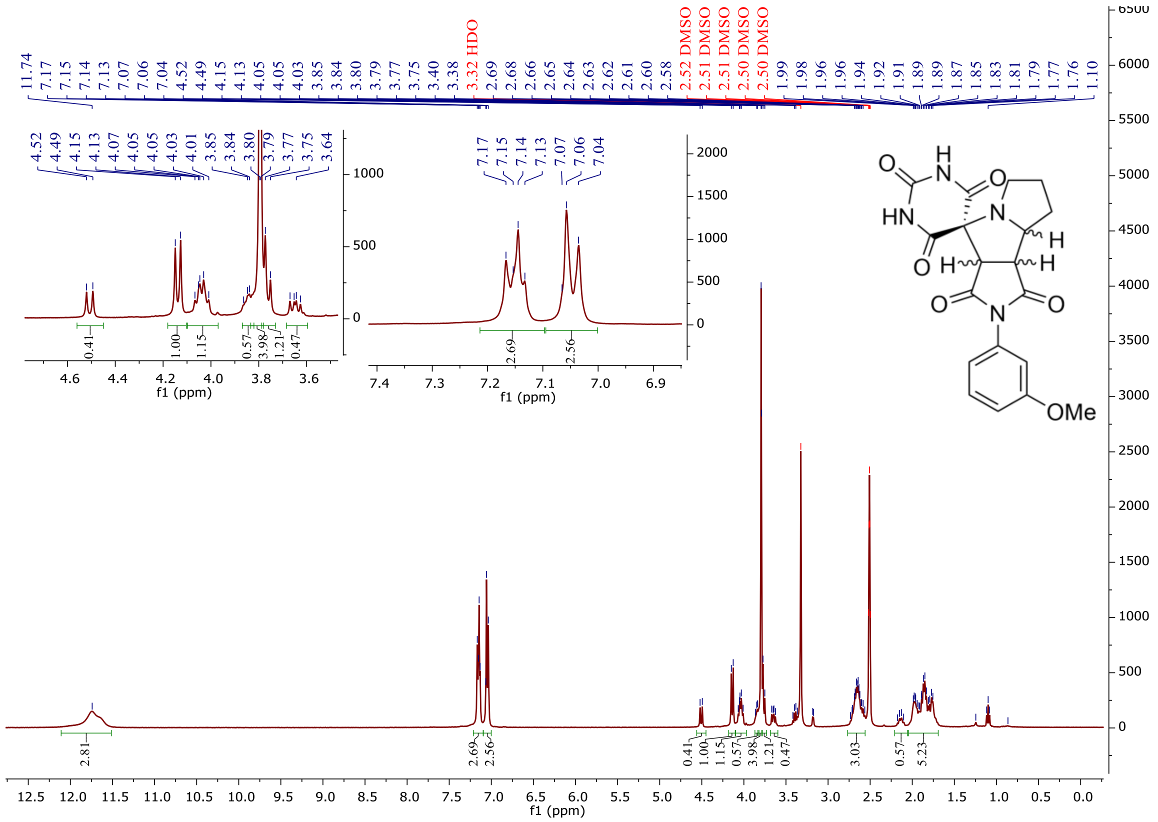

Figure S65. ^{1}H NMR spectrum (DMSO-d_6) of **4o**

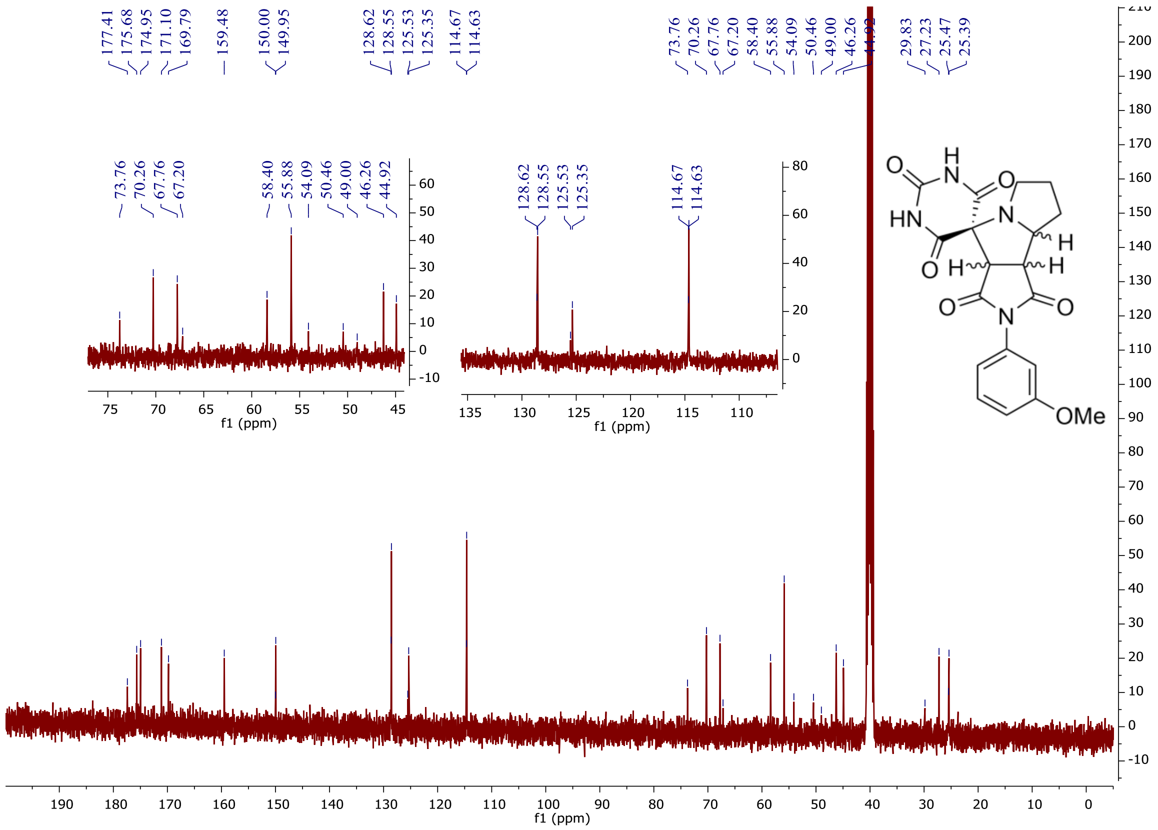

Figure S66. ^{13}C NMR spectrum (DMSO-d_6) of **4o**

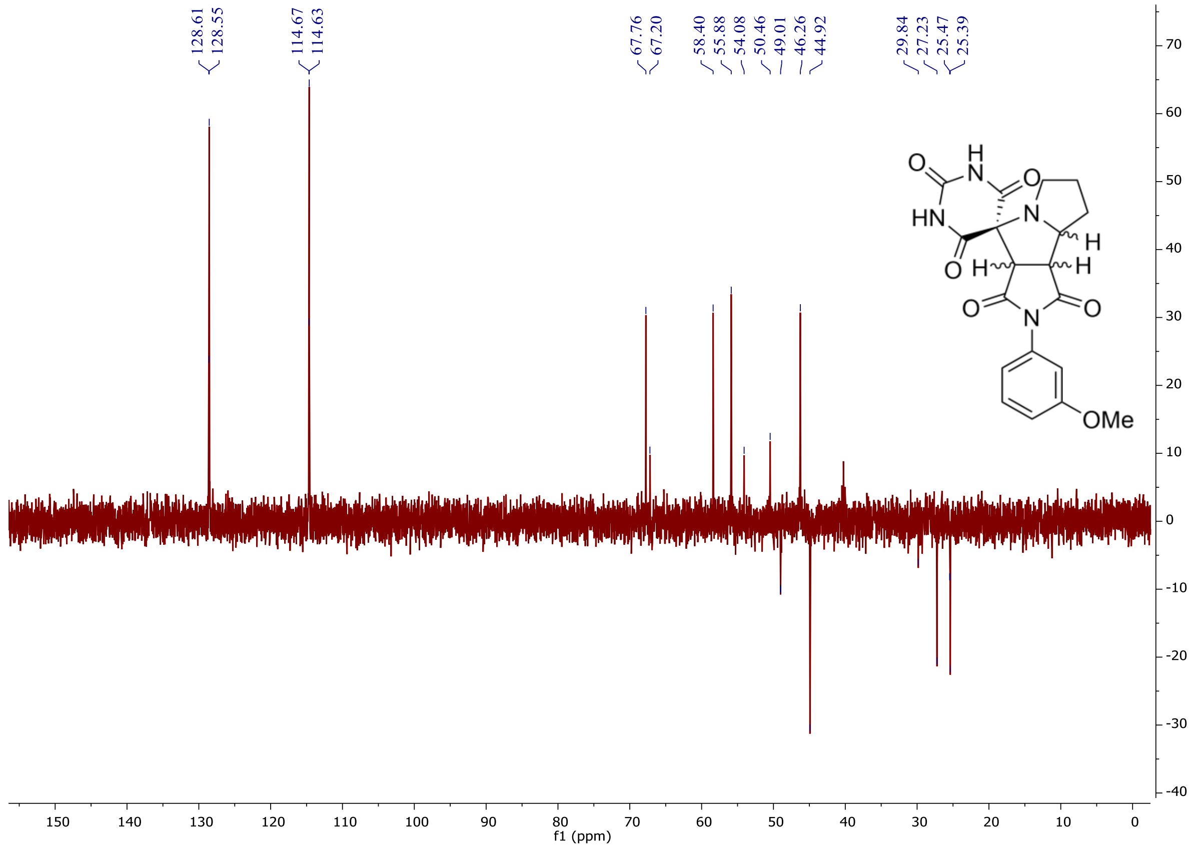

Figure S67. ^{13}C DEPT NMR spectrum (DMSO-d_6) of **4o**

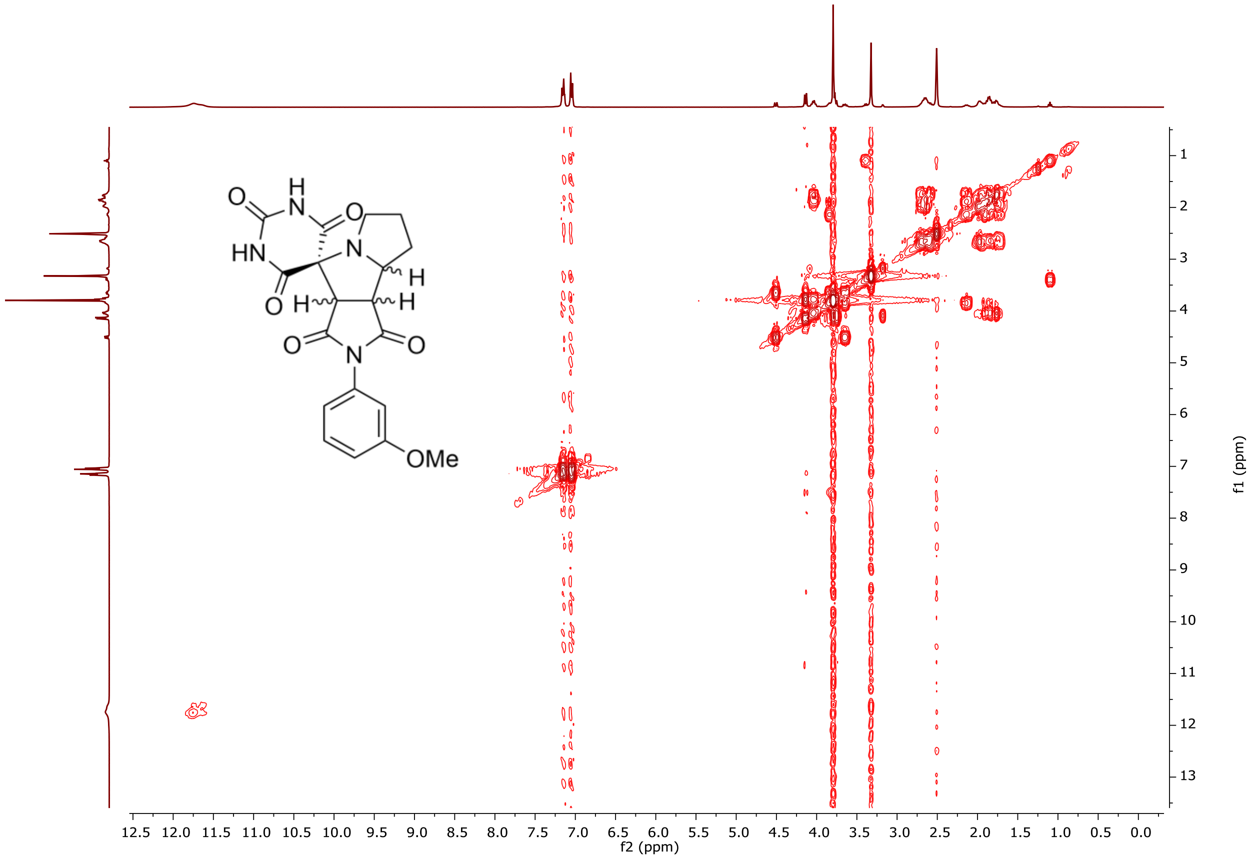

Figure S68. ^{1}H-^{1}H COSY NMR spectrum (DMSO-d_6) of **4o**

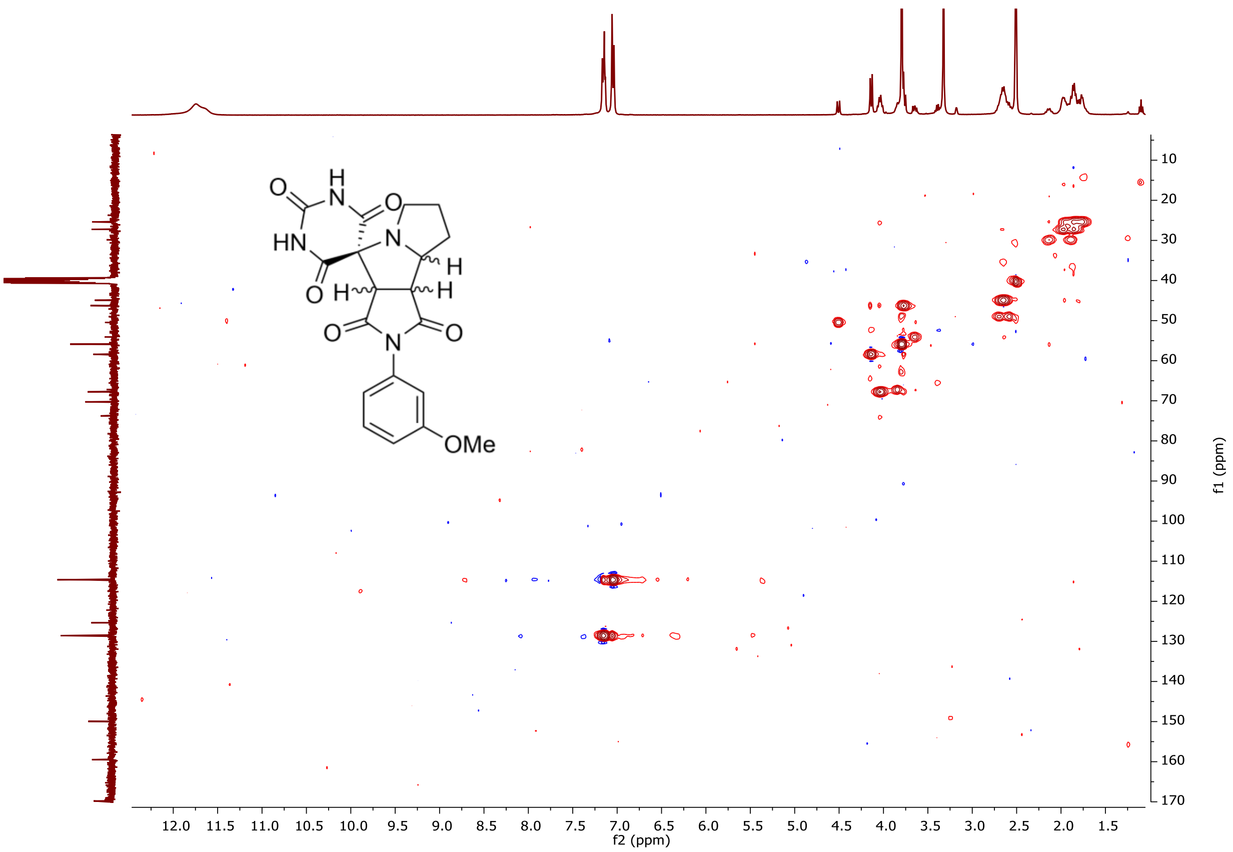

Figure S69. ^{1}H-^{13}C HSQC NMR spectrum (DMSO-d_6) of **4o**

(*rac*)-(*endo,exo*)-2'-(2-Methoxyphenyl)hexahydro-1'*H*,2*H*-spiro[pyrimidine-5,4'-pyrrolo[3,4-*a*]pyrrolizine]-1',2,3',4,6(1*H*,2'*H*,3*H*)-pentaone (**4p**)

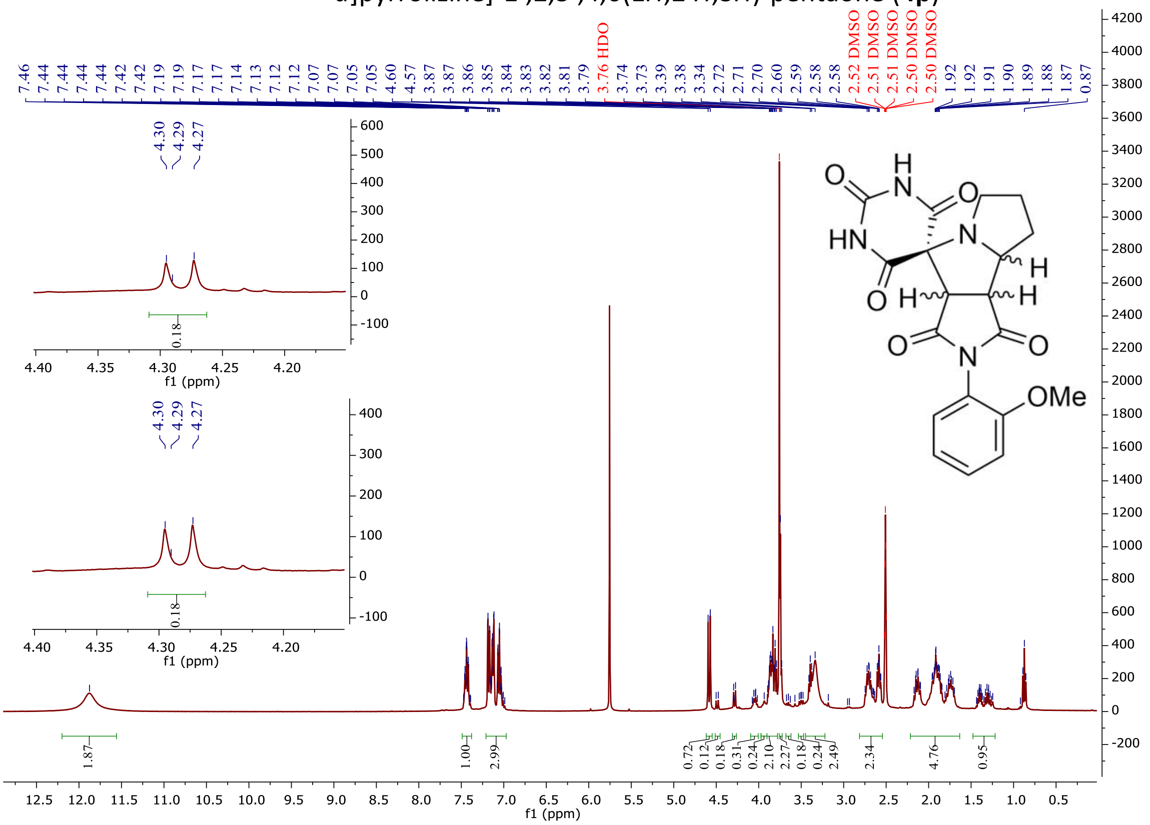

Figure S70. ^{1}H NMR spectrum (DMSO-d_6) of **4p**

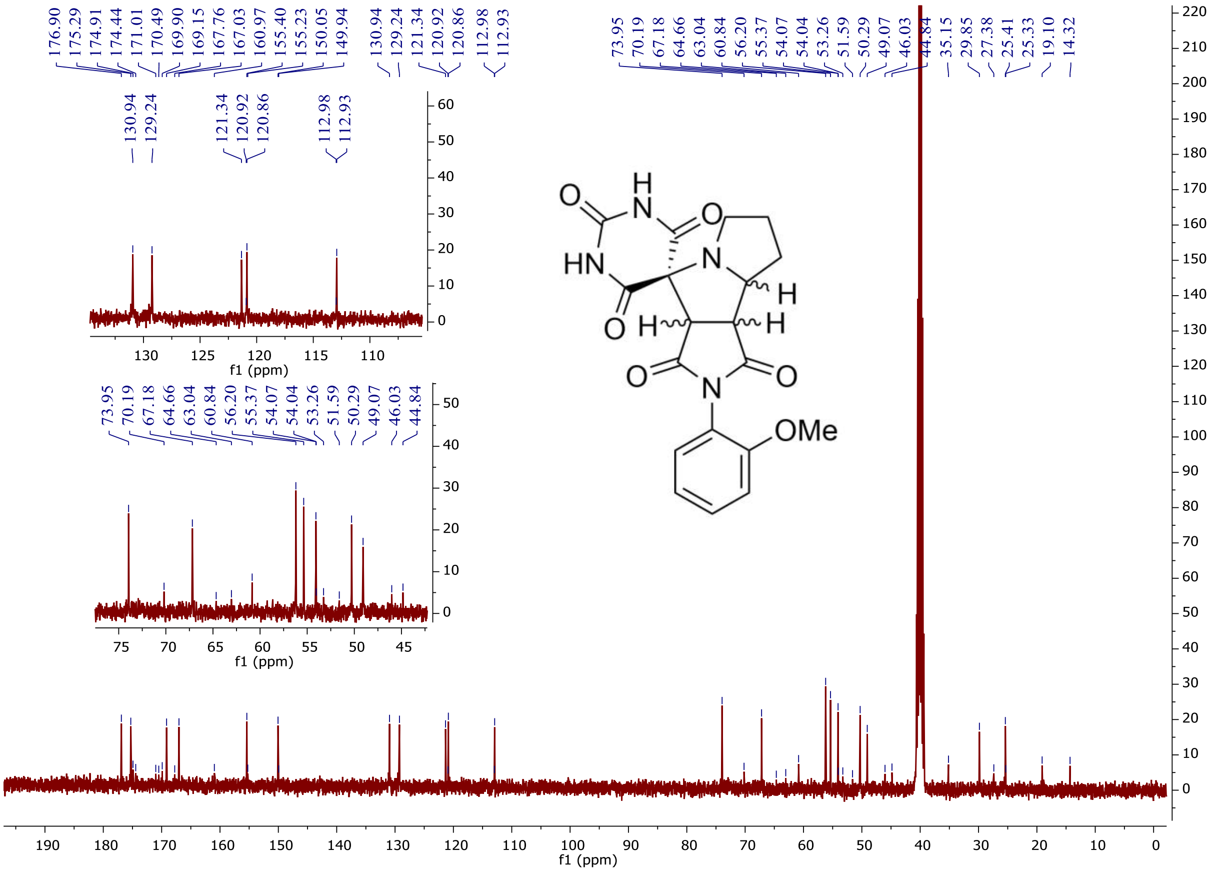

Figure S71. ^{13}C NMR spectrum (DMSO-d_6) of **4p**

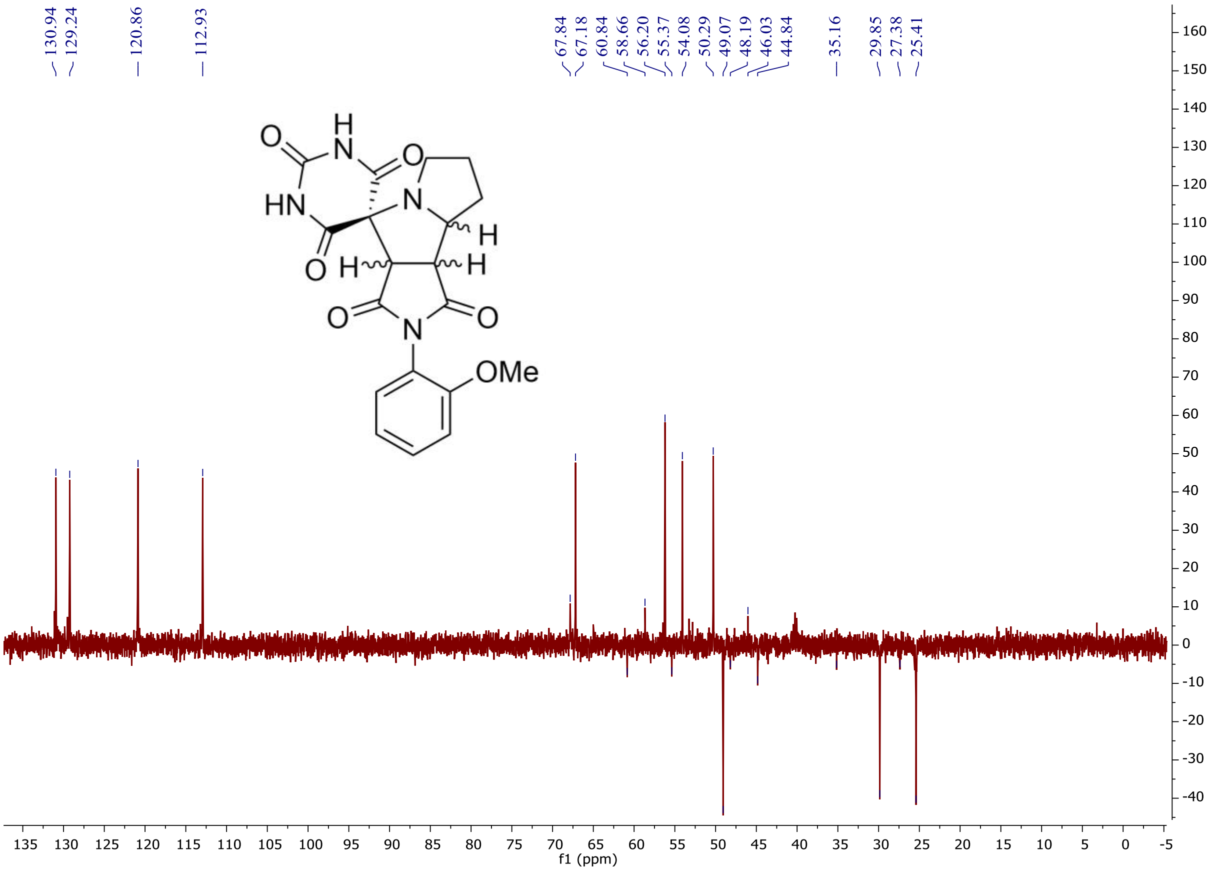

Figure S72. ^{13}C DEPT NMR spectrum (DMSO-d_6) of **4p**

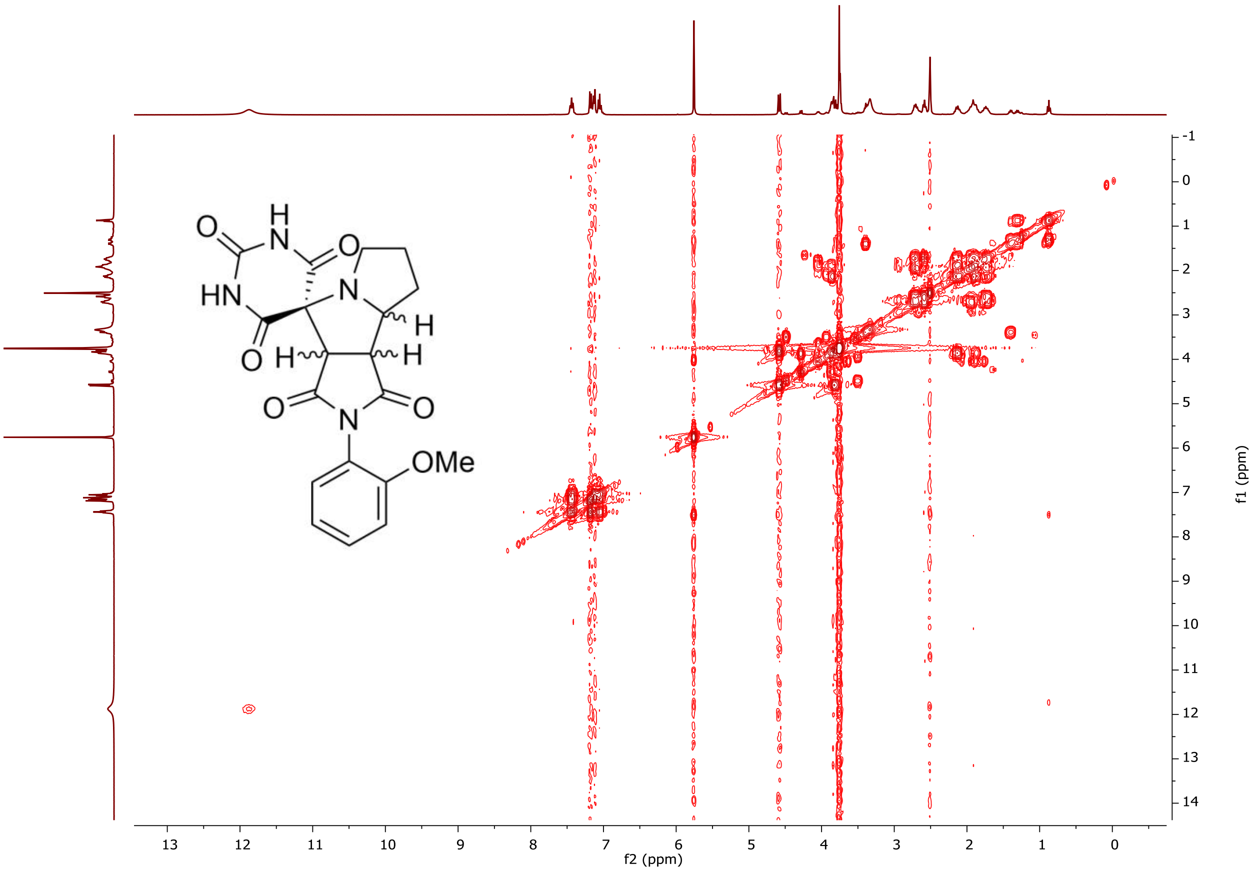

Figure S73. ^{1}H-^{1}H COSY NMR spectrum (DMSO-d_6) of **4p**

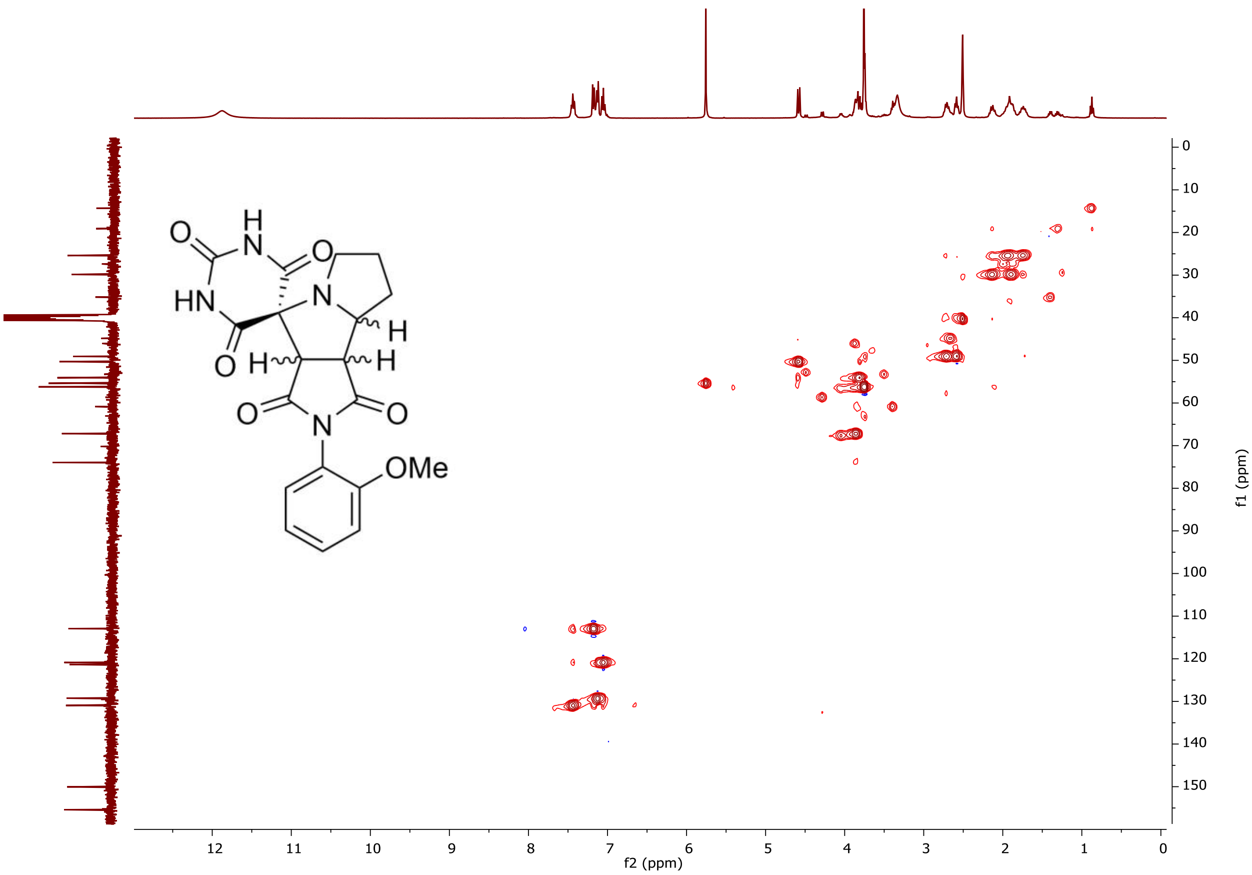

Figure S74. ^{1}H-^{13}C HSQC NMR spectrum (DMSO-d_6) of **4p**

X-Ray crystallographic data for compounds 4b (CCDC 2391172) and 4c (CCDC 2391171)

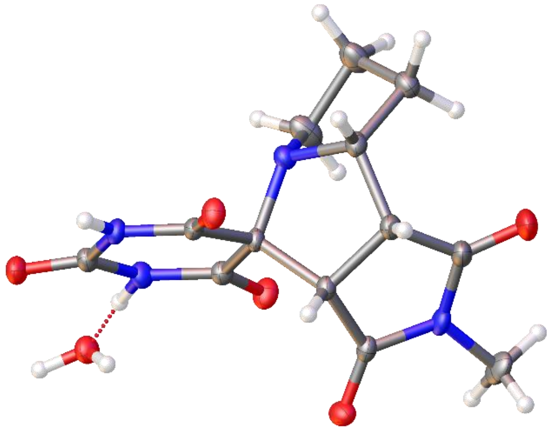

Figure S75. ORTEP representation of the molecular structure of **4b** (CCDC 2391172). Thermal ellipsoids are drawn at 50% probability level.

Table S2. Crystal data for compound **4b**

Empirical formula	$C_{13}H_{16}N_4O_6\cdot H_2O$
Formula weight	324.30
Temperature/K	100(2)
Crystal system	monoclinic
Space group	$P2_1/n$
a/Å	14.7560(11)
b/Å	7.0571(3)
c/Å	14.9476(12)
α/°	90
β/°	117.516(10)
γ/°	90
Volume/Å^3	1380.5(2)
Z	4
ρ_{calc}g/cm^3	1.560
μ/mm^{-1}	1.071
F(000)	680.0
Radiation	Cu Kα (λ = 1.54184)
2Θ range for data collection/°	6.962 to 139.842
Index ranges	-16 ≤ h ≤ 17, -8 ≤ k ≤ 8, -18 ≤ l ≤ 15
Reflections collected	8687
Independent reflections	2565 [R_{int} = 0.0939, R_{sigma} = 0.0627]
Data/restraints/parameters	2565/0/220
Goodness-of-fit on F^2	1.084
Final R indexes [I>=2σ (I)]	R_1 = 0.0720, wR_2 = 0.1915
Final R indexes [all data]	R_1 = 0.0763, wR_2 = 0.2000
Largest diff. peak/hole / e Å^{-3}	0.85/-0.57

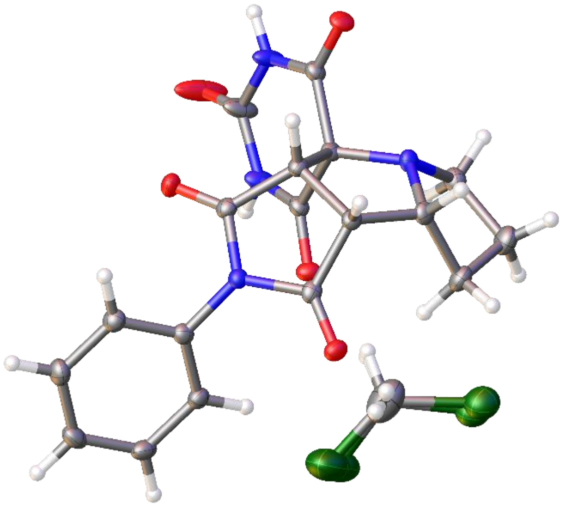

Figure S76. ORTEP representation of the molecular structure of **4c** (CCDC 2391171). Thermal ellipsoids are drawn at 50% probability level.

Table S3. Crystal data for compound **4c**

Empirical formula	$C_{18}H_{16}N_4O_5{\cdot}CH_2Cl_2$
Formula weight	453.27
Temperature/K	100.00(10)
Crystal system	monoclinic
Space group	$P2_1/c$
a/Å	14.2474(2)
b/Å	11.89379(16)
c/Å	12.31795(17)
α/°	90
β/°	103.4937(15)
γ/°	90
Volume/Å^3	2029.73(5)
Z	4
ρ_{calc}g/cm^3	1.483
μ/mm^{-1}	3.234
F(000)	936.0
Radiation	Cu Kα (λ = 1.54184)
2Θ range for data collection/°	6.38 to 139.998
Index ranges	-17 ≤ h ≤ 17, -14 ≤ k ≤ 14, -14 ≤ l ≤ 12
Reflections collected	15742
Independent reflections	3839 [R_{int} = 0.0394, R_{sigma} = 0.0317]
Data/restraints/parameters	3839/0/278
Goodness-of-fit on F^2	1.055
Final R indexes [I>=2σ (I)]	R_1 = 0.0436, wR_2 = 0.1139
Final R indexes [all data]	R_1 = 0.0483, wR_2 = 0.1174
Largest diff. peak/hole / e Å^{-3}	0.47/-0.61

Biological activity study

In silico analysis: The molecular descriptors of the synthesized spiro-fused adducts were determined with using the free online software SwissADME (http://www.swissadme.ch/ accessed on 19 February 2025) and analyzed according to Lipinski's rule. ADMET profiling was estimated in silico using the PreADMET 2.0 online software (https://preadmet.webservice.bmdrc.org/ accessed on 19 February 2025).

Molecular docking: Molegro Virtual Docker 6.0 software was used to perform molecular docking. The crystal structure data were obtained from the Protein Data Bank (8DNH). The target structures were prepared automatically using standard procedures of the Molegro Virtual Docker package. The chemical structures of the ligands were drawn using ChemBioDraw Ultra 13.0 and optimized by MM2 calculations in Chem3D Pro 13.0. The MolDock Score was used as a scoring function. There were 20 trial runs for calculations. MolDock SE was used as a docking algorithm following energy minimization and optimization of hydrogen bonds. Visualization of molecular docking results and analysis of ligand-protein interactions were performed using BIOVIA Discovery Studio Visualizer 2021.

Cell cultures and culturing conditions: The human cervical carcinoma (HeLa) and erythroleukemia (K-562) were obtained from the Bank of Cell Cultures of the Institute of Cytology of the Russian Academy of Sciences. the human melanoma (Sk-mel-2) cell line was obtained from the Bank of Cell Cultures of the Institute of Cytology and genetics, Siberian Branch of Russian Academy of Sciences. K-562 cells were cultured in RPMI medium (Hyclone) supplemented with fetal bovine serum (FBS, 10% v/v, Hyclone) and gentamicin. HeLa and Sk-mel-2 cells were cultured in Dulbecco's Modified Eagle's Medium (DMEM) with the same supplements. All the cell lines were maintained under controlled conditions: a humid atmosphere with 5% CO_2 at 37 °C.

Cell proliferation assay: Cell viability was measured in vitro using the 3-(4,5-dimethylthiazol-2-yl)-5-(3-carboxymethoxyphenyl)-2-(4-sulfophenyl)-2*H*-tetrazolium (MTS) assay. In short, cells were seeded into 96-well microtiter plates at a density of 5×10^3 cells per well in 100 µL of complete medium and allowed to grow and adhere onto the wells for 24 h at 37 °C. After that, the cells were treated with various concentrations of the compounds for a period of 24 and 72 h. After the treatment, 20 µL of MTS reagent stock solution was added into each well and incubated at 37 °C for 2 h in a humidified, 5% CO_2 atmosphere. Finally, the absorbance was recorded at 495 nm using 96-well plate reader 'Multiskan gO'. All samples were measured in triplicate.

Actin cytoskeleton staining: Sk-mel-2 cells were seeded onto a Petri dish with cover slips at a density of 2×10^5 cells per dish and incubated for 24 h. After that, cells were treated with chosen compounds (10 µg/mL) for 24 h. The medium was removed, cells were fixed with 4% paraformaldehyde, washed with PBS three times, and permeabilized with 0.3% Triton-X100. The cells were rinsed with PBS three times. Actin filaments (microfilaments) were stained at 37 °C for 15 min with rhodamine-phalloidin. The samples were rinsed with PBS three times, followed by embedding in Fluoroshield medium. The intensity of the staining of preparations was estimated using an AxioObserver Z1 confocal microscope.

Evaluation of cell motility by scratch test: Cells were seeded onto Petri dishes at a density of 5×10^5 cells per dish and grown to confluency. Scratch wounds were made by a 200 µL pipette tip and detached cells were removed after that by washing with PBS. Culture media was replaced with serum-free DMEM in order to inhibit cell proliferation. Compounds to be screened were added to the cultures at a 5 µg/mL concentration and incubated for 24 h. Different fields were analyzed by a bright field, and each scratch area was photographed at 0 and 24 h. Images were captured using an Axio Observer Z1 confocal microscope (Carl Zeiss MicroImaging gmbH, Jena, germany). The percent of wound closure in five randomly chosen fields was calculated with NIH ImageJ software.

Statistical analysis: Statistical analysis was performed using Statistica 6.0. All data from the three independent experiments were used for measuring the means ± standard deviation (mean ± SD), which were compared using the Student's t-test.

Table S4. Physicochemical profiles of compounds according to Lipinski's rule.

Compound	MW	nHBD	nHBA	Log P	nRotB	TPSA, Å^2	$N_{Violation}$	Meet Lipinski Criteria
	<500	<5	<10	≤5	<10	<140	<1	Yes/No
**4a**	292.25	3	6	-0.92	0	124.68	0	Yes
**4b**	306.27	2	6	-1.06	0	115.89	0	Yes
**4c**	368.34	2	6	0.63	1	115.89	0	Yes
**4d**	382.37	2	6	0.45	1	115.89	0	Yes
**4e**	413.34	2	8	-0.26	2	161.71	0	Yes
**4f**	382.37	2	6	0.59	6	115.89	0	Yes
**4g**	396.40	2	8	0.82	3	115.89	0	Yes
**4h**	402.79	2	6	0.72	1	115.89	0	Yes
**4i**	437.23	2	6	1.22	1	115.89	0	Yes
**4j**	420.78	2	7	1.10	1	115.89	0	Yes
**4k**	413.34	2	8	-0.18	2	145.32	0	Yes
**4l**	402.79	2	6	0.72	1	115.89	0	Yes
**4m**	436.34	2	9	1.06	2	115.89	0	Yes
**4n**	382.37	2	6	0.86	1	115.89	0	Yes
**4o**	398.37	2	7	0.36	2	125.12	0	Yes
**4p**	398.37	2	7	0.36	2	125.12	0	Yes

MW: molecular weight; nHBD: number of hydrogen-bond donors; nHBA: number of hydrogen bond acceptors; Log P: logarithm of partition coefficient of the compound between n-octanol and water; nRotB: number of rotatable bonds; TPSA: topological polar surface area; $N_{Violation}$: number of violated criteria.

Table S5. ADMET profiles of selected compounds.

Comp.	HIA, %	Caco2, nm/sec	BBB	PPB, %	CYP_2D6 inhibition	Solubility, mg/L	Carcinogenicity (rat / mouse)	Mutagenicity	hERG inhibition
**4a**	46.17	21.10	0.24	8.48	non	66439.4	positive / positive	mutagen	low risk
**4b**	61.90	21.09	0.08	10.04	non	106362	positive / negative	mutagen	low risk
**4c**	87.25	20.83	0.02	30.72	non	2229.56	negative / positive	non-mutagen	low risk
**4d**	88.72	20.90	0.02	34.02	non	654.424	negative / positive	non-mutagen	low risk
**4e**	48.00	20.84	0.01	39.82	non	511.286	negative / positive	mutagen	low risk
**4f**	88.38	20.92	0.05	28.27	non	2209.02	positive / negative	non-mutagen	low risk
**4g**	89.71	20.98	0.05	31.54	inhibitor	3727.48	positive / negative	non-mutagen	ambiguous
**4h**	92.81	20.41	0.09	42.01	non	314.038	negative / positive	non-mutagen	low_risk
**4i**	95.11	19.67	0.03	59.53	non	46.2102	negative / positive	non-mutagen	ambiguous
**4j**	92.86	20.15	0.03	47.80	non	156.088	negative / positive	non-mutagen	low_risk
**4k**	48.00	19.04	0.05	30.93	non	538.076	negative / positive	mutagen	low_risk
**4l**	92.813	20.12	0.01	39.96	non	330.493	negative / positive	non-mutagen	low_risk
**4m**	88.99	20.63	0.08	36.06	non	412.536	negative / positive	non-mutagen	low_risk
**4n**	88.72	20.90	0.02	31.35	non	688.715	negative / positive	non-mutagen	low_risk
**4o**	84.61	20.62	0.01	33.08	non	1572.39	negative / positive	non-mutagen	ambiguous
**4p**	84.62	21.01	0.05	28.86	non	1494.11	negative / positive	non-mutagen	ambiguous

HIA: human intestinal absorption; BBB: *in vivo* blood-brain barrier penetration (C.brain/C.blood); PPB: in vitro plasma protein binding; Caco2: in vitro Caco-2 cell permeability (nm/sec); CYP_2D6_inhibition: in vitro Cytochrome P450 2D6 inhibition; Solubility: water solubility in pure water mg/L); Carcinogenicity: 2 years carcinogenicity bioassay in rat and mouse; Mutagenicity: mutagenicity according to Ames test; hERG inhibition: in vitro Human ether-a-go-go related gene channel inhibition

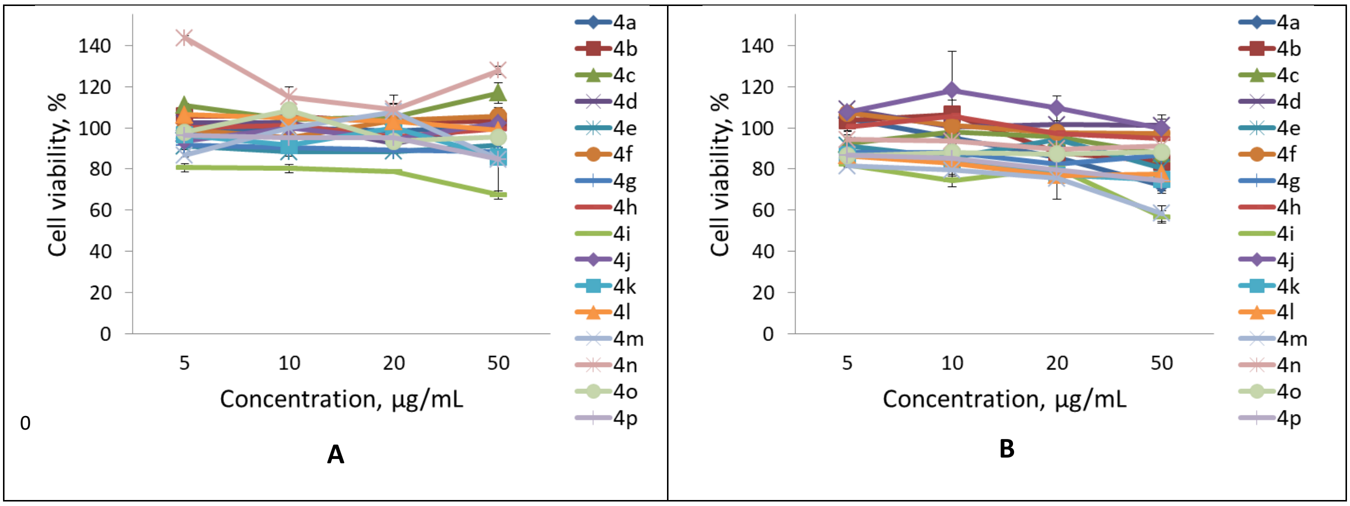

Figure S77. Cytotoxicity of racemic adducts **4a**–**p** against K562 cell line for 24h (**A**) and 72 h (**B**).

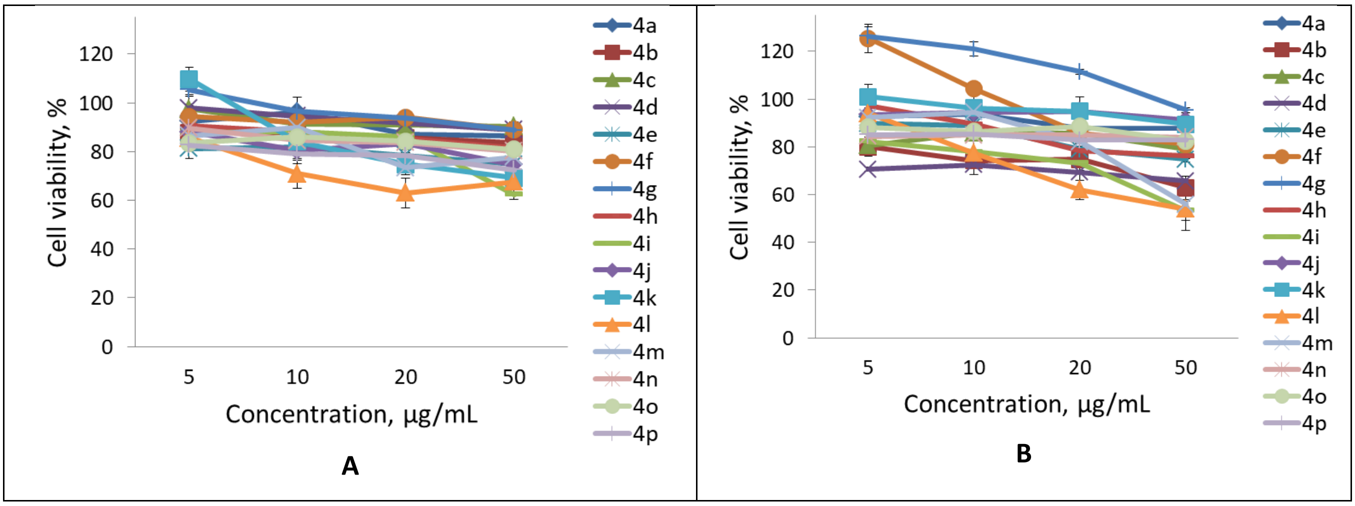

Figure S78. Cytotoxicity of racemic adducts **4a**–**p** against Sk-mel-2 cell line for 24h (**A**) and 72 h (**B**).

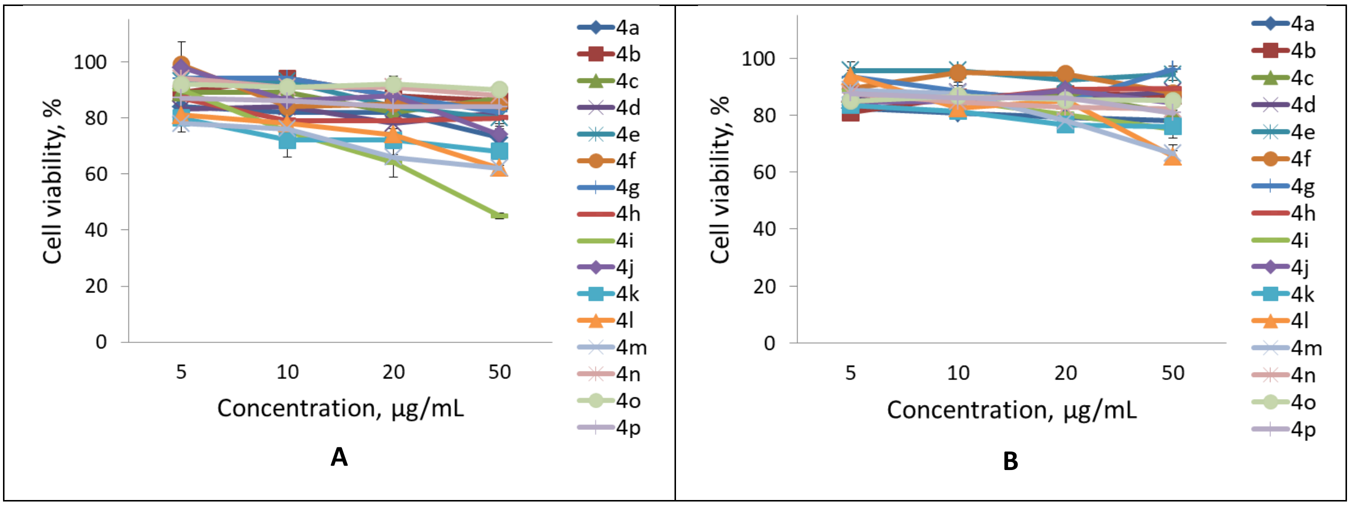

Figure S79. Cytotoxicity of racemic adducts **4a**–**p** against HeLa cell line for 24h (**A**) and 72 h (**B**).

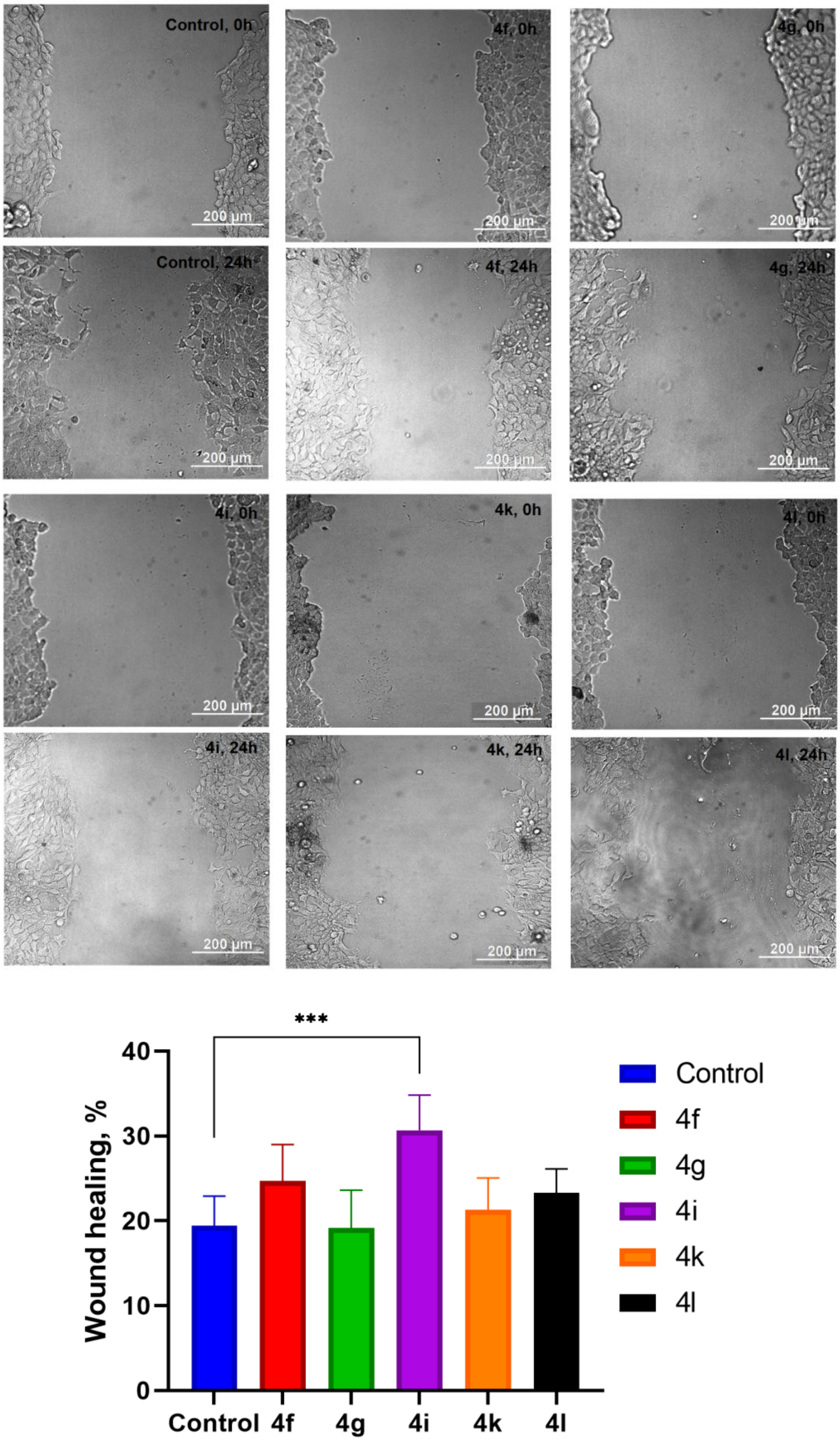

Figure S80. Microscopic images of the Sk-mel-2 cell wound area in the scratch assay after 24 h incubation with compounds **4f**, **4g**, **4i**, **4k**, **4l**

Table S6. Wound healing ability of Sk-mel-2 cells after incubation with compounds **4f**, **4g**, **4i**, **4k**, **4l**

	W (0h) ± SD, um	W (24h) ± SD, um	Wound close ± SD, %
Control	490 ± 42	395 ± 21	19 ± 7
**4f**	454 ± 17	342 ± 45	25 ± 9
**4g**	502 ± 41	406 ± 48	19 ± 10
**4i**	536 ± 32	372 ± 38	31 ± 8 *
**4k**	520 ± 46	409 ± 19	21 ± 7
**4l**	558 ± 45	428 ± 15	23 ± 6

W (0 h) – scratch width at 0 h, W (24 h) – scratch width at 24 h, wound close – 100 - W(0)/W(24)*100

Table S7. Docked study results of **4f, 4g, 4i, 4k, 4l** with the target protein (PDB ID: 8DNH)

**#**	**Protein PDB ID**	**Ligand**	**MolDock Score** [a]	**Rerank Score** [a]	**HBond** [a]	**MolDock Score** [a]	**Rerank Score** [a]	**HBond** [a]
			nucleotide cleft			target-binding cleft		
1	8DNH	**4a**	-118.39	-93.818	-8.16	-96.984	-77.745	-1.06
2	8DNH	**4b**	-146.56	-117.55	-9.45	-122.86	-87.129	-6.54
3	8DNH	**4c**	-156.54	-125.64	-9.11	-131.58	-86.951	-4.28
4	8DNH	**4d**	-155.24	-119.12	-7.56	-116.28	-84.209	-4.32
5	8DNH	**4e**	-153.27	-112.32	-8.21	-125.43	-90.405	-2.27
6	8DNH	**4f**	-146.55	-110.48	-8.77	-115.54	-81.125	-1.75
7	8DNH	**4g**	-154.53	-114.50	-8.33	-129.86	-93.329	-2.33
8	8DNH	**4h**	-146.02	-108.50	-8.67	-145.33	-108.60	-8.53
9	8DNH	**4i**	-147.82	-106.58	-3.78	-125.43	-91.830	-3.14
10	8DNH	**4j**	-141.08	-100.04	-7.27	-125.71	-83.003	-3.58
11	8DNH	**4k**	-154.92	-124.23	-6.19	-129.19	-101.33	-11.18
12	8DNH	**4l**	-118.39	-93.818	-8.16	-96.984	-77.745	-1.06
13	8DNH	**4m**	-146.56	-117.55	-9.45	-122.86	-87.129	-6.54
14	8DNH	**4n**	-156.54	-125.64	-9.11	-131.58	-86.951	-4.28
15	8DNH	**4o**	-155.23	-119.12	-7.56	-116.28	-84.209	-4.32
16	8DNH	**4p**	-153.27	-112.33	-8.21	-125.43	-90.405	-2.27
17	8DNH	ADP	-154.53	-114.50	-8.33	-129.86	-93.329	-2.33

[a] arbitrary units; ADP – adenosine diphosphate
